# Supplementary material for: A design principle of polymers processable into 2D homeotropic order
Source: Nat Commun. 2016 Nov 29;7:13640. doi: 10.1038/ncomms13640 (PMC5141351; doi:10.1038/ncomms13640)
Supplement: Supplementary Information — Supplementary Figures 1-34, Supplementary Tables 1-16, Supplementary Discussion, Supplementary Methods, and Supplementary References [file ncomms13640-s1.pdf]

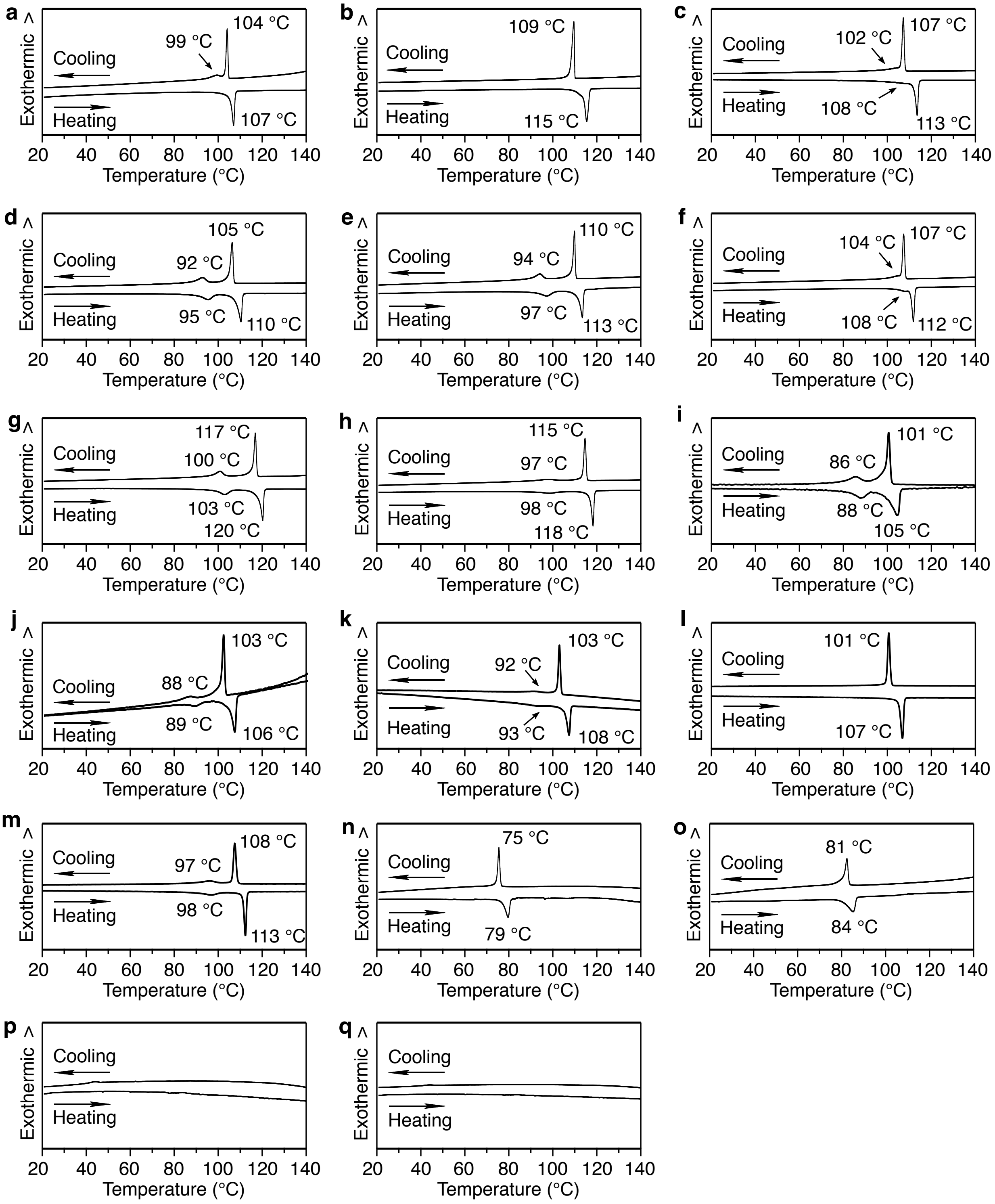

Supplementary Figure 1. DSC traces at a scan rate of 5 °C min^{-1} in the second heating/cooling cycle of **(a) PMA$^{\mathbf{BBB}}$**, (**b**) **PA$^{\mathbf{BBB}}$**, (**c**) **PPA$^{\mathbf{BBB}}$**, (**d**) **PMA$^{\mathbf{TTT}}$**, (**e**) **PMA$^{\mathbf{TTA}}$**, (**f**) **PMA$^{\mathbf{BBA}}$**, (**g**) **PMA$^{\mathbf{TAA}}$**,

(**h**) **PMA**$^{\mathbf{BAA}}$, (**i**) **PMA**$^{\mathbf{TTB}}$, (**j**) **PMA**$^{\mathbf{TBT}}$, (**k**) **PMA**$^{\mathbf{BTT}}$, (**l**) **PMA**$^{\mathbf{BBB'}}$, (**m**) **PMA**$^{\mathbf{TTT'}}$, (**n**) **PMA**$^{\mathbf{BB}}$, (**o**) **PMA**$^{\mathbf{TT}}$, (**p**) **PMA**$^{\mathbf{B}}$ and (**q**) **PMA**$^{\mathbf{T}}$.

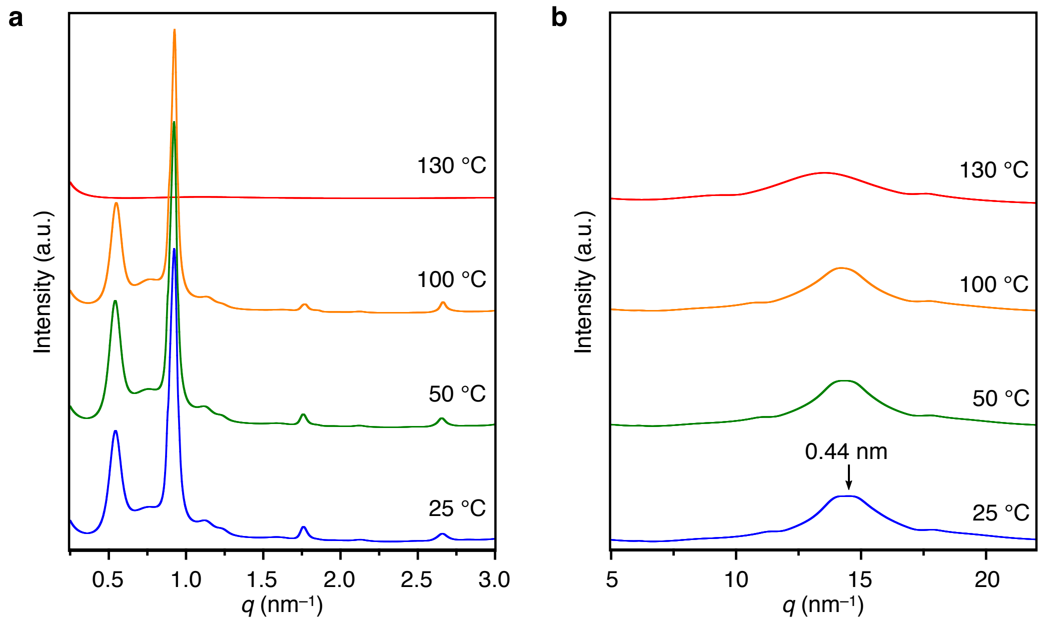

Supplementary Figure 2. (**a**) SAXS and (**b**) WAXD patterns of a bulk sample of **PMA**$^{\mathbf{BBB}}$ at different temperatures on cooling from its isotropic melt in a glass capillary (ϕ = 1.5 mm).

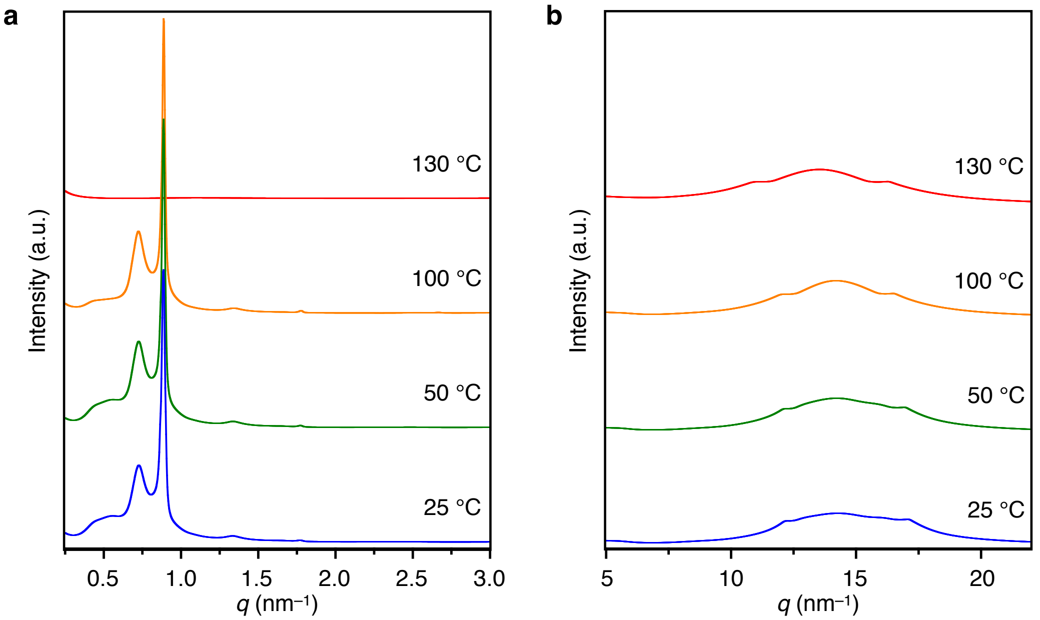

Supplementary Figure 3. (**a**) SAXS and (**b**) WAXD patterns of a bulk sample of **PMA**$^{\mathbf{BAA}}$ at different temperatures on cooling from its isotropic melt in a glass capillary (ϕ = 1.5 mm).

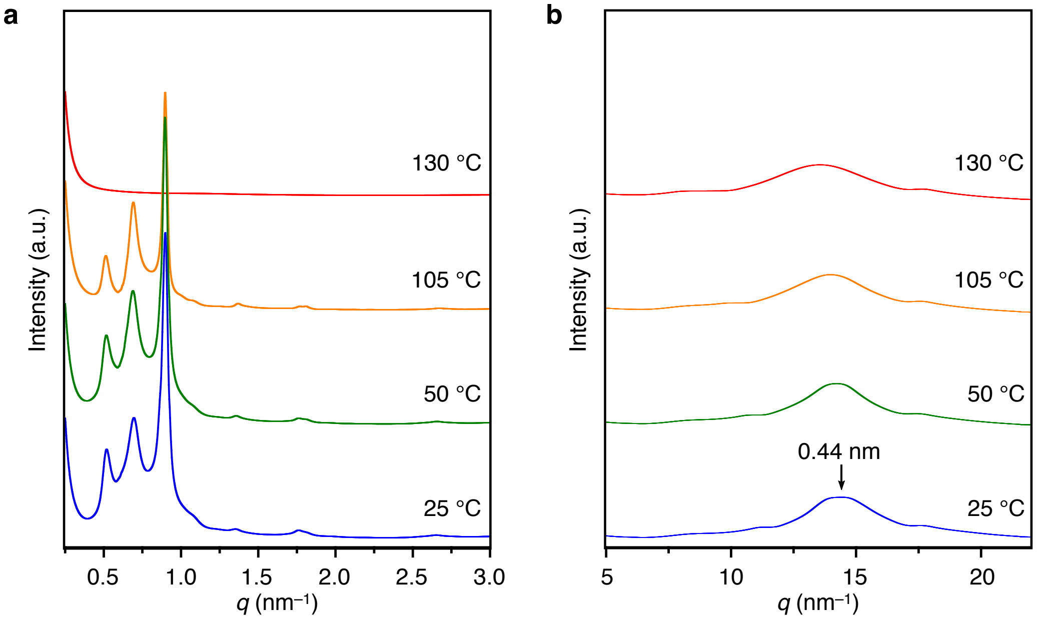

Supplementary Figure 4. (**a**) SAXS and (**b**) WAXD patterns of a bulk sample of **PMA**$^{\mathbf{BBA}}$ at different temperatures on cooling from its isotropic melt in a glass capillary (ϕ = 1.5 mm).

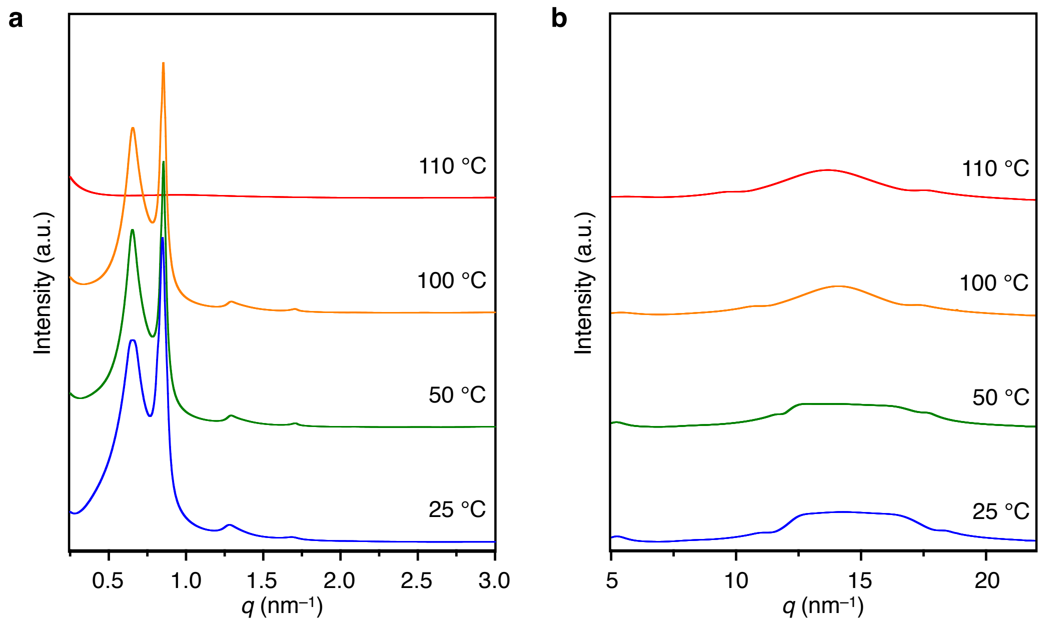

Supplementary Figure 5. (**a**) SAXS and (**b**) WAXD patterns of a bulk sample of **PMA**$^{\mathbf{TTT}}$ at different temperatures on cooling from its isotropic melt in a glass capillary ($\phi = 1.5$ mm).

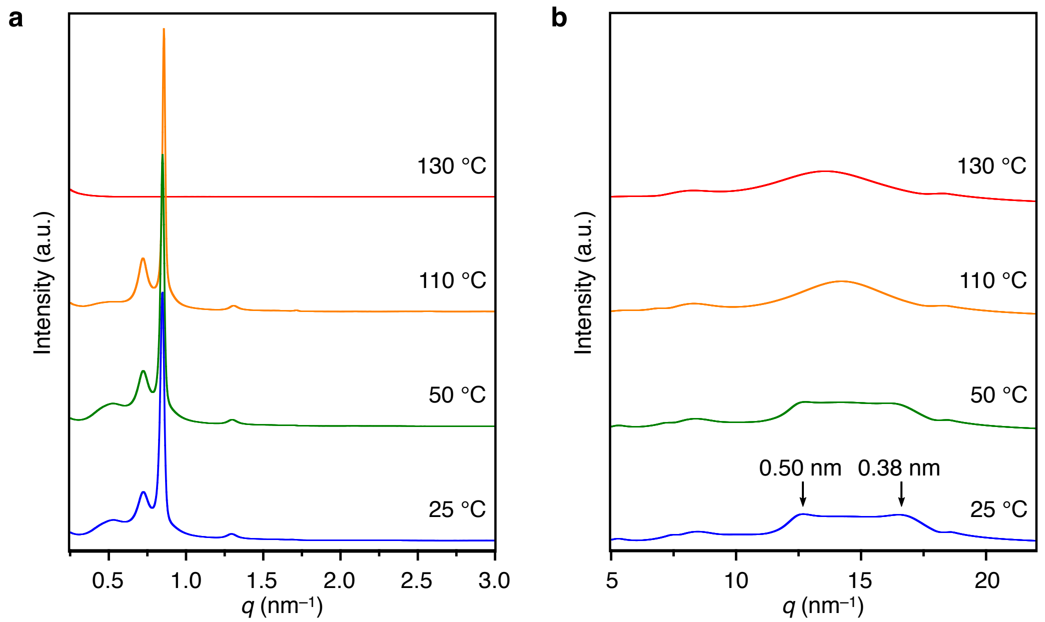

Supplementary Figure 6. (**a**) SAXS and (**b**) WAXD patterns of a bulk sample of **PMA**$^{\mathbf{TAA}}$ at different temperatures on cooling from its isotropic melt in a glass capillary ($\phi = 1.5$ mm).

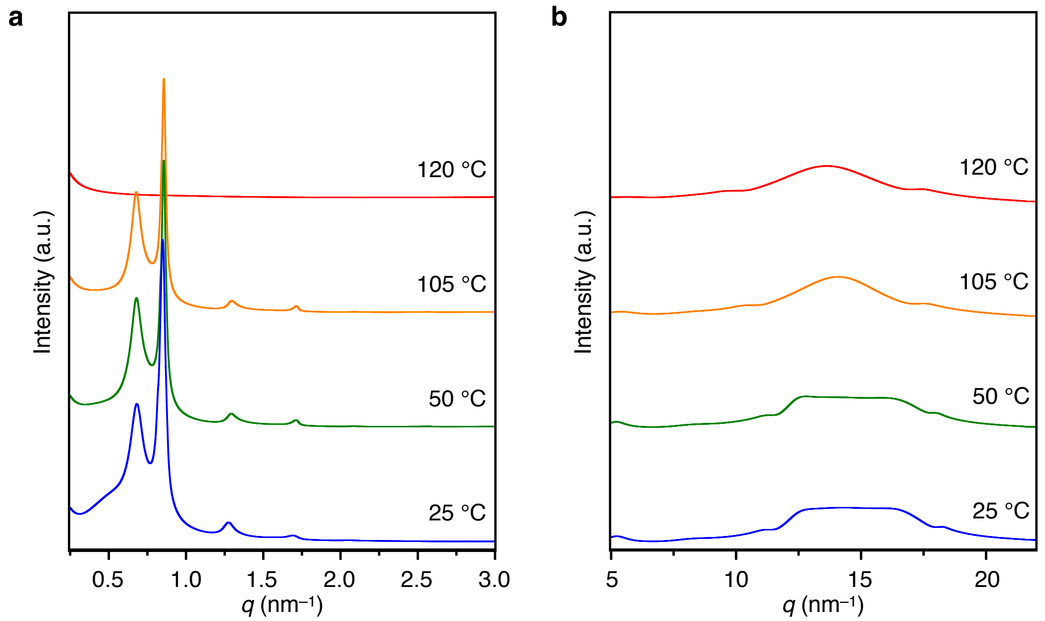

Supplementary Figure 7. (**a**) SAXS and (**b**) WAXD patterns of a bulk sample of **PMA**^{TTA} at different temperatures on cooling from its isotropic melt in a glass capillary (ϕ = 1.5 mm).

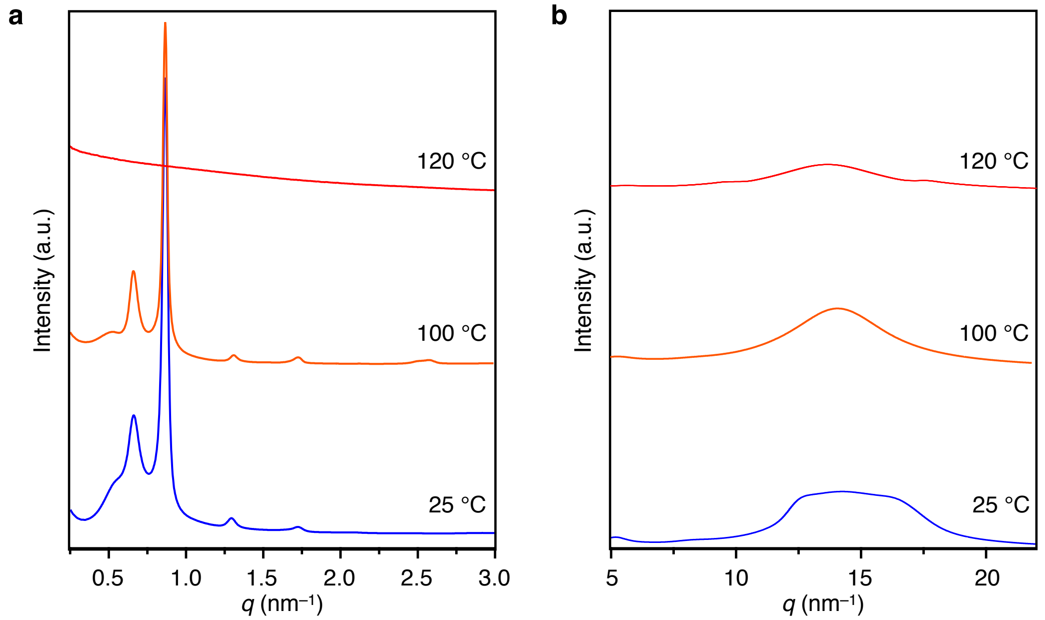

Supplementary Figure 8. (**a**) SAXS and (**b**) WAXD patterns of a bulk sample of **PMA**^{TTB} at different temperatures on cooling from its isotropic melt in a glass capillary (ϕ = 1.5 mm).

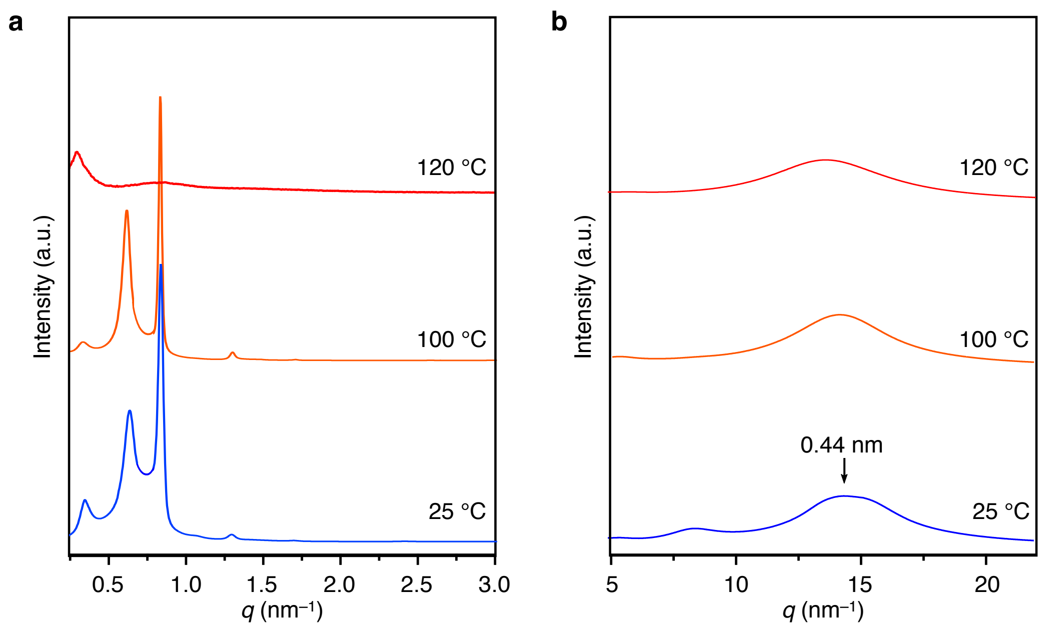

Supplementary Figure 9. (**a**) SAXS and (**b**) WAXD patterns of a bulk sample of **PMA**$^{\mathbf{TBT}}$ at different temperatures on cooling from its isotropic melt in a glass capillary ($\phi = 1.5$ mm).

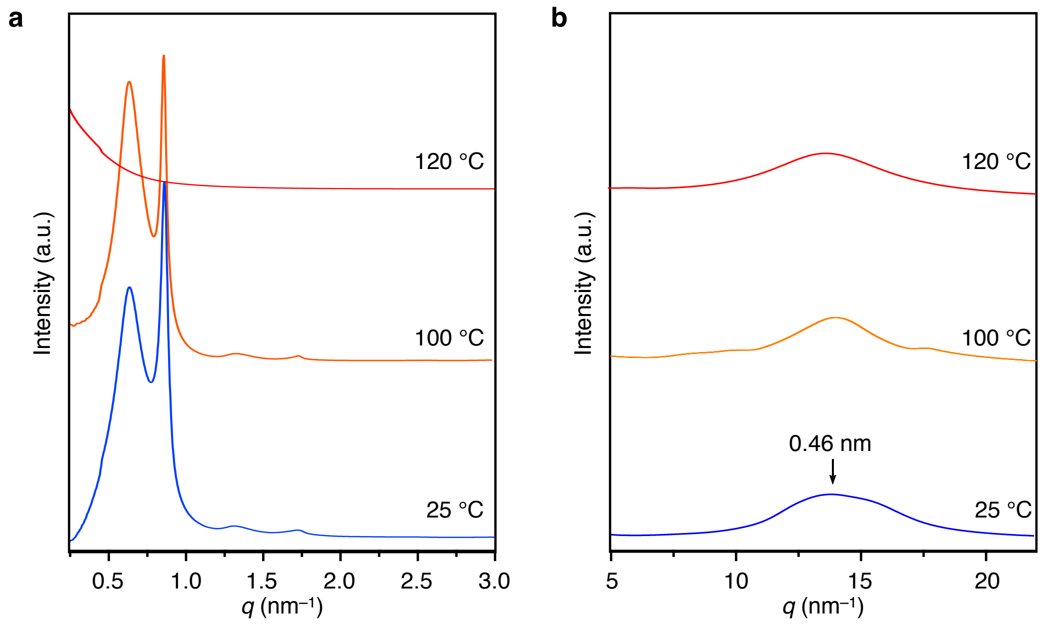

Supplementary Figure 10. (**a**) SAXS and (**b**) WAXD patterns of a bulk sample of **PMA**$^{\mathbf{BTT}}$ at different temperatures on cooling from its isotropic melt in a glass capillary ($\phi = 1.5$ mm)

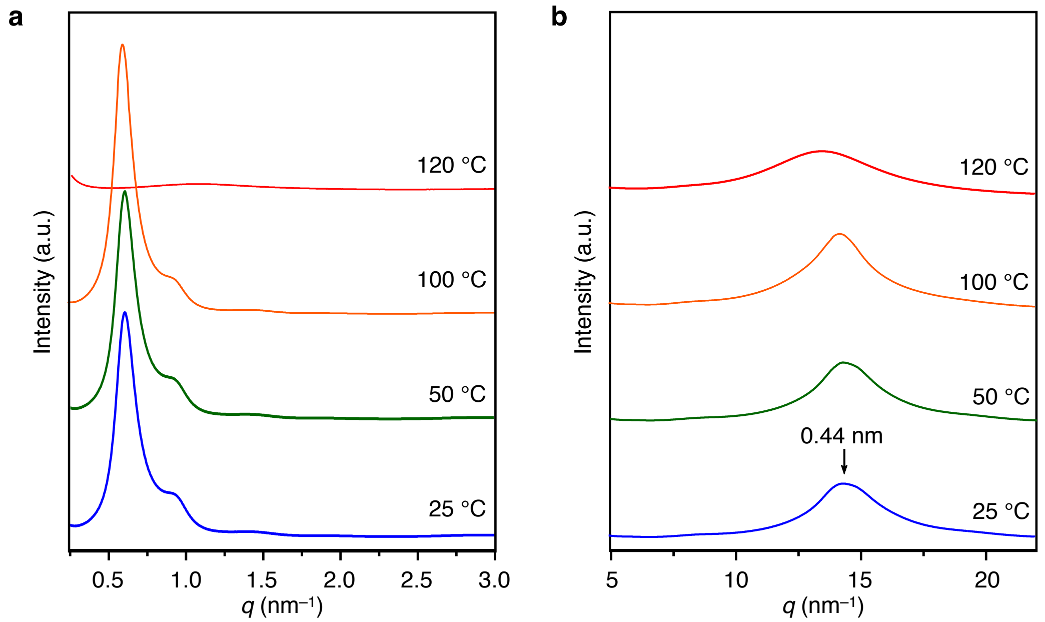

Supplementary Figure 11. (**a**) SAXS and (**b**) WAXD patterns of a bulk sample of **PMA**$^{\mathbf{BBB'}}$ at different temperatures on cooling from its isotropic melt in a glass capillary ($\phi = 1.5$ mm).

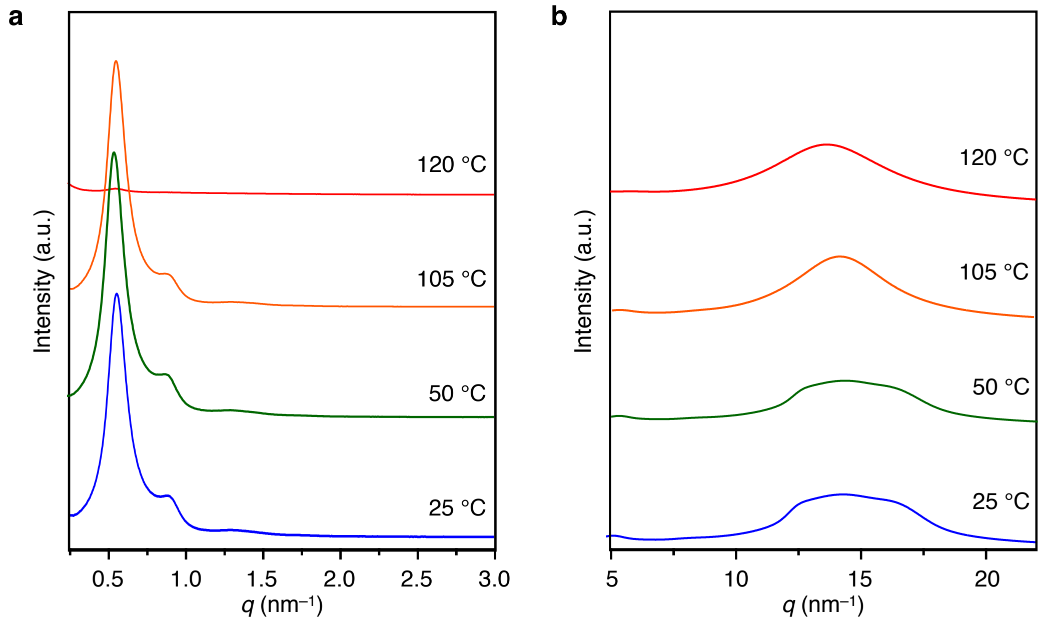

Supplementary Figure 12. (**a**) SAXS and (**b**) WAXD patterns of a bulk sample of **PMA**$^{\mathbf{TTT'}}$ at different temperatures on cooling from its isotropic melt in a glass capillary ($\phi = 1.5$ mm).

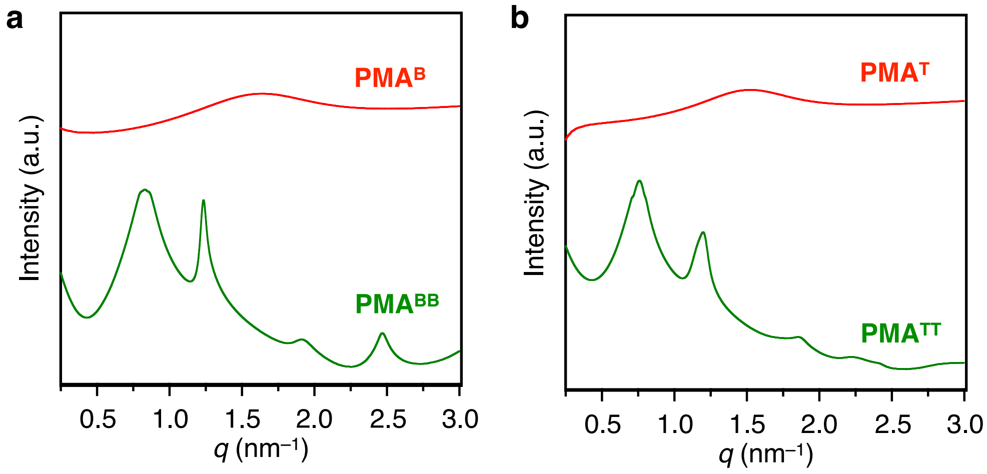

Supplementary Figure 13. SAXS patterns of bulk samples of (**a**) **PMA^{B}** (red) and **PMA^{BB}** (green) at 25 °C, and (**b**) **PMA^{T}** (red) and **PMA^{TT}** (green) at 25 °C on cooling from their isotropic melts in a glass capillary (ϕ = 1.5 mm).

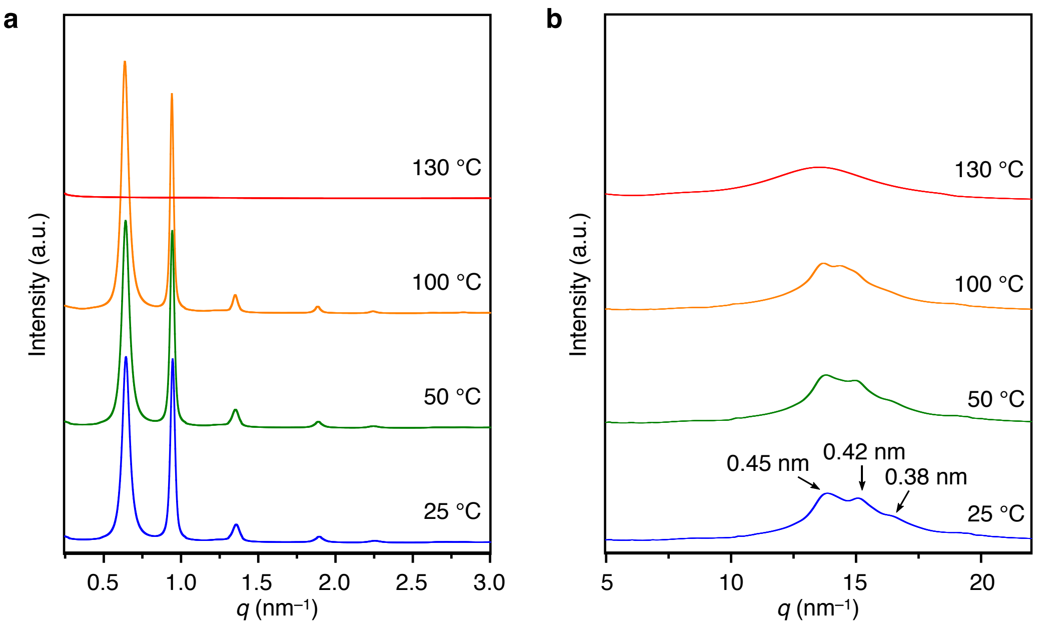

Supplementary Figure 14. (**a**) SAXS and (**b**) WAXD patterns of a bulk sample of **PA^{BBB}** at different temperatures on cooling from its isotropic melt in a glass capillary (ϕ = 1.5 mm).

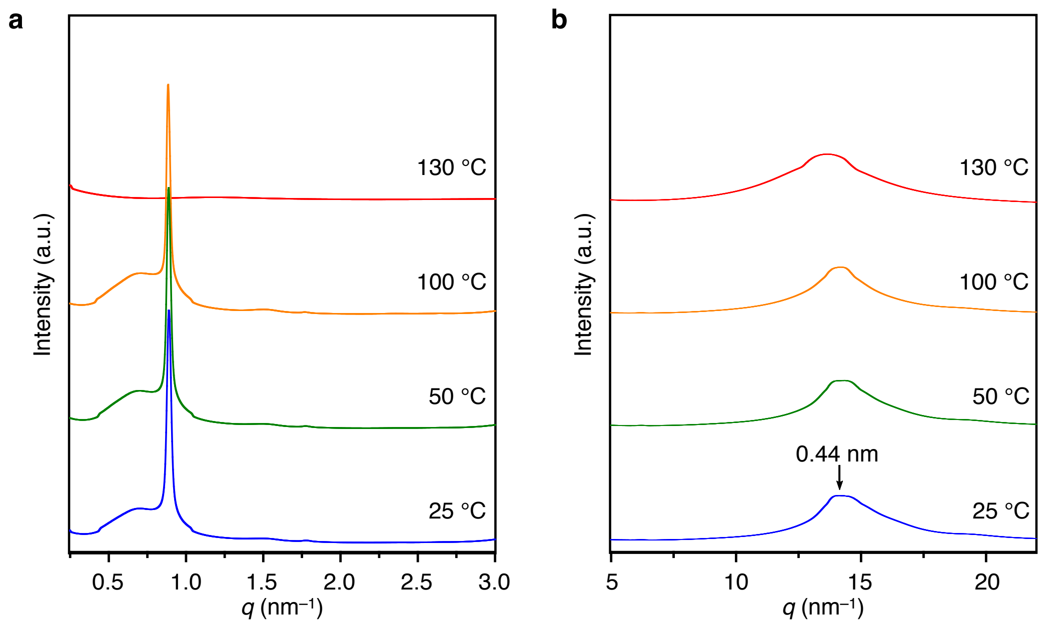

Supplementary Figure 15. (**a**) SAXS and (**b**) WAXD patterns of a bulk sample of **PPA**$^{\mathbf{BBB}}$ at different temperatures on cooling from its isotropic melt in a glass capillary (ϕ = 1.5 mm).

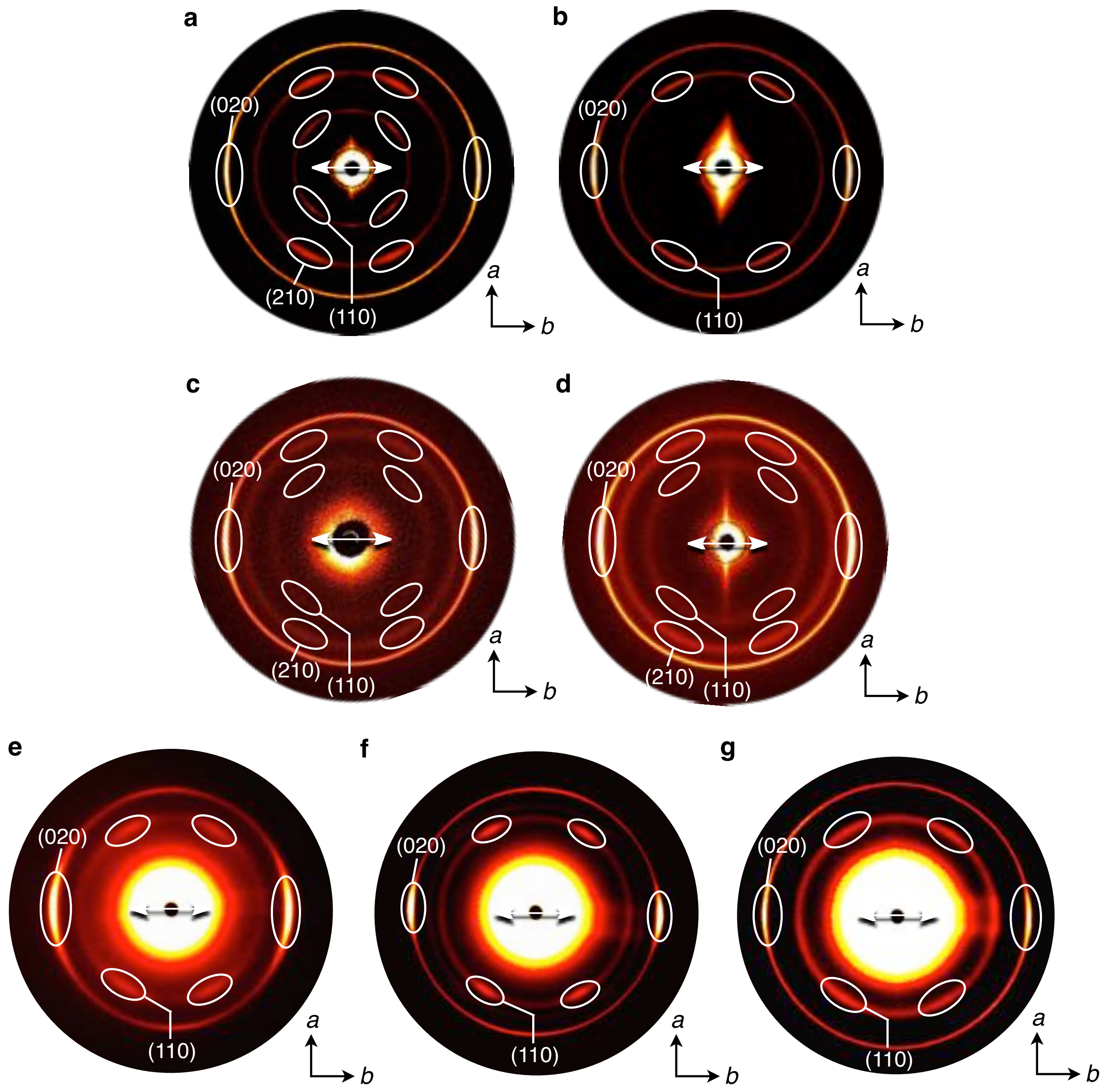

Supplementary Figure 16. Through-view 2D SAXS images at 25 °C of hot-pressed films of (**a**) **PMA**$^{\mathbf{BBA}}$, (**b**) **PMA**$^{\mathbf{TTA}}$, (**c**) **PMA**$^{\mathbf{BAA}}$, **(d) PMA**$^{\mathbf{TAA}}$, (**e**) **PMA**$^{\mathbf{TTB}}$, (**f**) **PMA**$^{\mathbf{TBT}}$ and **(g) PMA**$^{\mathbf{BTT}}$, prepared with two parallel Teflon sheets. White arrows represent the directions of the grooves on the Teflon sheets. Black arrows represent the orientations of the *a*- and *b*-axes of the 2D rectangular lattices of bottlebrush polymers.

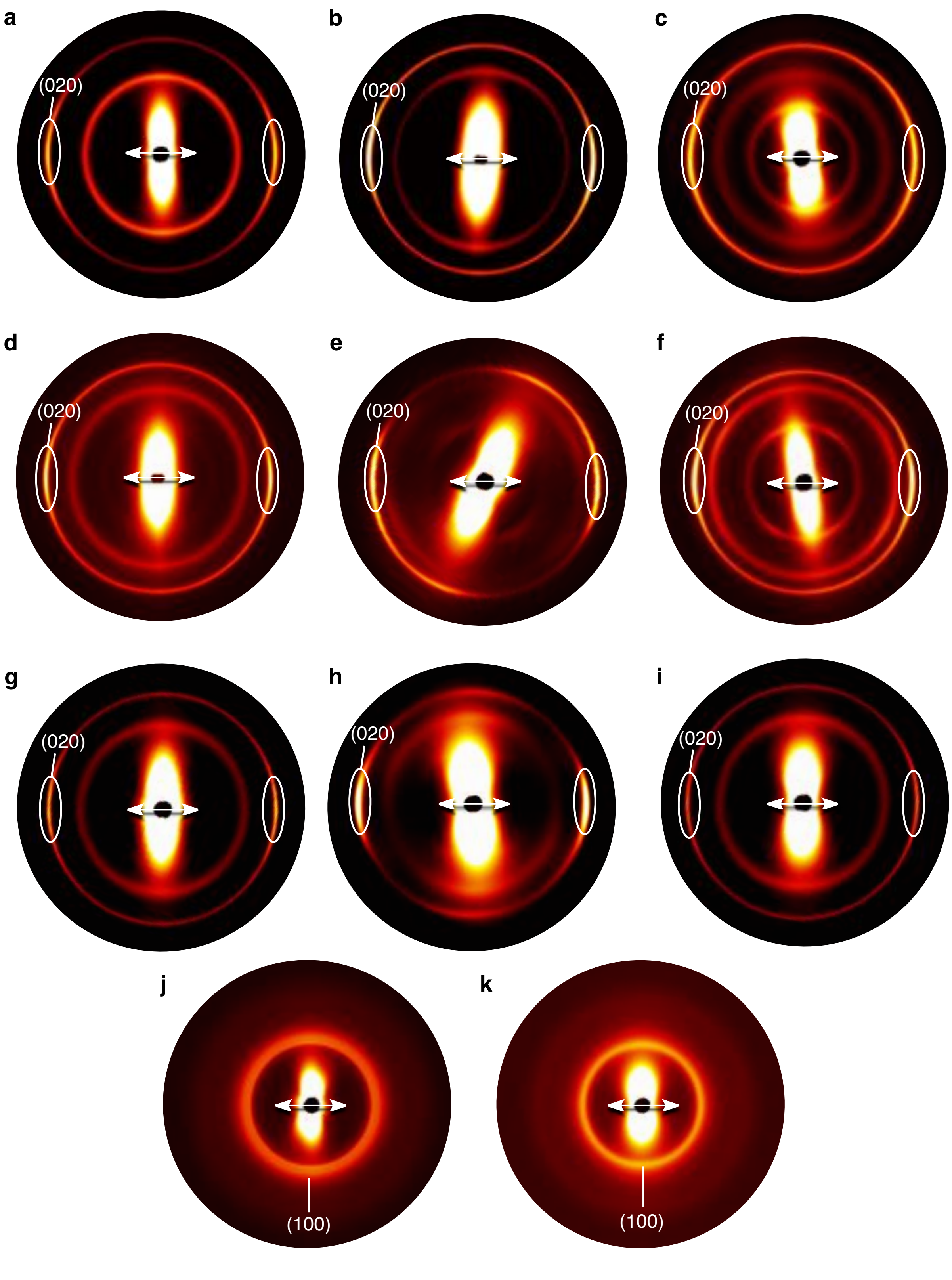
a
(020)
b
(020)
c
(020)
d
(020)
e
(020)
f
(020)
g
(020)
h
(020)
i
(020)
j
(100)
k
(100)

Supplementary Figure 17. Edge-view 2D SAXS images at 25 °C of hot-pressed films of (**a**) **PMA**^{BBB}, (**b**) **PMA**^{TTT}, (**c**) **PMA**^{BBA}, (**d**) **PMA**^{TTA}, (**e**) **PMA**^{TAA}, (**f**) **PMA**^{BAA}, (**g**) **PMA**^{TTB}, (**h**) **PMA**^{TBT}, (**i**) **PMA**^{BTT}, (**j**) **PMA**$^{BBB'}$ and (**k**) **PMA**$^{TTT'}$, prepared with two parallel Teflon sheets. White arrows represent the directions of the grooves on the Teflon sheets.

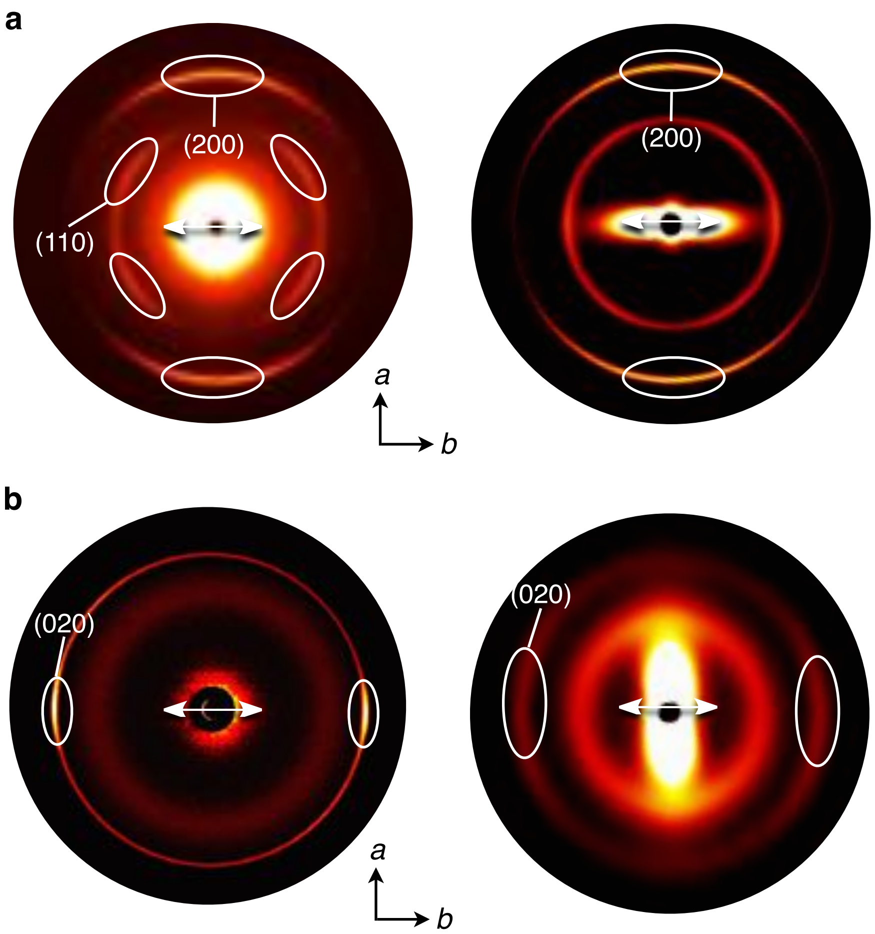

Supplementary Figure 18. Through-(left) and edge-view (right) 2D SAXS images at 25 °C of hot-pressed films of (**a**) **PA**^{BBB} and (**b**) **PPA**^{BBB} prepared with two parallel Teflon sheets. White arrows represent the directions of the grooves on the Teflon sheets. Black arrows represent the orientations of the *a*- and *b*-axes of the 2D rectangular lattices of bottlebrush polymers.

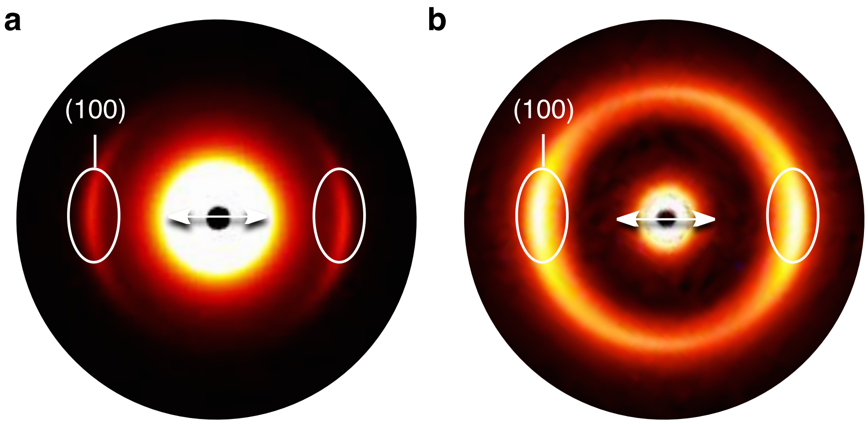

Supplementary Figure 19. Through-view 2D SAXS images at 25 °C of hot-pressed films of the monomers for (**a**) **PMA$^{\mathbf{BBB}}$** and (**b**) **PMA$^{\mathbf{TTT}}$** prepared with two parallel Teflon sheets. White arrows represent the directions of the grooves on the Teflon sheets.

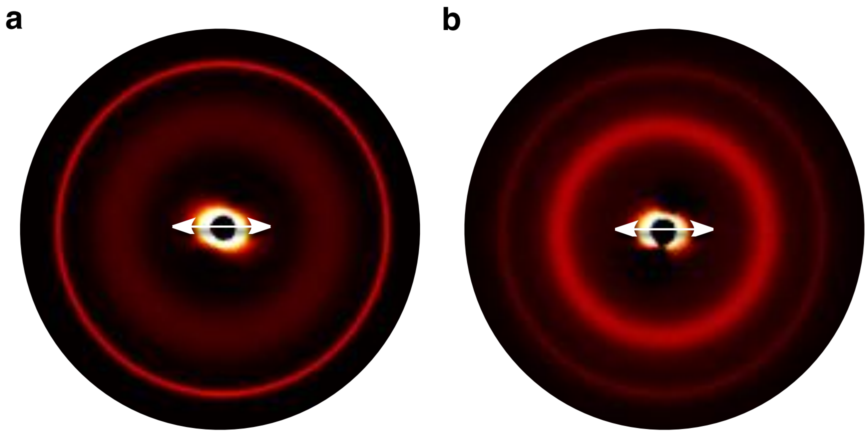

Supplementary Figure 20. Through-view 2D SAXS images at 25 °C of thermally annealed films of (**a**) **PMA$^{\mathbf{BBB}}$** and (**b**) **PMA$^{\mathbf{TTT}}$** on a Teflon sheet after drop-casting without pressing. White arrows represent the directions of the grooves on the Teflon sheets.

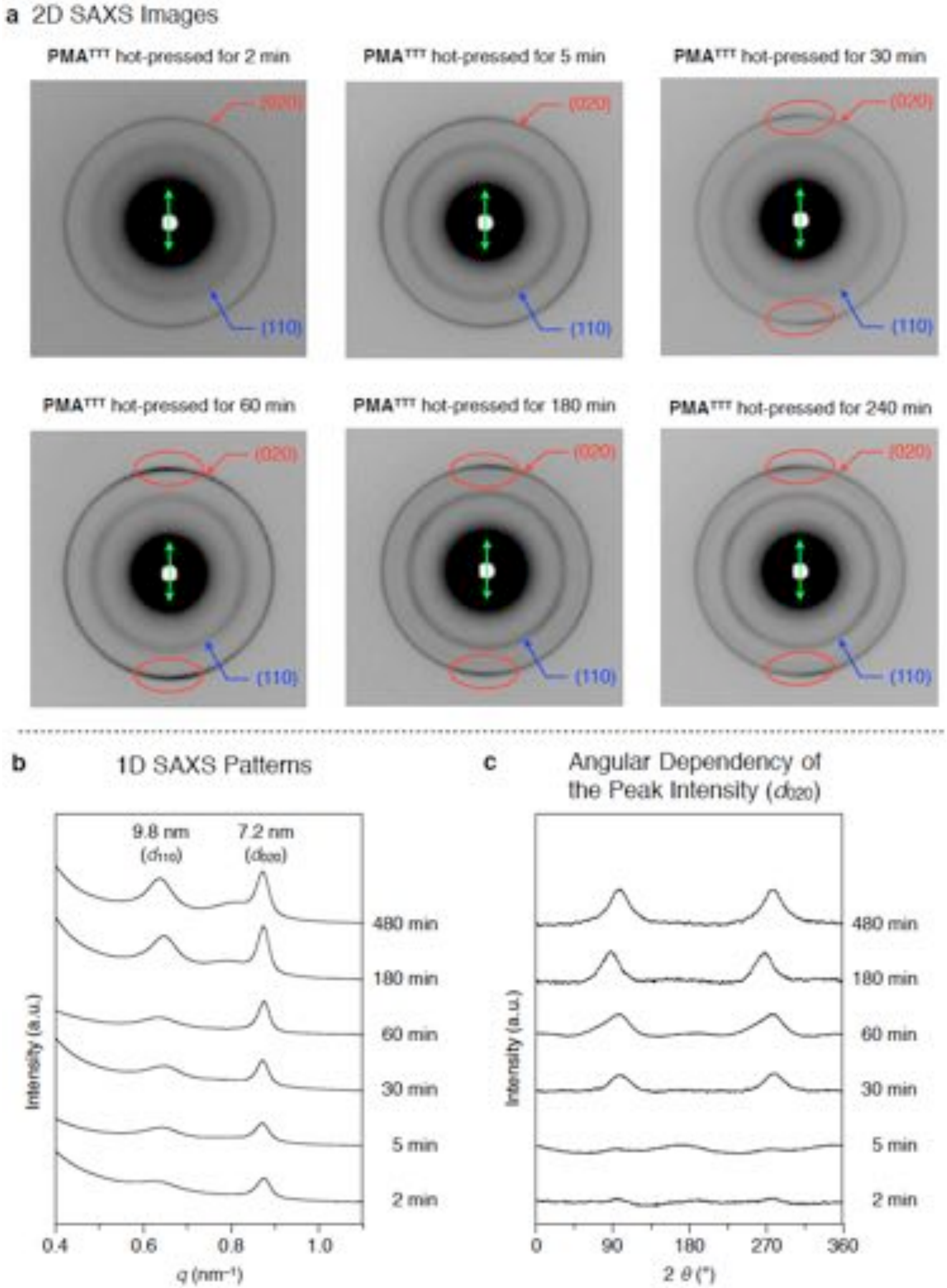

Supplementary Figure 21. (**a**) Through-view 2D SAXS images of thin films of **PMA^{TTT}** hot-pressed for 2, 5, 30, 60, 180 and 480 min. Green arrows represent the directions of the grooves on the Teflon sheets. 1D SAXS patterns (**b**) and angular dependency (**c**) of the peak intensity of the diffraction from the (020) plane converted from the SAXS images of the films.

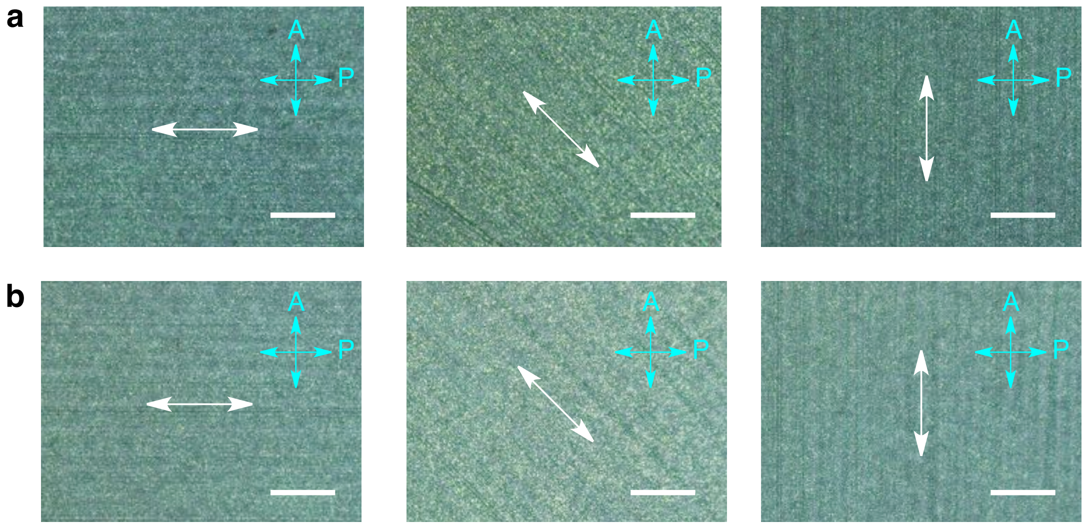

Supplementary Figure 22. POM micrographs (scale bars = 100 μm) at 25 °C under crossed polarizers of hot-pressed films of (**a**) **PMA**$^{\mathbf{BBB'}}$ and (**b**) **PMA**$^{\mathbf{TTT'}}$ prepared with parallel Teflon sheets. The films were tilted at 0° (left), 45° (center) and 90° (right) relative to the transmission axis of the analyzer. Sky-blue arrows represent the transmission axes of the polarizer (P) and analyzer (A), whereas white arrows represent the directions of the grooves on the Teflon sheets.

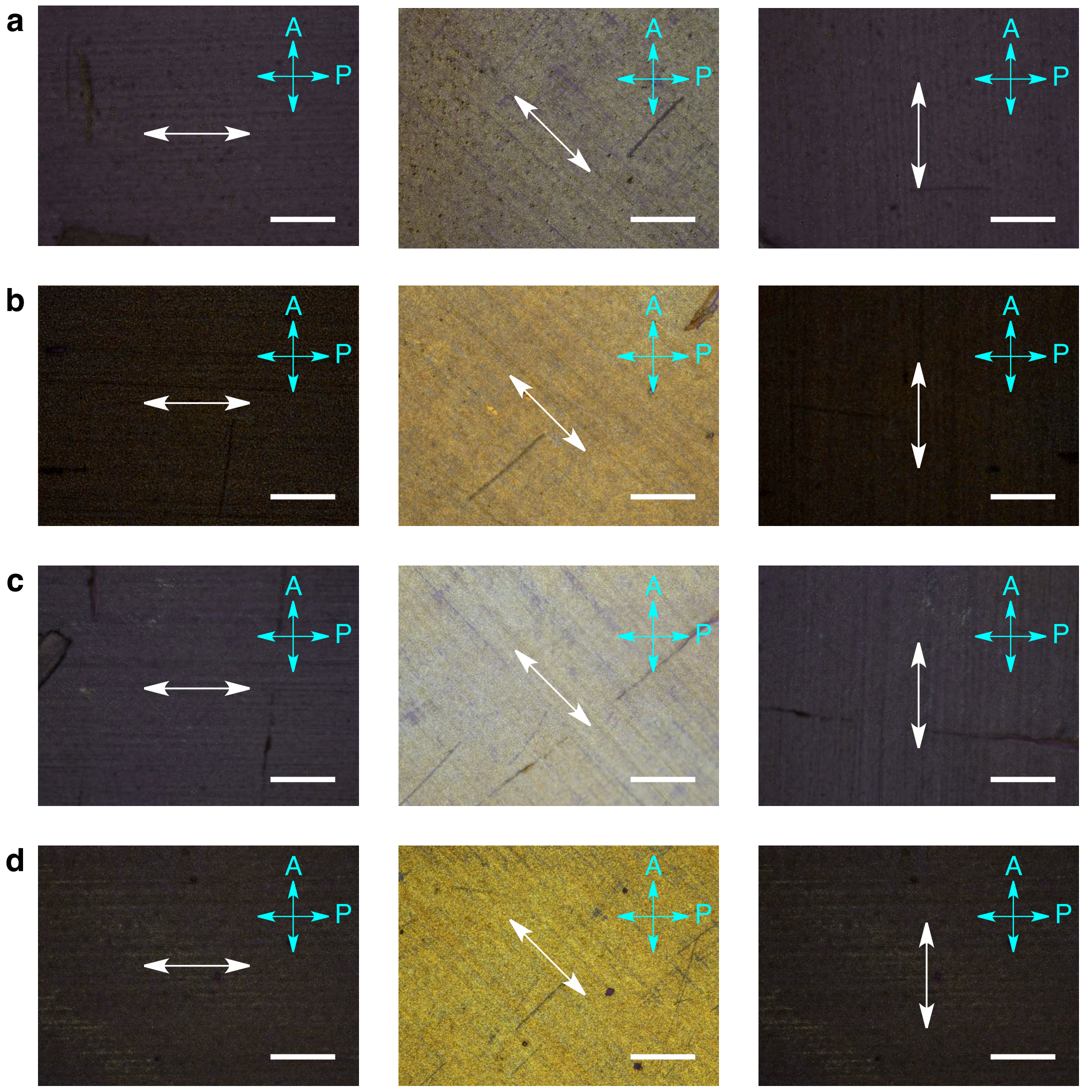

Supplementary Figure 23. POM micrographs (scale bars = 100 μm) at 25 °C under crossed polarizers of hot-pressed films of (**a**) **PMA**$^{\mathbf{BBA}}$, (**b**) **PMA**$^{\mathbf{BAA}}$, (**c**) **PMA**$^{\mathbf{TTA}}$ and (**d**) **PMA**$^{\mathbf{TAA}}$ prepared with parallel Teflon sheets. The films were tilted at 0° (left), 45° (center) and 90° (right) relative to the transmission axis of the analyzer. Sky-blue arrows represent the transmission axes of the polarizer (P) and analyzer (A), whereas white arrows represent the directions of the grooves on the Teflon sheets.

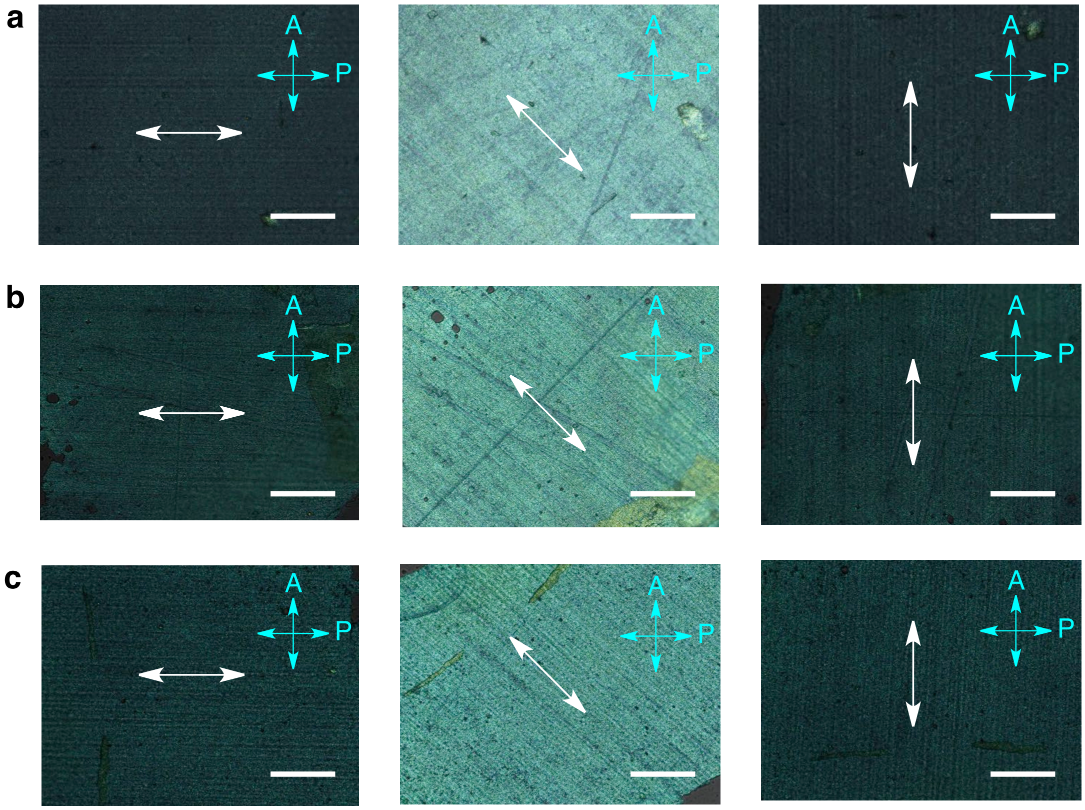

Supplementary Figure 24. POM micrographs (scale bars = 100 μm) at 25 °C under crossed polarizers of hot-pressed films of (**a**) **PMA^{TTB}**, (**b**) **PMA^{TBT}** and (**c**) **PMA^{BTT}** prepared with parallel Teflon sheets. The films were tilted at 0° (left), 45° (center) and 90° (right) relative to the transmission axis of the analyzer. Sky-blue arrows represent the transmission axes of the polarizer (P) and analyzer (A), whereas white arrows represent the directions on the grooves of the Teflon sheets.

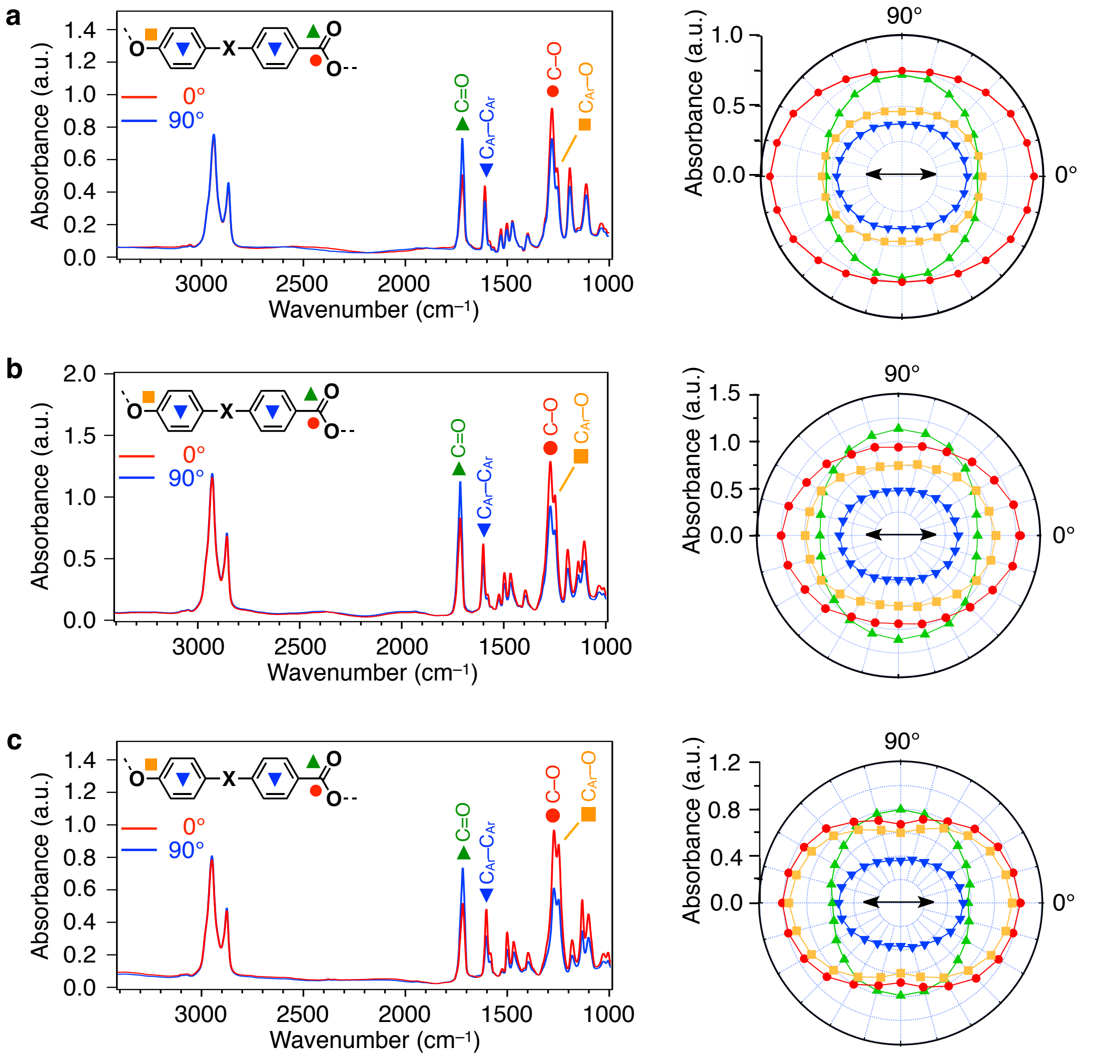

Supplementary Figure 25. Polarized IR spectra and polar plots of IR absorption intensities, recorded upon rotation of a polarizer every 15°, of hot-pressed films of (**a**) **PMA^{BBB}**, (**b**) **PMA^{BBA}** and (**c**) **PMA^{BAA}** prepared with parallel Teflon sheets, at azimuthal angles $\theta = 0°$ (red line) and 90° (blue line). The azimuthal angle is defined as zero when the polarizing direction of the incident light is parallel to the directions of the grooves on the Teflon sheets (black arrows). Stretching vibrations at 1714 (green triangle), 1604 (blue triangle), 1277 (red closed circle) and 1245 cm^{-1} (yellow square) are attributable to C=O (ester carbonyl), C_{Ar}–C_{Ar} (aromatic), C–O (ester) and C_{Ar}–O (ether) groups, respectively.

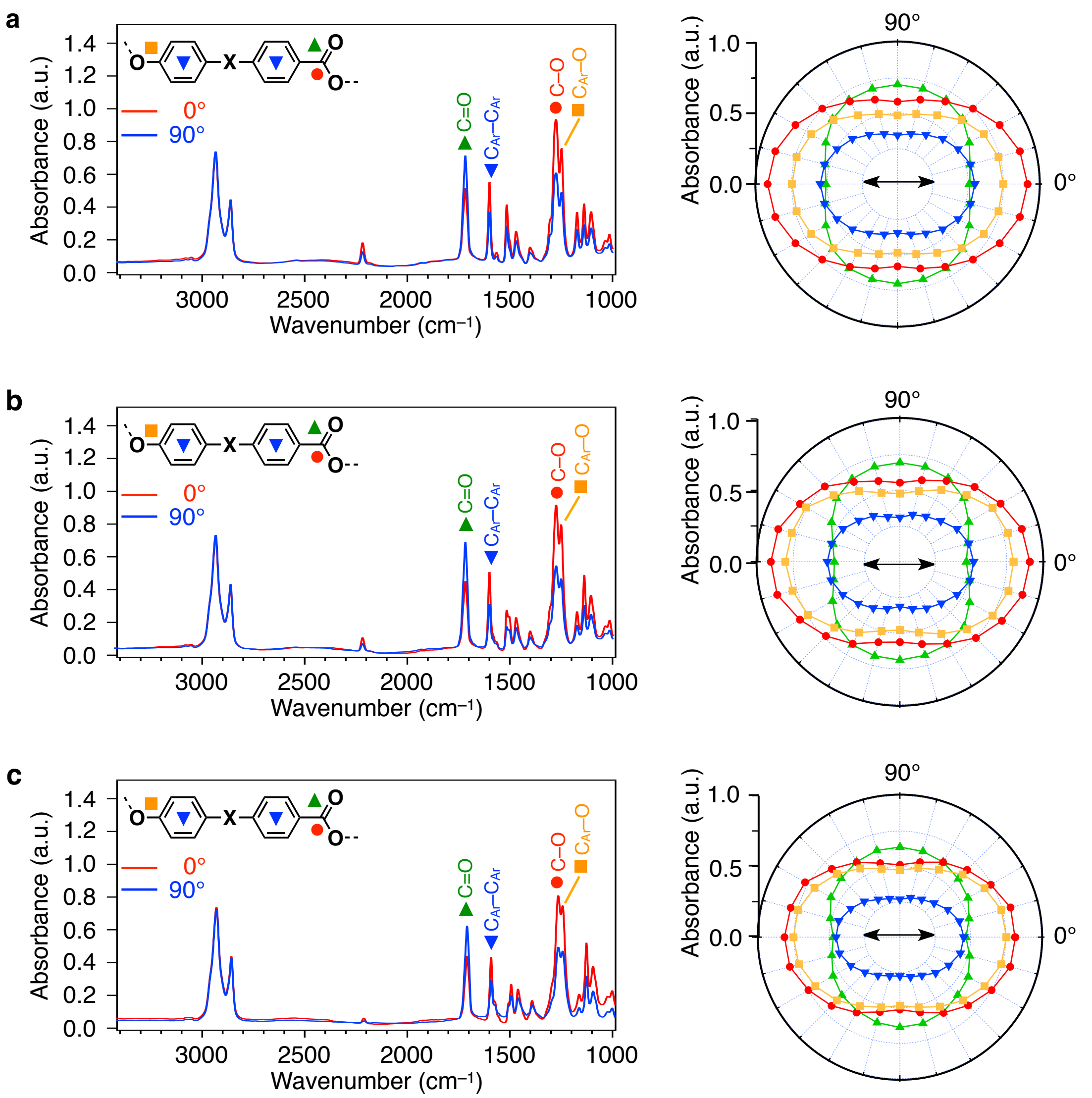

Supplementary Figure 26. Polarized IR spectra and polar plots of IR absorption intensities, recorded upon rotation of a polarizer every 15°, of hot-pressed films of (**a**) **PMA^TTT**, (**b**) **PMA^TTA** and (**c**) **PMA^TAA** prepared with parallel Teflon sheets at azimuthal angles $\theta = 0°$ (red line) and 90° (blue line). The azimuthal angle is defined as zero when the polarizing direction of the incident light is parallel to the directions of the grooves of the Teflon sheets (black arrows). Stretching vibrations at 1714 (green triangle), 1604 (blue triangle), 1277 (red closed circle) and 1245 cm^{-1} (yellow square) are attributable to C=O (ester carbonyl), C_{Ar}–C_{Ar} (aromatic), C–O (ester) and C_{Ar}–O (ether) groups, respectively.

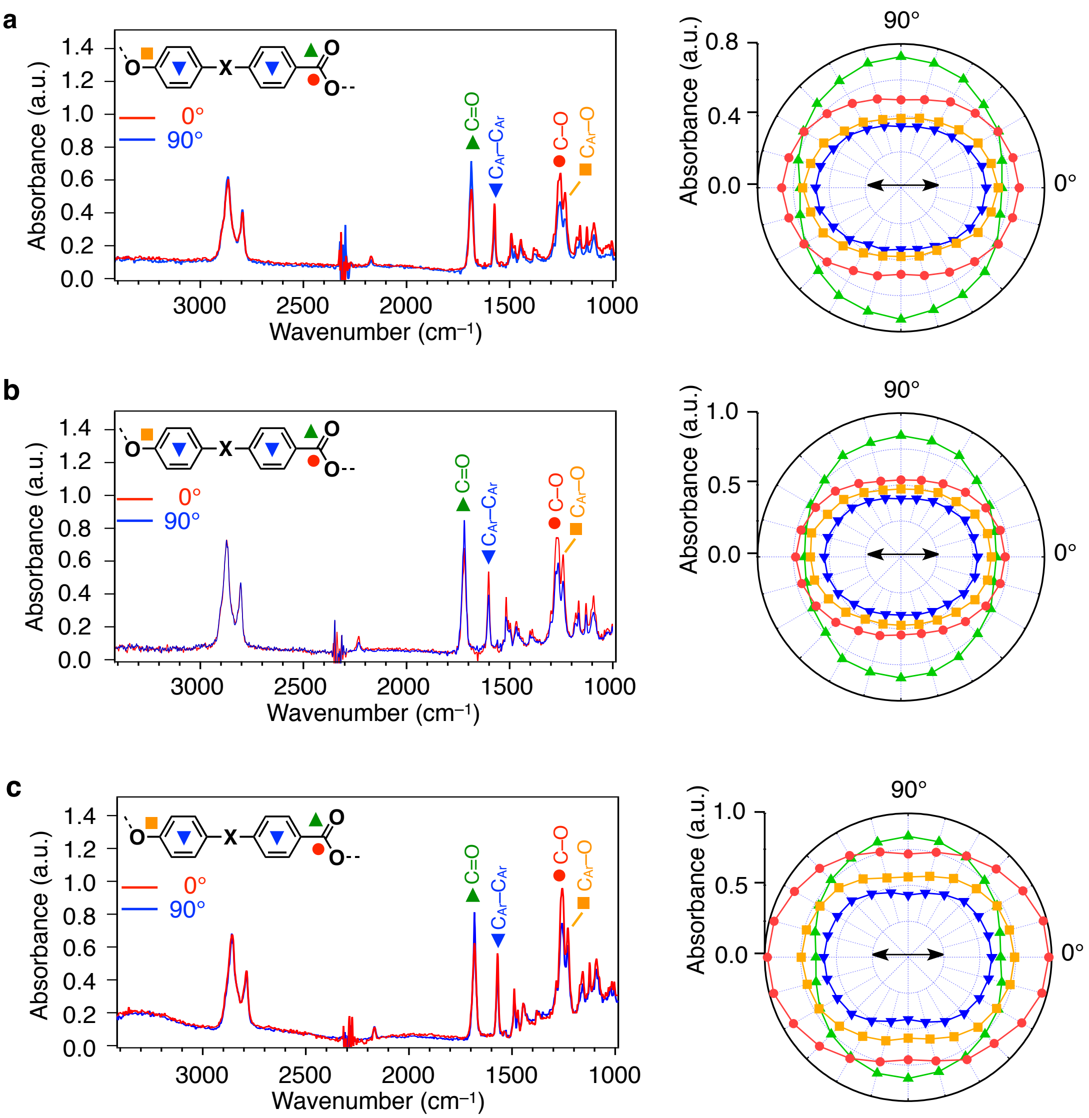

Supplementary Figure 27. Polarized IR spectra and polar plots of IR absorption intensities, recorded upon rotation of a polarizer every 15°, of hot-pressed films of (**a**) **PMA$^{\mathbf{TTB}}$**, (**b**) **PMA$^{\mathbf{TBT}}$** and (**c**) **PMA$^{\mathbf{TTB}}$** prepared with parallel Teflon sheets at azimuthal angles $\theta = 0°$ (red line) and 90° (blue line). The azimuthal angle is defined as zero when the polarizing direction of the incident light is parallel to the directions of the grooves of the Teflon sheets (black arrows). Stretching vibrations at 1714 (green triangle), 1600 (blue triangle), 1277 (red closed circle) and 1245 cm^{-1} (yellow square) are attributable to C=O (ester carbonyl), C_{Ar}–C_{Ar} (aromatic), C–O (ester) and C_{Ar}–O (ether) groups, respectively.

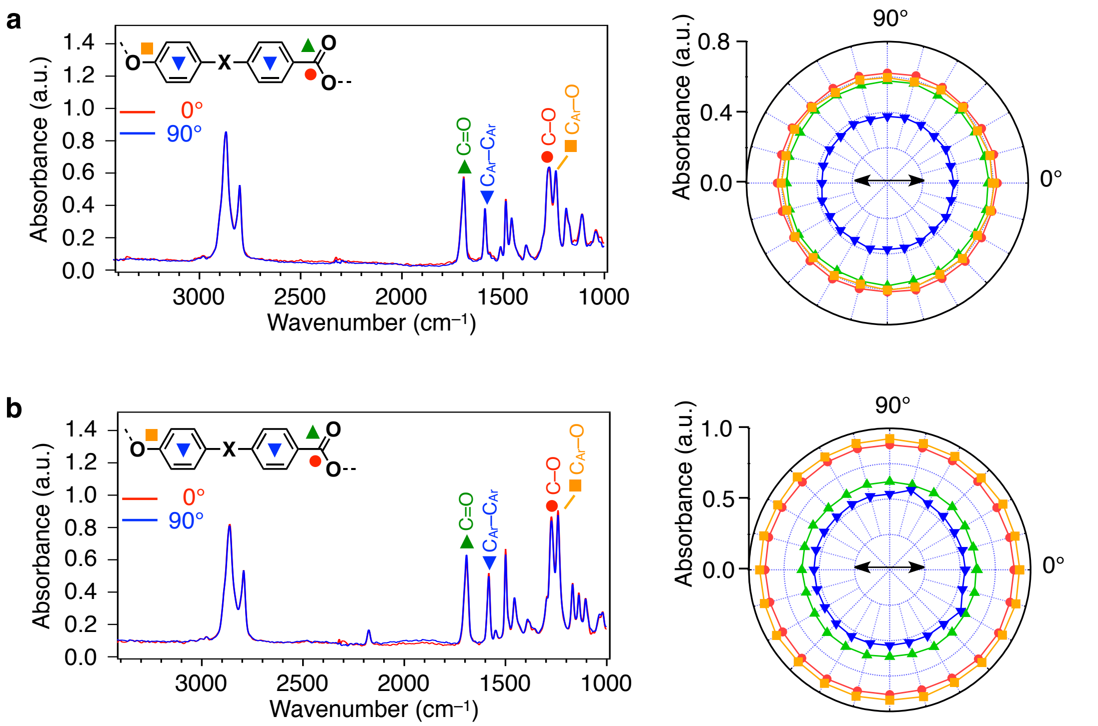

Supplementary Figure 28. Polarized IR spectra and polar plots of IR absorption intensities, recorded upon the rotation of a polarizer every 15°, of hot-pressed films of (**a**) **PMA$^{BBB'}$** and (**b**) **PMA$^{TTT'}$** prepared with parallel Teflon sheets at azimuthal angles $\theta = 0°$ (red line) and 90° (blue line). The azimuthal angle is defined as zero when the polarizing direction of the incident light is parallel to the direction of the grooves of the Teflon sheets (black arrows). Stretching vibrations at 1714 (green triangle), 1600 (blue triangle), 1274 (red closed circle) and 1249 cm^{-1} (yellow square) are attributable to C=O (ester carbonyl), C_{Ar}–C_{Ar} (aromatic), C–O (ester) and C_{Ar}–O (ether) groups, respectively.

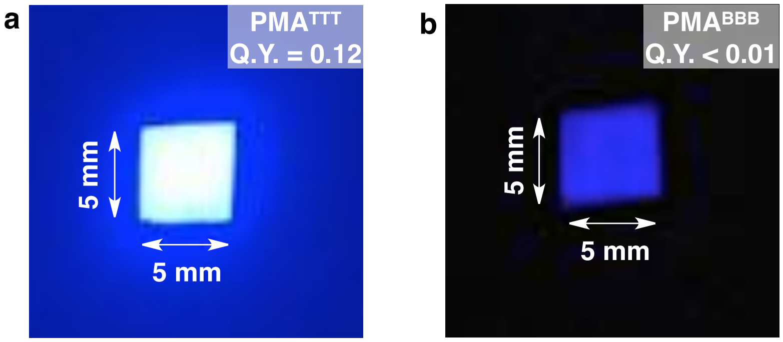

Supplementary Figure 29. Photographs of hot-pressed films (5 mm × 5 mm) of (**a**) **PMA**$^{\mathbf{TTT}}$ and (**b**) **PMA**$^{\mathbf{BBB}}$ irradiated by UV light (365 nm). The fluorescence quantum yield of hot-pressed **PMA**$^{\mathbf{TTT}}$ film was 0.12, while that of hot-pressed **PMA**$^{\mathbf{BBB}}$ flim was only less than 0.01.

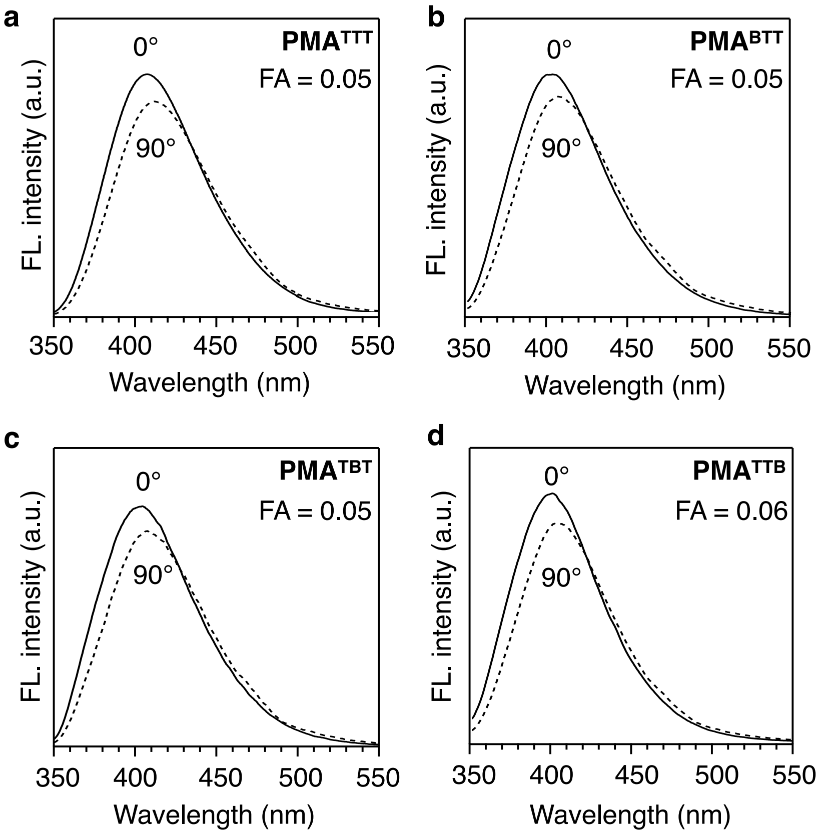

Supplementary Figure 30. Polarized emission spectra of (**a**) **PMA**$^{\mathbf{TTT}}$, (**b**) **PMA**$^{\mathbf{BTT}}$, (**c**) **PMA**$^{\mathbf{TBT}}$, and (**d**) **PMA**$^{\mathbf{TTB}}$ in $CHCl_3$ (0.1 mg/mL). The azimuthal angle is defined as 0° (solid

line) and 90° (broken line) when the excitation polarizer is parallel and perpendicular to the emission polarizer, respectively. Fluorescence anisotropy (FA) was computed by the formula FA = $(I_{0°} - I_{90°})/(I_{0°} + I_{90°})$, where $I_{0°}$ and $I_{90°}$ are the fluorescence intensities in the polarized emission spectra.

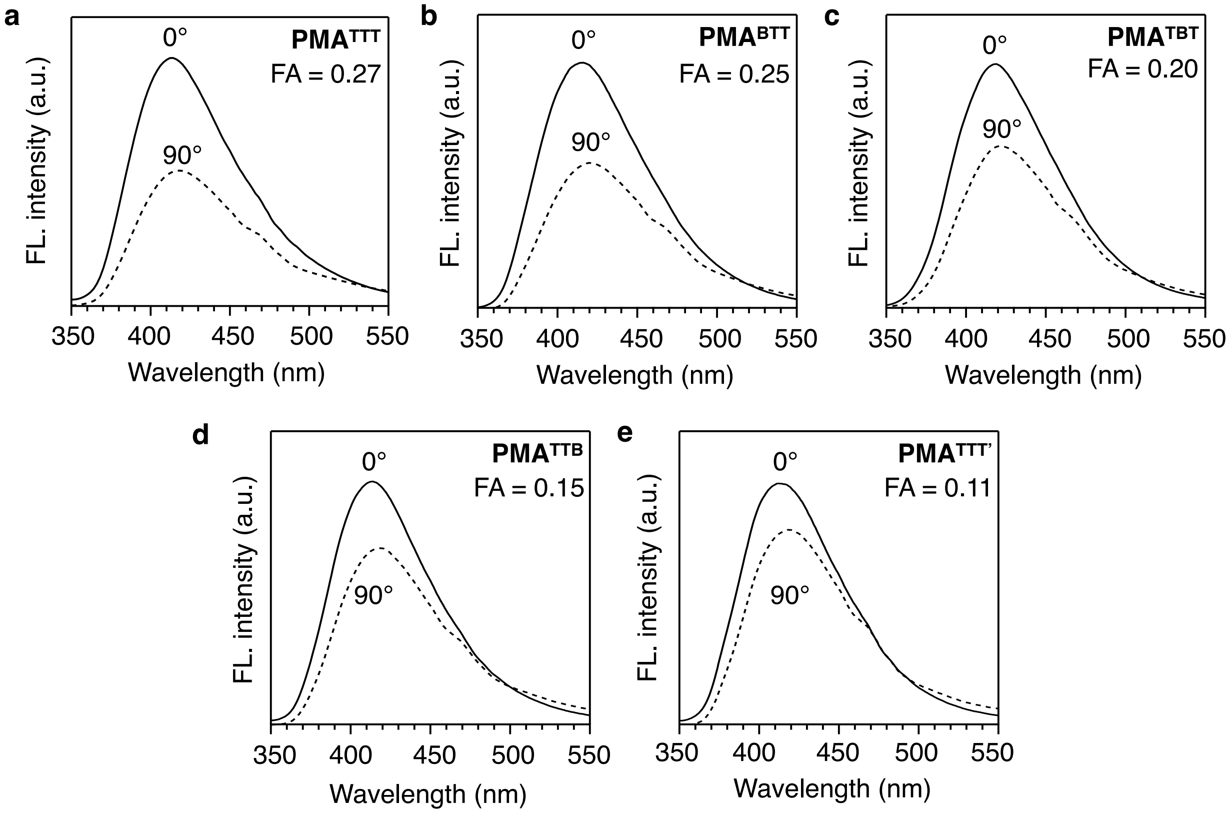

Supplementary Figure 31. Polarized emission spectra of hot-pressed films of (**a**) **PMA^{TTT}**, (**b**) **PMA^{BTT}**, (**c**) **PMA^{TBT}**, (**d**) **PMA^{TTB}** and (**e**) **PMA$^{TTT'}$** under the reflection mode. The polarizing direction of the exication light is parallel to the surface grooves on the Teflon sheets. The azimuthal angle is defined as 0° (solid line) and 90° (broken line) when the excitation polarizer is parallel and perpendicular to the emission polarizer, respectively. Fluorescence anisotropy (FA) was computed by the formula FA = $(I_{0°} - I_{90°})/(I_{0°} + I_{90°})$, where $I_{0°}$ and $I_{90°}$ are the fluorescence intensities in the polarized emission spectra.

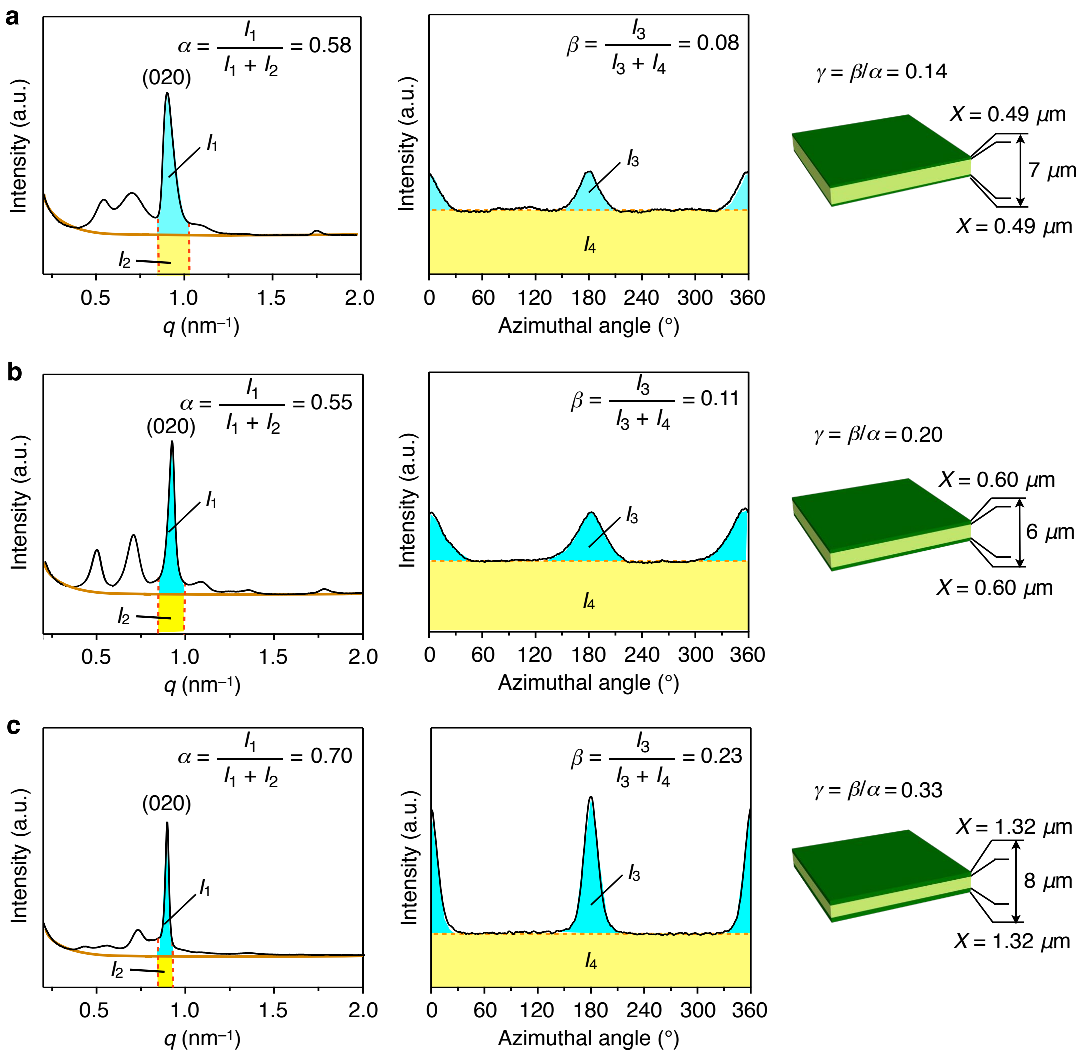

Supplementary Figure 32. Quantification of SAXS data to evaluate the contents of ordered domains (γ) of (**a**) **PMA**$^{\mathbf{BBB}}$, (**b**) **PMA**$^{\mathbf{BBA}}$ and (**c**) **PMA**$^{\mathbf{BAA}}$ in 7, 6 and 8 μm-thick hot-pressed films, respectively, prepared with two parallel Teflon sheets. In the dark and pale green-colored domains in the illustrations on the right hand side, polymer chains are oriented homeotropically and randomly, respectively.

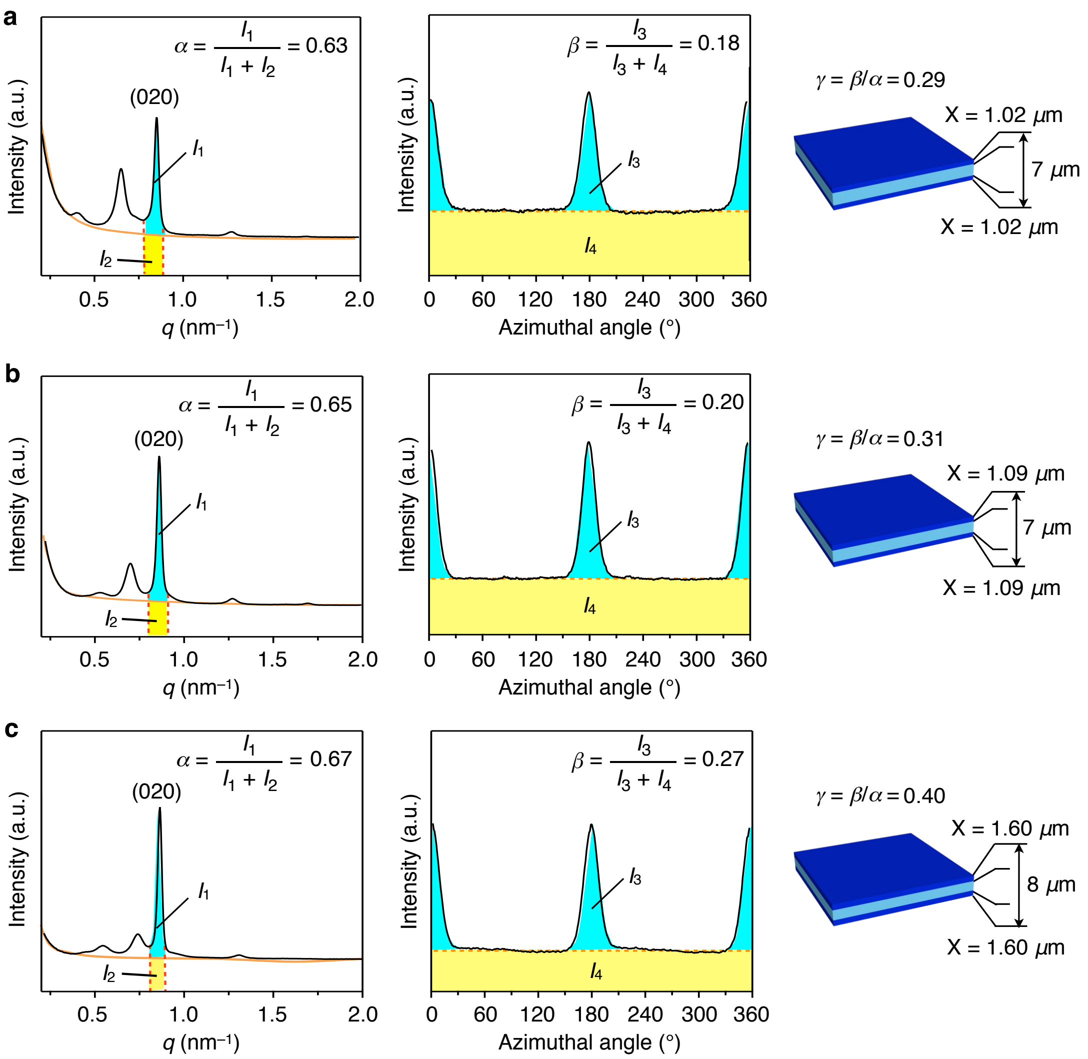

Supplementary Figure 33. Quantification of SAXS data to evaluate the contents of ordered domains (γ) of (**a**) **PMA**$^{\mathbf{TTT}}$, (**b**) **PMA**$^{\mathbf{TTA}}$ and (**c**) **PMA**$^{\mathbf{TAA}}$ in 7, 7 and 8-μm-thick hot-pressed films, respectively, prepared with parallel Teflon sheets. In the dark and pale blue-colored domains in the illustrations on the right hand side, polymer chains are oriented homeotropically and randomly, respectively.

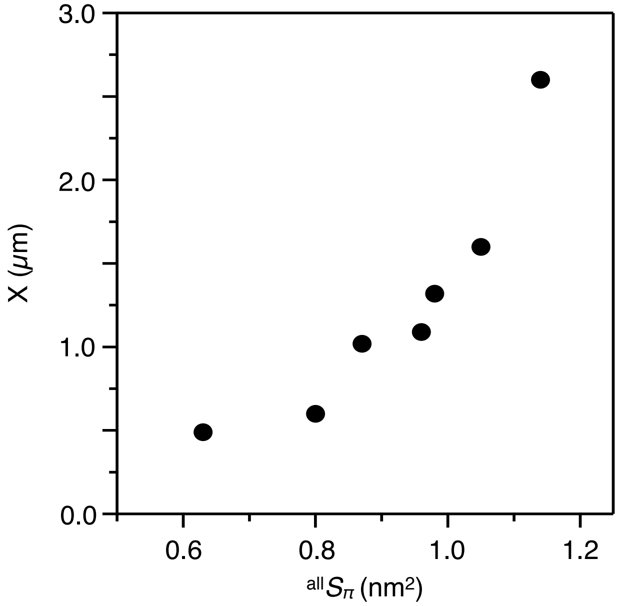

Supplementary Figure 34. Plot of total π-plane surface area of all mesogens (${}^{all}S_{\pi}$) in its individual side chains *versus* the thickness of the ordered area (X) in a hot-pressed film.

Supplementary Table 1. Polymerization of Macromonomers.

Polymer	Yield (%)	$M_n \times 10^{-4\,a}$	$M_w/M_n{}^a$	DP[b]
**PMA^{AAA}** S2	73	15.0	2.0	103
**PMA^{BBB}**	90	7.6	3.8	54
**PMA^{TTT}**	80	7.7	3.0	54
**PMA^{BBA}**	87	7.7	2.9	55
**PMA^{TTA}**	85	7.9	3.3	55
**PMA^{BAA}**	89	8.0	3.2	57
**PMA^{TAA}**	75	6.2	2.2	45
**PMA^{TTB}**	80	8.4	2.3	58
**PMA^{TBT}**	67	6.5	2.0	45
**PMA^{BTT}**	91	11.0	3.3	76
**PA^{BBB}**	47	3.8	1.4	28
**PPA^{BBB}**	70	6.2	2.0	43
**PMA$^{BBB'}$**	53	7.3	2.2	54
**PMA$^{TTT'}$**	80	12.0	2.7	85
**PMA^{BB}**	90	16.0	3.4	169
**PMA^{TT}**	88	7.6	3.1	71
**PMA^{B}**	92	5.1	3.8	88
**PMA^{T}**	95	7.0	4.0	113

[a]Determined by SEC (polystyrene standards) with $CHCl_3$ as an eluent. [b]Degree of polymerization (number-average repeating units of polymer chains).

Supplementary Table 2. Phase Transition Temperatures (T) and Enthalpy Changes (ΔH) of Polymers.

Polymer	Phase Sequence (Space Group)[a]	T (°C) heat / cool	ΔH (J g^{-1}) heat / cool
**PMA^{AAA}** [S2]	S ($P2_1/a$) / M ($P2_1/a$)	105 / 103	5.6 / 4.5
	M ($P2_1/a$) / Iso	124 / 120	16.6 / 17.3
**PMA^{BBB}**	S ($P2_1/a$) / M ($P2_1/a$)	no / 99	no / 1.9
	S ($P2_1/a$) / Iso	107 / no	20.4 / no
	M ($P2_1/a$) / Iso	no / 104	no / 18.8
**PMA^{TTT}**	S ($C2/m$) / M ($C2/m$)	95 / 92	6.2 / 5.8
	M ($C2/m$) / Iso	110 / 105	14.1 / 14.8
**PMA^{BBA}**	S ($P2_1/a$) / M ($P2_1/a$)	108 / 104	– / –
	M ($P2_1/a$) / Iso	112 / 107	18.2 / 20.2
**PMA^{TTA}**	S ($C2/m$) / M ($C2/m$)	97 / 94	5.2 / 4.8
	M ($C2/m$) / Iso	113 / 110	14.5 / 15.4
**PMA^{BAA}**	S ($P2_1/a$) / M ($P2_1/a$)	98 / 97	2.4 / 2.0
	M ($P2_1/a$) / Iso	118 / 115	16.0 / 16.4
**PMA^{TAA}**	S ($P2_1/a$) / M ($P2_1/a$)	103 / 100	5.6 / 5.0
	M ($P2_1/a$) / Iso	120 / 117	15.2 / 16.5
**PMA^{TTB}**	S ($C2/m$) / M ($C2/m$)	88 / 86	3.0 / 2.7
	M ($C2/m$) / Iso	105 / 101	11.9 / 12.7
**PMA^{TBT}**	S ($C2/m$) / M ($C2/m$)	89 / 88	1.8 / 1.7
	M (C2/m) / Iso	106 / 103	13.4 / 13.5
**PMA^{BTT}**	S ($C2/m$) / M ($C2/m$)	93 / 92	1.7 / 1.4
	M ($C2/m$) / Iso	108 / 103	14.4 / 14.7
**PA^{BBB}**	S ($P2/a$) / Iso	115 / 109	33.6 / 33.8
**PPA^{BBB}**	S ($C2/m$) / M ($C2/m$)	108 / 102	– / –
	M ($C2/m$) / Iso	113 / 107	19.6 / 19.0
**PMA$^{\mathrm{BBB'}}$**	S ($P6mm$) / Iso	107 / 101	24.1 / 24.6
**PMA$^{\mathrm{TTT'}}$**	S ($P6mm$) / M ($P6mm$)	98 / 97	4.7 / 3.9
	M ($P6mm$) / Iso	113 / 108	17.0 / 17.4
**PMA^{BB}**	S (–)[b] / Iso	79 / 75	9.0 / 9.4
**PMA^{TT}**	S (–)[b] / Iso	84 / 81	8.9 / 9.6
**PMA^{B}**	–[c]	no / no	no / no
**PMA^{T}**	–[c]	no / no	no / no

[a]S: solid, M: mesophase, Iso: isotropic melt, $P2_1/a$, $C2/m$, $P2/a$: rectangular lattice, $P6mm$: hexagonal lattice. [b]Not determined from SAXS pattern. [c]No phase transition.

Supplementary Table 3. SAXS Data of a Bulk Sample of **PMA[BBB]**.

Temp. (°C)	q (nm^{-1})	$d_{obs.}$ (nm)	$d_{calc.}$ (nm)	*hkl*	Temp. (°C)	q (nm^{-1})	$d_{obs.}$ (nm)	$d_{calc.}$ (nm)	*hkl*
25	0.543	11.58	11.55	110	100	0.548	11.45	11.31	110
($P2_1/a$)[a]	0.765	8.21	8.14	210	($P2_1/a$)[a]	0.770	8.15	8.16	210
	0.923	6.81	7.09	020		0.926	6.78	6.79	020
	1.120	5.61	6.00	310		1.129	5.56	5.65	310
	1.241	5.06	4.97	400		1.245	5.04	5.01	400
	1.587	3.96	3.85	330		1.620	3.88	3.77	330
	1.762	3.57	3.47	520		1.770	3.55	3.50	520
	2.128	2.95	3.00	620		1.853	3.39	3.39	040
	2.658	2.36	2.36	060		2.122	2.96	3.04	620
						2.664	2.36	2.36	060

[a]$P2_1/a$ rectangular lattice parameters a = 19.9 and b = 14.2 nm at 25 °C and a = 20.4 and b = 13.6 nm at 100 °C.

Supplementary Table 4. SAXS Data of a Bulk Sample of **PMA[BAA]**.

Temp. (°C)	q (nm^{-1})	$d_{obs.}$ (nm)	$d_{calc.}$ (nm)	*hkl*	Temp. (°C)	q (nm^{-1})	$d_{obs.}$ (nm)	$d_{calc.}$ (nm)	*hkl*
25	0.549	11.44	11.83	110	100	0.540	11.65	11.72	110
($P2_1/a$)[a]	0.726	8.65	8.52	210	($P2_1/a$)[a]	0.723	8.68	8.73	210
	0.887	7.08	7.12	020		0.887	7.08	7.09	020
	1.334	4.71	4.63	130		1.342	4.68	4.62	130
	1.648	3.81	3.94	330		1.659	3.79	3.91	330
	1.770	3.55	3.56	040		1.778	3.53	3.54	040
	2.481	2.53	2.51	450		2.174	2.89	2.81	150
	2.653	2.37	2.37	060		2.525	2.49	2.53	450
						2.667	2.35	2.36	060

[a]$P2_1/a$ rectangular lattice parameters a = 21.2 and b = 14.3 nm at 25 °C and a = 22.2 and b = 14.2 nm at 100 °C.

Supplementary Table 5. SAXS Data of a Bulk Sample of **PMA^{BBA}**.

Temp. (°C)	q (nm^{-1})	$d_{obs.}$ (nm)	$d_{calc.}$ (nm)	hkl	Temp. (°C)	q (nm^{-1})	$d_{obs.}$ (nm)	$d_{calc.}$ (nm)	hkl
25	0.520	12.09	11.96	110	105	0.503	12.01	11.84	110
($P2_1/a$)[a]	0.695	9.04	8.78	210	($P2_1/a$)[a]	0.712	8.82	8.84	210
	0.899	6.99	7.07	020		0.919	6.84	6.90	020
	1.083	5.80	5.60	400		1.091	5.75	5.75	400
	1.231	5.10	5.13	320		1.251	5.02	5.13	320
	1.350	4.65	4.61	130		1.390	4.52	4.51	130
	1.760	3.57	3.61	430		1.789	3.51	3.59	430
	1.814	3.46	3.53	040		1.828	3.44	3.45	040
	1.978	3.17	3.19	340		1.992	3.15	3.15	340
	2.650	2.37	2.36	060		2.134	2.94	2.96	440
						2.250	2.79	2.74	150
						2.692	2.33	2.30	060

[a] $P2_1/a$ rectangular lattice parameters a = 22.4 and b = 14.1 nm at 25 °C and a = 23.0 and b = 13.8 nm at 105 °C.

Supplementary Table 6. SAXS Data of a Bulk Sample of **PMA^{TTT}**.

Temp. (°C)	q (nm^{-1})	$d_{obs.}$ (nm)	$d_{calc.}$ (nm)	hkl	Temp. (°C)	q (nm^{-1})	$d_{obs.}$ (nm)	$d_{calc.}$ (nm)	hkl
25	0.657	9.57	9.58	110	100	0.650	9.66	9.65	110
($C2/m$)[a]	0.849	7.40	7.46	020	($C2/m$)[a]	0.855	7.35	7.36	020
	1.281	4.90	4.79	220		1.292	4.86	4.82	220
	1.683	3.73	3.73	040		1.705	3.68	3.68	040
	1.976	3.18	3.19	330		2.011	3.12	3.21	330
						2.547	2.47	2.47	060

[a] $C2/m$ rectangular lattice parameters a = 12.5 and b = 14.9 nm at 25 °C and a = 12.8 and b = 14.7 nm at 100 °C.

Supplementary Table 7. SAXS Data of a Bulk Sample of **PMA^{TAA}**.

Temp. (°C)	q (nm^{-1})	$d_{obs.}$ (nm)	$d_{calc.}$ (nm)	hkl	Temp. (°C)	q (nm^{-1})	$d_{obs.}$ (nm)	$d_{calc.}$ (nm)	hkl
25	0.519	12.10	12.46	110	110	0.522	12.02	12.07	110
($P2_1/a$)[a]	0.723	8.69	8.84	210	($P2_1/a$)[a]	0.720	8.72	8.75	210
	0.848	7.41	7.61	020		0.857	7.33	7.33	020
	1.295	4.85	4.94	130		1.309	4.80	4.77	130
	1.548	4.06	4.15	330		1.587	3.96	4.06	330
	1.687	3.73	3.80	040		1.714	3.66	3.67	040
	2.064	3.04	3.01	150		2.086	3.01	2.95	150
	2.353	2.67	2.65	450		2.431	2.58	2.58	450
						2.575	2.44	2.44	060

[a] $P2_1/a$ rectangular lattice parameters a = 21.7 and b = 15.2 nm at 25 °C and a = 21.8 and b = 14.7 nm at 110 °C.

Supplementary Table 8. SAXS Data of a Bulk Sample of $\mathbf{PMA^{TTA}}$.

Temp. (°C)	q (nm^{-1})	$d_{obs.}$ (nm)	$d_{calc.}$ (nm)	*hkl*	Temp. (°C)	q (nm^{-1})	$d_{obs.}$ (nm)	$d_{calc.}$ (nm)	*hkl*
25	0.682	9.22	9.23	110	105	0.679	9.25	9.23	110
(*C*2/*m*)[a]	0.849	7.40	7.44	020	(*C*2/*m*)[a]	0.855	7.35	7.32	020
	1.326	4.74	4.61	220		1.312	4.63	4.61	220
	1.691	3.72	3.72	040		1.713	3.67	3.66	040
	2.051	3.06	3.08	330		2.087	3.01	3.07	330
						2.569	2.44	2.44	060

[a]*C*2/*m* rectangular lattice parameters a = 11.8 and b = 14.9 nm at 25 °C and a = 11.9 and b = 14.7 nm at 105 °C.

Supplementary Table 9. SAXS Data of a Bulk Sample of $\mathbf{PMA^{TTB}}$.

Temp. (°C)	q (nm^{-1})	$d_{obs.}$ (nm)	$d_{calc.}$ (nm)	*hkl*	Temp. (°C)	q (nm^{-1})	$d_{obs.}$ (nm)	$d_{calc.}$ (nm)	*hkl*
25	0.659	9.53	9.63	110	100	0.662	9.49	9.53	110
(*C*2/*m*)[a]	0.864	7.27	7.29	020	(*C*2/*m*)[a]	0.868	7.24	7.25	020
	1.291	4.86	4.82	220		1.313	4.78	4.77	220
	1.721	3.65	3.64	040		1.730	3.63	3.63	040
	2.089	3.01	3.21	330		2.132	2.95	3.18	330
						2.528	2.48	2.42	060

[a]*C*2/*m* rectangular lattice parameters a = 12.8 and b = 14.6 nm at 25 °C and a = 12.6 and b = 14.5 nm at 100 °C.

Supplementary Table 10. SAXS Data of a Bulk Sample of $\mathbf{PMA^{TBT}}$.

Temp. (°C)	q (nm^{-1})	$d_{obs.}$ (nm)	$d_{calc.}$ (nm)	*hkl*	Temp. (°C)	q (nm^{-1})	$d_{obs.}$ (nm)	$d_{calc.}$ (nm)	*hkl*
25	0.653	9.62	9.63	110	100	0.646	9.72	9.62	110
(*C*2/*m*)[a]	0.852	7.35	7.38	020	(*C*2/*m*)[a]	0.858	7.32	7.31	020
	1.304	4.82	4.82	220		1.319	4.76	4.81	220
	1.699	3.70	3.69	040		1.721	3.65	3.66	040

[a]*C*2/*m* rectangular lattice parameters a = 12.7 and b = 14.8 nm at 25 °C and a = 12.8 and b = 14.6 nm at 100 °C.

Supplementary Table 11. SAXS Data of a Bulk Sample of **PMA**$^{\mathbf{BTT}}$.

Temp. (°C)	q (nm^{-1})	$d_{obs.}$ (nm)	$d_{calc.}$ (nm)	*hkl*	Temp. (°C)	q (nm^{-1})	$d_{obs.}$ (nm)	$d_{calc.}$ (nm)	*hkl*
25	0.649	9.68	9.59	110	100	0.649	9.68	9.54	110
(*C*2/*m*)[a]	0.874	7.19	7.22	020	(*C*2/*m*)[a]	0.874	7.19	7.20	020
	1.322	4.75	4.80	220		1.335	4.70	4.77	220
	1.737	3.62	3.61	040		1.746	3.60	3.60	040
	1.963	3.20	3.20	330		1.960	3.20	3.18	330
						2.622	2.40	2.40	060

[a]*C*2/*m* rectangular lattice parameters a = 12.9 and b = 14.4 nm at 25 °C and a = 12.7 and b = 14.4 nm at 100 °C.

Supplementary Table 12. SAXS Data of a Bulk Sample of **PMA**$^{\mathbf{BBB'}}$.

Temp. (°C)	q (nm^{-1})	$d_{obs.}$ (nm)	$d_{calc.}$ (nm)	*hkl*	Temp. (°C)	q (nm^{-1})	$d_{obs.}$ (nm)	$d_{calc.}$ (nm)	*hkl*
25	0.581	10.81	11.05	100	100	0.571	11.00	11.21	100
(*P*6*mm*)[a]	0.968	6.49	6.38	110	(*P*6*mm*)[a]	0.954	6.58	6.47	110
	1.455	4.32	4.20	210		1.460	4.30	4.26	210
	1.751	3.59	3.68	300		1.707	3.68	3.74	300

[a]*P*6*mm* hexagonal lattice parameter a = 12.7 nm at 25 °C and a = 12.9 nm at 100 °C.

Supplementary Table 13. SAXS Data of a Bulk Sample of **PMA**$^{\mathbf{TTT'}}$.

Temp. (°C)	q (nm^{-1})	$d_{obs.}$ (nm)	$d_{calc.}$ (nm)	*hkl*	Temp. (°C)	q (nm^{-1})	$d_{obs.}$ (nm)	$d_{calc.}$ (nm)	*hkl*
25	0.540	11.63	11.78	100	105	0.537	11.69	11.80	100
(*P*6*mm*)[a]	0.918	6.84	6.80	110	(*P*6*mm*)[a]	0.913	6.88	6.81	110
	1.392	4.51	4.45	210		1.371	4.58	4.46	210
	1.622	3.88	3.93	300		1.622	3.88	3.93	300

[a]*P*6*mm* hexagonal lattice parameter a = 13.6 nm at 25 °C and a = 13.6 nm at 105 °C.

Supplementary Table 14. SAXS Data of a Bulk Sample of **PA**[BBB].

Temp. (°C)	q (nm^{-1})	$d_{obs.}$ (nm)	$d_{calc.}$ (nm)	*hkl*	Temp. (°C)	q (nm^{-1})	$d_{obs.}$ (nm)	$d_{calc.}$ (nm)	*hkl*
25	0.645	9.74	9.38	110	100	0.636	9.87	9.91	110
(*P*2/*a*)[a]	0.947	6.64	6.93	200	(*P*2/*a*)[b]	0.942	6.67	6.89	200
	1.357	4.63	4.63	300		1.261	4.98	4.96	220
	1.895	3.32	3.34	410		1.352	4.65	4.59	300
	2.253	2.79	2.78	500		1.884	3.34	3.35	410
						2.242	2.80	2.75	500
						2.630	2.39	2.38	530

[a]*P*2/*a* rectangular lattice parameters a = 13.9 and b = 12.4 nm at 25 °C and a = 13.8 and b = 14.3 nm at 100 °C.

Supplementary Table 15. SAXS Data of a Bulk Sample of **PPA**[BBB].

Temp. (°C)	q (nm^{-1})	$d_{obs.}$ (nm)	$d_{calc.}$ (nm)	*hkl*	Temp. (°C)	q (nm^{-1})	$d_{obs.}$ (nm)	$d_{calc.}$ (nm)	*hkl*
25	0.706	8.90	9.32	110	100	0.702	8.95	8.68	110
(*C*2/*m*)[a]	0.889	7.07	7.07	020	(*C*2/*m*)[b]	0.911	6.90	6.91	020
	1.329	4.73	4.71	220		1.384	4.54	4.34	220
	1.776	3.54	3.54	040		1.815	3.46	3.45	040
						2.191	2.87	2.89	330
						2.730	2.30	2.30	060

[a]*C*2/*m* rectangular lattice parameters a = 12.6 and b = 14.2 nm at 25 °C and a = 11.2 and b = 13.8 nm at 100 °C.

Supplementary Figure 16. Polarized IR Data of Hot-Pressed Polymer Films.

	$[A_{//}/A_{\perp}]^{\#}$			
	$\nu_{(C=O)}$ 1714 cm^{-1}	$\nu_{(C_{Ar}–C_{Ar})}$ 1604 cm^{-1}	$\nu_{(C–O)}$ 1277 cm^{-1}	$\nu_{(C_{Ar}–O)}$ 1245 cm^{-1}
**PMA^{AAA}** S2	0.65	2.29	2.31	2.69
**PMA^{BBB}**	0.74	1.25	1.25	1.24
**PMA^{TTT}**	0.72	1.59	1.54	1.54
**PMA^{BBA}**	0.71	1.33	1.35	1.39
**PMA^{TTA}**	0.71	1.57	1.55	1.57
**PMA^{BAA}**	0.70	1.58	1.52	1.59
**PMA^{TAA}**	0.69	1.62	1.59	1.59
**PMA^{TTB}**	0.77	1.36	1.35	1.41
**PMA^{TBT}**	0.77	1.35	1.33	1.38
**PMA^{BTT}**	0.80	1.31	1.28	1.33
**PMA$^{BBB'}$**	1.02	1.04	0.95	1.01
**PMA$^{TTT'}$**	0.99	0.97	0.98	0.98

$^{\#}$Dichroic ratio $[A_{//}/A_{\perp}]$ of designated absorption bands, in which the incident infrared radiation is polarized parallel (//) and perpendicular (⊥) to the stretched direction of a PTFE sheet.

Supplementary Disscussion

Evaluation of the order parameters of hot-pressed films were performed using a method similar to that reported by Daxer and Fratzl[9]. 2D-SAXS images (Supplementary fig. 16) were integrated along the Debye–Scherrer ring to obtain scattering intensity profiles (left insets in Supplementary figs. 32 and 33) as a function of scattering vector q ranging from 0.2 to 2 nm^{-1}. Symbol α represents the contribution of both oriented and non-oriented polymers to the total scattering corresponding to the scattering from the (020) plane, which is obtained by the following Supplementary Equation 1:

$$\alpha = I_1/(I_1 + I_2) \qquad \text{----------------------------------- (1)}$$

where I_1 and I_2 denote integrals of the blue and yellow parts in the left insets of supplementary figs. 29 and 30, respectively. Then, the scattering intensities of the (020) plane were plotted against the azimuthal angle θ (right insets in Supplementary figs. 32 and 33). Symbol β represents the contribution of oriented polymers alone to the total scattering, which is obtained by the following Supplementary Equation 2:

$$\beta = I_3/(I_3 + I_4) \qquad \text{----------------------------------- (2)}$$

where I_3 and I_4 denote integrals of the blue and yellow parts in the right insets of supplementary figs. 29 and 30, respectively. The degree of lattice orientation is defined as Supplementary Equation 3:

$$\gamma = \beta/\alpha \qquad \text{----------------------------------- (3)}$$

If all polymer molecules are randomly oriented in the film, symbol γ should be equal to zero. If all polymer molecules align undirectionally, symbol γ should be equal to one. Since the lattice orientation most likely develops equally on both sides of the film, toward the inner side from the interface with the Teflon sheets, the hot-pressed film adopts a bimorph configuration consisting of a disordered layer (Y) sandwiched by two ordered layers (X). Based on this configurational feature, the thicknesses of Y and X can be estimated from the γ value and total thickness of the hot-pressed film (d), provided that the thicknesses of ordered domians on both sides of the film are identical to one another.

$$\mathrm{X} = \gamma d/2 \qquad \text{----------------------------------- (4)}$$

$$\mathrm{Y} = (1-\gamma)d \qquad \text{----------------------------------- (5)}$$

Supplementary Methods

Materials. Unless otherwise noted, reagents were used as received from Sigma-Aldrich [2-hexyl-1-decanol, ethyl 4-hydroxy-4'-biphenylcarboxylate], Tokyo Chemical Industry [10-bromo-1-decanol, *tert*-butyldimethylchlorosilane (TBDMSCl), tetrabutylammonium fluoride (TBAF, 1 M in tetrahydrofuran (THF)), trimethylsilylacetylene and tetrakis(triphenylphosphine)palladium], and Wako Pure Chemical Industries [*N,N'*-diisopropylcarbodiimide (DIPC), *p*-toluenesulfonic acid monohydrate, 4-dimethylaminopyridine (DMAP), triethylamine, 4,4′-biphenol, 2,2'-azobisisobutyronitrile (AIBN), 4-iodophenol, methacryloyl chloride, acryloyl chloride, imidazole, CuI, Cs_2CO_3, Na_2CO_3 and KOH]. 4-(Dimethylamino)pyridinuim 4-toluenesulfonate (DPTS)[1], 10-Bromodecyl *tert*-butyldimethylsilyl ether[2], 1-bromo-2-hexyldecane[3], 4-ethynylbenzoic acid[4], Rh(nbd)BPh_4[5], compounds **12**[2] and **18**[2] were prepared according to the procedures analogous to those reported previously. CH_2Cl_2 was dried over CaH_2 and freshly distilled prior to use. THF was refluxed over a mixture of Na and benzophenone and freshly distilled before use. Other dehydrated solvents were purchased from Wako Pure Chemical Industries or Kanto Chemicals. Uniaxially stretched polytetrafluoroethylene (PTFE, Teflon™) sheets (300 mm × 10 m × 0.1 mm) were purchased from Flon Industry and cut into appropriate dimensions before use.

General. Column chromatography was carried out with Wakogel silica C-300 (particle size: 45–75 μm). Recycling preparative size-exclusion chromatography (SEC) was performed using JAIGEL 2H and 2.5H columns on a JAI model LC-9201 recycling HPLC system equipped with a JASCO model MD-2010 Plus variable-wavelength UV-vis detector using $CHCl_3$/MeOH (100/1 v/v) as an eluent. Analytical SEC was performed at 40 °C on a TOSOH model HLC-8220 GPC system equipped with a refractive index (RI) detector, using $CHCl_3$ as an eluent at a flow rate of 0.35 mL min^{-1} on linearly connected two polystyrene gel columns (TSKgel SuperHM-M, TOSOH). The molecular weight calibration curve was obtained by using standard polystyrenes (Shodex STANDARD SM–105, Showa Denko). ^{1}H and ^{13}C NMR spectra were recorded on a JEOL model JNM-ECA500 spectrometer operating at 500 and 125 MHz, respectively, where chemical shifts (δ in ppm) were determined with respect to tetramethylsilane as an internal reference. Matrix-assisted laser desorption ionization time-of-flight (MALDI-TOF) mass spectrometry was performed on an Applied Biosystems model MDS SCIEX 4800 Plus MALDI TOF/TOF™ Analyzer using dithranol as a matrix. Infrared (IR) spectra were recorded at 25 °C on a JASCO model FT/IR-4100 Fourier transform infrared spectrometer with an attenuated total reflection (ATR) equipment (ATR PRO450-S). Polarized IR spectra were recorded at 25 °C on a JASCO model FT/IR-4100 Fourier transform infrared spectrometer equipped with an Irtron IRT-5000 microscope unit. Polarized fluorescence spectra were recorded at 25 °C on a JASCO model FP-6500 spectrofluorometer equppied with UV/vis and vis polarizers. Differential scanning calorimetry (DSC) was performed on a Mettler–Toledo model DSC 1 differential scanning

calorimeter, where temperature and enthalpy were calibrated with In (430 K, 3.3 J mol^{-1}) and Zn (692.7 K, 12 J mol^{-1}) standard samples using sealed Al pans. Cooling and heating profiles were recorded and analyzed using the Mettler–Toledo $STAR^{e}$ software system. Polarized optical microscopy (POM) was performed on a Nikon model Eclipse LV100POL optical polarizing microscope equipped with a Mettler–Toledo model FP90 controller. Hot press was performed on an Imoto Machinery model IMC-180C compact press. Film thicknesses were measured using a Mitutoyo model MDQ-30M micrometer. Small-angle X-ray scattering (SAXS) and wide-angle X-ray diffraction (WAXD) measurements were carried out at BL45XU in SPring-8 (Hyogo, Japan) with an imaging plate area detector, a R-AXIS IV++ (Rigaku). Scattering vector, $q = 4\pi\sin\theta/\lambda$, and the position of an incident X-ray on the detector were calibrated using several orders of layer reflections from silver behenate (d = 58.380 Å), where 2θ and λ are scattering angle and wavelength of X-ray (1.0 Å), respectively. The sample-to-detector distances for SAXS and WAXD measurements were 2.0 and 0.4 m, respectively. The scattering/diffraction images recorded were integrated along the Debye-Scherrer ring, affording one-dimensional intensity data using the FIT2D software[6]. The cell parameters were refined using the CellCalc ver. 2.10 software[7]. Powdery samples were placed into a 1.0 mm-ϕ glass capillary in a temperature-controlled heating block and once heated to isotropic melt. The resultant samples were then exposed to an X-ray beam for 10 (WAXD) or 100 (SAXS) seconds at given temperatures. Film samples were clipped with tweezers or sandwiched by sapphire glasses and exposed at 25 °C to an X-ray beam for 300 seconds. Dipole moments of three mesogens **A**, **B** and **T** were calculated with the density functional theory (DFT) method at the B3LYP/6-31G(d) level using a Gaussian 03 package[8].

Synthesis

Synthesis of 5, 8 and 11

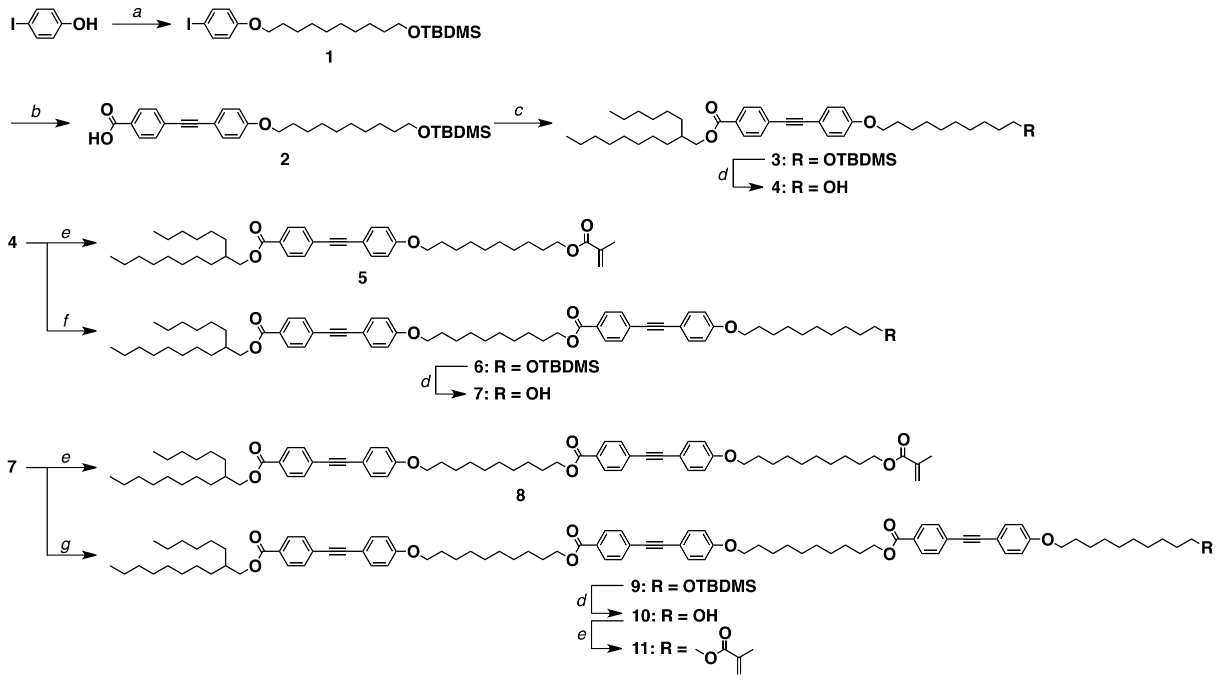

Reagents and conditions: (a) 10-bromodecyl *tert*-butyldimethylsilyl ether, Cs_2CO_3, DMF, 70 °C; (b) 4-ethynylbenzoic acid, CuI, triethylamine, tetrakis(triphenylphosphine)palladium, THF, reflux; (c) 2-hexyl-1-decanol, DPTS, DIPC, CH_2Cl_2, 25 °C; (d) TBAF, THF, 25 °C; (e) methacryloyl chloride, triethylamine, CH_2Cl_2, 25 °C; (f) **2**, DPTS, DIPC, CH_2Cl_2, 25 °C; (g) **2**, DPTS, DIPC, CH_2Cl_2, 40 °C.

Compound 1. 10-Bromodecyl *tert*-butyldimethylsilyl ether (6.9 g, 19.7 mmol) and Cs_2CO_3 (7.3 g, 22.5 mmol) were successively added to a DMF solution (45 mL) of 4-iodophenol (4.1 g, 18.6 mmol), and the mixture was stirred at 70 °C for 18 h under N_2. The reaction mixture was poured into a saturated aqueous solution of NH_4Cl and extracted with AcOEt. The combined organic extract was washed successively with water and brine, and an organic phase separated was dried over anhydrous $MgSO_4$ and then evaporated to dryness under a reduced pressure. The residue was subjected to column chromatography (SiO_2, hexane/AcOEt 10/1 v/v) to allow isolation of **1** as colorless oil (7.5 g, 15.3 mmol) in 82% yield. ^{1}H NMR (500 MHz, $CDCl_3$): δ (ppm) 7.51 (d, J = 9.2 Hz, 2H), 6.65 (d, J = 9.2 Hz, 2H), 3.88 (t, J = 6.6 Hz, 2H), 3.58 (t, J = 6.6 Hz, 2H), 1.74 (m, 2H), 1.49 (m, 2H), 1.41 (m, 2H), 1.27 (br, 10H), 0.88 (s, 9H), 0.03 (s, 6H). ^{13}C NMR (125 MHz, $CDCl_3$): δ (ppm) 159.16, 138.28, 117.08, 82.52, 68.25, 63.46, 33.03, 29.69, 29.63, 29.55, 29.49, 29.28, 26.14, 25.94, 18.53, –5.10. MALDI-TOF mass: calcd. for $C_{22}H_{39}INaO_2Si$ $[M + Na]^+$: m/z = 513.17; found: 513.19.

Compound 2. To a THF solution (30 mL) of **1** (5.2 g, 10.7 mmol) were successively added 4-ethynylbenzoic acid (1.9 g, 12.8 mmol), CuI (0.051 g, 0.3 mmol), triethylamine (30 mL), and tetrakis(triphenylphosphine)palladium (0.616 g, 0.5 mmol), and the mixture was purged with N_2 for 20 min and then refluxed for 18 h under N_2. The reaction mixture was poured into a saturated aqueous solution of NH_4Cl and extracted successively with AcOEt, CH_2Cl_2 and ether. The combined organic extract was evaporated to dryness under a reduced pressure, and the residue was recrystallized from MeOH, affording **2** as pale yellow solid (3.9 g, 7.6 mmol) in 71% yield. ^{1}H NMR (500 MHz, DMSO-d_6, 60 °C): δ (ppm) 7.90 (d, J = 8.1 Hz, 2H), 7.51–7.47 (m, 4H), 6.96 (d, J = 8.6 Hz, 2H), 4.01 (t, J = 6.6 Hz, 2H), 3.57 (t, J = 6.3 Hz, 2H), 1.72 (m, 2H), 1.43 (m, 4H), 1.32–1.28 (br, 10H), 0.87 (s, 9H), 0.02 (s. 6H). ^{13}C NMR (125 MHz, DMSO-d_6, 60 °C): δ (ppm) 166.38, 159.19, 132.87, 130.94, 130.01, 129.23, 126.91, 114.77, 113.41, 92.13, 87.12, 67.56, 62.23, 31.99, 28.63, 28.56, 28.40, 28.30, 25.55, 25.15, 24.97, 17.64, −5.56. MALDI-TOF mass: calcd. for $C_{31}H_{43}Na_2O_4Si$ $[M - H + 2Na]^+$: m/z = 553.27; found: 553.37.

Compound 3. To a CH_2Cl_2 solution (20 mL) of **2** (1.50 g, 2.95 mmol) were successively added DPTS (0.17 g, 0.59 mmol) and DIPC (0.48 g, 3.81 mmol), and the mixture was stirred at 25 °C under Ar until all the reagents were dissolved. 2-Hexyl-1-decanol (0.75 g, 3.09 mmol) was added to the resulting solution, and the mixture was stirred at 25 °C for 12 h under Ar. The reaction mixture was poured into water and extracted with $CHCl_3$. The combined organic extract was washed successively with water and brine, and an organic phase separated was dried over anhydrous $MgSO_4$ and then evaporated to dryness under a reduced pressure. The residue was subjected to column chromatography (SiO_2, $CHCl_3$) to allow isolation of **3** as colorless oil (2.10 g, 2.86 mmol) in 97% yield. ^{1}H NMR (500 MHz, $CDCl_3$): δ (ppm) 8.00 (d, J = 8.0 Hz, 2H), 7.55 (d, J = 8.0 Hz, 2H), 7.47 (d, J = 8.6 Hz, 2H), 6.88 (d, J = 8.6 Hz, 2H), 4.23 (d, J = 5.8 Hz, 2H), 3.97 (t, J = 6.3 Hz, 2H), 3.60 (t, J = 6.3 Hz, 2H), 1.82–1.76 (m, 3H), 1.54–1.22 (m, 38H), 0.90–0.86 (m, 15H), 0.05 (s, 6H). ^{13}C NMR (125 MHz, $CDCl_3$): δ (ppm) 166.31, 159.63, 133.24, 131.28, 129.53, 129.44, 128.34, 114.63, 114.55, 92.62, 87.50, 68.11, 67.94, 63.08, 37.47, 32.81, 31.92, 31.83, 31.47, 29.96, 29.63, 29.57, 29.53, 29.48, 29.42, 29.36, 29.32, 29.19, 26.78, 26.76, 26.01, 25.74, 25.66, 22.69, 22.66, 18.39, 14.11, −5.23. MALDI-TOF mass: calcd. for $C_{47}H_{77}O_4Si$ $[M + H]^+$: m/z = 733.56; found: 733.56.

Compound 4. TBAF (1.0 M in THF, 6.0 mL, 6.0 mmol) was added to a THF solution (20 mL) of **3** (2.0 g, 2.84 mmol), and the mixture was stirred at 25 °C for 12 h under Ar. The reaction mixture was evaporated to dryness under a reduced pressure, and a $CHCl_3$ solution of the residue was washed with a saturated aqueous solution of NH_4Cl. An organic phase separated was dried over anhydrous $MgSO_4$ and evaporated to dryness under a reduced pressure. The residue was subjected to column chromatography (SiO_2, $CHCl_3$) to allow isolation of **4** as colorless oil (1.77 g,

2.58 mmol) in 91% yield. ^{1}H NMR (500 MHz, $CDCl_3$): δ (ppm) 8.00 (d, J = 8.0 Hz, 2H), 7.55 (d, J = 8.0 Hz, 2H), 7.47 (d, J = 8.6 Hz, 2H), 6.88 (d, J = 8.6 Hz, 2H), 4.23 (d, J = 5.8 Hz, 2H), 3.97 (t, J = 6.3 Hz, 2H), 3.64 (m, 2H), 1.82−1.76 (m, 3H), 1.60−1.54 (m, 2H), 1.47−1.19 (m, 37H), 0.89–0.86 (m, 6H). ^{13}C NMR (125 MHz, $CDCl_3$): δ (ppm) 166.22, 159.54, 133.15, 131.20, 129.53, 129.44, 128.34, 114.60, 114.55, 92.63, 87.51, 68.11, 67.94, 63.06, 37.47, 32.81, 31.92, 31.83, 31.47, 29.96, 29.63, 29.57, 29.53, 29.48, 29.41, 29.36, 29.32, 29.20, 26.78, 26.76, 26.01, 25.75, 25.66, 22.68, 22.66, 14.11. MALDI-TOF mass: calcd. for $C_{41}H_{63}O_4$ $[M + H]^+$: m/z = 619.47; found: 619.47.

Compound 5. To a CH_2Cl_2 solution (15 mL) of **4** (0.70 g, 1.13 mmol) were successively added methacryloyl chloride (0.24 g, 2.21 mmol) and triethylamine (0.21 g, 2.20 mmol), and the mixture was stirred at 25 °C for 10 h under Ar. The reaction mixture was poured into water and extracted with $CHCl_3$. The combined organic extract was washed successively with a saturated aqueous solution of $NaHCO_3$, water and brine, and an organic phase separated was dried over anhydrous $MgSO_4$ and then evaporated to dryness under a reduced pressure. The residue was subjected to column chromatography (SiO_2, $CHCl_3$/hexane 1/1 v/v) to allow isolation of **5** as colorless oil (0.63 g, 0.91 mmol) in 80% yield. ^{1}H NMR (500 MHz, $CDCl_3$): δ (ppm) 8.00 (d, J = 8.0 Hz, 2H), 7.55 (d, J = 8.0 Hz, 2H), 7.47 (d, J = 8.6 Hz, 2H), 6.87 (d, J = 8.6 Hz, 2H), 6.10 (s, 1H), 5.54 (t, J = 1.7 Hz, 1H), 4.23 (d, J = 5.8 Hz, 2H), 4.13 (t, J = 6.9 Hz, 2H), 3.97 (t, J = 6.3 Hz, 2H), 1.94 (br, 3H), 1.82−1.76 (m, 3H), 1.70−1.64 (m, 2H), 1.51−1.28 (m, 36H), 0.89−0.86 (m, 6H). ^{13}C NMR (125 MHz, $CDCl_3$): δ (ppm) 167.56, 166.28, 159.61, 136.58, 133.23, 131.27, 129.53, 129.44, 128.33, 125.13, 114.61, 114.54, 92.61, 87.50, 68.09, 67.92, 64.81, 37.46, 31.91, 31.82, 31.46, 29.96, 29.56, 29.45, 29.44, 29.34, 29.31, 29.23, 29.18, 28.62, 26.77, 26.76, 26.00, 25.97, 22.68, 22.65, 18.33, 14.12. MALDI-TOF mass: calcd. for $C_{45}H_{67}O_5$ $[M + H]^+$: m/z = 687.50; found: 687.45.

Compound 6. By a procedure similar to that for **3**, compound **6** was obtained in 75% yield (2.16 g, 1.95 mmol) from **2** (1.40 g, 2.75 mmol), **4** (1.61 g, 2.60 mmol), DPTS (0.15 g, 0.54 mmol) and DIPC (0.48 g, 3.81 mmol). ^{1}H NMR (500 MHz, $CDCl_3$): δ (ppm) 8.00 (d, J = 8.0 Hz, 2H), 8.00 (d, J = 8.0 Hz, 2H), 7.55 (d, J = 8.0 Hz, 4H), 7.46 (d, J = 8.6 Hz, 4H), 6.87 (d, J = 8.6 Hz, 2H), 6.87 (d, J = 8.6 Hz, 2H), 4.32 (t, J = 6.6 Hz, 2H), 4.22 (d, J = 5.8 Hz, 2H), 3.96 (t, J = 6.3 Hz, 4H), 3.60 (t, J = 6.6 Hz, 2H), 1.81−1.74 (m, 7H), 1.59−1.28 (m, 50H), 0.91−0.86 (m, 15H), 0.05 (s, 6H). ^{13}C NMR (125 MHz, $CDCl_3$): δ (ppm) 166.30, 166.23, 159.62, 133.23, 131.27, 129.52, 129.44, 128.36, 114.62, 114.53, 92.66, 92.63, 87.50, 68.10, 67.94, 65.27, 63.08, 37.46, 32.79, 31.91, 31.82, 31.46, 29.95, 29.62, 29.56, 29.52, 29.47, 29.45, 29.43, 29.40, 29.35, 29.23, 29.18, 26.77, 26.75, 26.00, 25.73, 25.66, 22.68, 22.66, 18.39, 14.11, −5.23. MALDI-TOF mass: calcd. for $C_{72}H_{105}O_7Si$ $[M + H]^+$: m/z = 1109.76; found: 1109.70.

Compound 7. By a procedure similar to that for **4**, compound **7** was obtained in 88% yield (1.65 g, 1.66 mmol) from **6** (2.10 g, 1.89 mmol) and TBAF (1.0 M in THF, 4.0 mL, 4.0 mmol). ^{1}H NMR (500 MHz, $CDCl_3$): δ (ppm) 8.00 (d, J = 8.0 Hz, 2H), 7.99 (d, J = 8.0 Hz, 2H), 7.55 (d, J = 8.0 Hz, 2H), 7.55 (d, J = 8.0 Hz, 2H), 7.46 (d, J = 8.6 Hz, 4H), 6.87 (d, J = 8.6 Hz, 2H), 6.87 (d, J = 8.6 Hz, 2H), 4.32 (t, J = 6.6 Hz, 2H), 4.23 (d, J = 5.8 Hz, 2H), 3.96 (t, J = 6.3 Hz, 4H), 3.64 (t, J = 6.6 Hz, 2H), 1.81–1.74 (m, 7H), 1.59–1.54 (m, 2H), 1.45–1.28 (m, 49H), 0.91–0.86 (m, 6H). ^{13}C NMR (125 MHz, $CDCl_3$): δ (ppm) 166.31, 166.22, 159.63, 133.22, 131.27, 129.51, 129.44, 128.37, 114.63, 114.53, 92.66, 92.64, 87.51, 68.11, 67.94, 65.27, 63.08, 37.47, 32.79, 31.92, 31.82, 31.46, 29.96, 29.63, 29.56, 29.51, 29.48, 29.45, 29.43, 29.40, 29.36, 29.23, 29.18, 26.77, 26.74, 26.00, 25.73, 25.66, 22.68, 22.66, 14.11. MALDI-TOF mass: calcd. for $C_{66}H_{91}O_7$ $[M + H]^+$: m/z = 995.68; found: 995.70.

Compound 8. By a procedure similar to that for **5**, compound **8** was obtained in 70% yield (0.46 g, 0.43 mmol) from **7** (0.61 g, 0.61 mmol), methacryloyl chloride (0.16 g, 1.21 mmol) and triethylamine (0.16 g, 1.20 mmol). ^{1}H NMR (500 MHz, $CDCl_3$): δ (ppm) 8.00 (d, J = 8.0 Hz, 2H), 7.99 (d, J = 8.0 Hz, 2H), 7.55 (d, J = 8.6 Hz, 4H), 7.47 (d x 2, J = 8.6 Hz, 4H), 6.87 (d, J = 8.6 Hz, 4H), 6.10 (m, 1H), 5.54 (m, 1H), 4.32 (t, J = 6.7 Hz, 2H), 4.23 (d, J = 5.7 Hz, 2H), 4.14 (t, J = 6.6 Hz, 2H), 3.97 (t, J = 6.3 Hz, 2H), 3.97 (t, J = 6.3 Hz, 2H), 1.94 (br, 3H), 1.84–1.76 (m, 7H), 1.70–1.64 (m, 2H), 1.46–1.28 (m, 48H), 0.89–0.86 (m, 6H). ^{13}C NMR (125 MHz, $CDCl_3$): δ (ppm) 167.56, 166.35, 159.61, 136.65, 133.23, 131.27, 129.50, 129.44, 128.35, 125.17, 114.62, 114.54, 92.60, 87.59, 68.10, 65.26, 64.82, 37.50, 31.91, 31.82, 31.46, 29.96, 29.56, 29.46, 29.43, 29.34, 29.31, 29.24, 29.18, 28.73, 28.62, 26.77, 26.01, 25.97, 22.68, 22.65, 18.33, 14.13, 14.11. MALDI-TOF mass: calcd. for $C_{70}H_{95}O_8$ $[M + H]^+$: m/z = 1063.70; found: 1063.68.

Compound 9. By a procedure similar to that for **3**, except that the reaction temperature was 40 °C, compound **9** was obtained in 81% yield (1.20 g, 0.81 mmol) from **2** (0.55 g, 1.10 mmol), **7** (1.0 g, 1.0 mmol), DPTS (0.061 g, 0.21 mmol) and DIPC (0.25 g, 2.01 mmol). ^{1}H NMR (500 MHz, $CDCl_3$): δ (ppm) 8.00 (d, J = 8.0 Hz, 6H), 7.55 (d, J = 8.0 Hz, 6H), 7.46 (d, J = 8.6 Hz, 6H), 6.87 (d, J = 8.6 Hz, 6H), 4.32 (t, J = 6.9 Hz, 2H), 4.32 (t, J = 6.9 Hz, 2H), 4.22 (d, J = 5.8 Hz, 2H), 3.98–3.95 (m, 6H), 3.60 (t, J = 6.6 Hz, 2H), 1.81–1.74 (m, 11H), 1.59–1.28 (m, 62H), 0.91–0.86 (m, 15H), 0.05 (s, 6H). ^{13}C NMR (125 MHz, $CDCl_3$): δ (ppm) 166.28, 166.21, 159.62, 133.23, 131.26, 129.52, 129.44, 128.38, 114.61, 114.53, 92.68, 92.63, 87.50, 68.12, 67.94, 65.27, 63.08, 37.47, 32.79, 31.91, 31.82, 31.45, 29.95, 29.61, 29.55, 29.52, 29.48, 29.44, 29.43, 29.40, 29.35, 29.23, 29.18, 26.77, 26.75, 26.00, 25.73, 25.66, 22.68, 22.66, 18.39, 14.11, −5.23. MALDI-TOF mass: calcd. for $C_{97}H_{133}O_{10}Si$ $[M + H]^+$: m/z = 1485.97; found: 1485.95.

Compound 10. By a procedure similar to that for **4**, compound **10** was obtained in 91% yield (0.92 g, 0.67 mmol) from **9** (1.10 g, 0.74 mmol) and TBAF (1.0 M in THF, 1.50 mL, 1.50 mmol).

^{1}H NMR (500 MHz, $CDCl_3$): δ (ppm) 8.00 (d x 2, J = 8.0 Hz, 4H, overlapped), 7.98 (d, J = 8.0 Hz, 2H), 7.55 (d, J = 8.0 Hz, 6H), 7.46 (d x 3, J = 8.6 Hz, 6H, overlapped), 6.87 (d x 3, J = 8.6 Hz, 6H, overlapped), 4.32 (t x 2, J = 6.9 Hz, 4H, overlapped), 4.22 (d, J = 5.8 Hz, 2H), 3.98–3.95 (m, 6H), 3.63 (br, 2H), 1.81–1.74 (m, 11H), 1.58–1.54 (m, 2H), 1.41–1.28 (m, 61H), 0.89–0.86 (m, 6H). ^{13}C NMR (125 MHz, $CDCl_3$): δ (ppm) 166.27, 166.20, 159.61, 133.23, 131.25, 129.51, 129.45, 129.43, 114.61, 114.52, 114.49, 92.63, 92.64, 87.50, 68.15, 68.08, 67.91, 65.24, 37.45, 32.80, 31.91, 31.88, 31.45, 30.37, 29.95, 29.62, 29.55, 29.47, 29.42, 29.33, 29.24, 29.18, 28.93, 28.70, 26.77, 26.75, 26.01, 22.68, 22.65, 14.12. MALDI-TOF mass: calcd. for $C_{91}H_{119}O_{10}$ $[M + H]^+$: m/z = 1371.88; found: 1371.90.

Compound 11. By a procedure similar to that for **5**, compound **11** was obtained in 60% yield (0.57 g, 0.39 mmol) from **10** (0.90 g, 0.66 mmol), methacryloyl chloride (0.14 g, 1.31 mmol) and triethylamine (0.14 g, 1.35 mmol). ^{1}H NMR (500 MHz, $CDCl_3$): δ (ppm) 8.00 (d, J = 8.0 Hz, 2H), 7.99 (d, J = 8.0 Hz, 2H), 7.55 (d, J = 8.6 Hz, 6H), 7.47 (d, J = 8.6 Hz, 2H), 7.46 (d, J = 8.6 Hz, 2H), 7.46 (d, J = 8.6 Hz, 2H), 6.87 (d, J = 8.6 Hz, 6H), 6.10 (s, 1H), 5.54 (t, J = 1.7 Hz, 1H), 4.32 (t, J = 6.7 Hz, 4H), 4.23 (d, J = 5.7 Hz, 2H), 4.14 (t, J = 6.6 Hz, 2H), 3.97 (t, J = 6.3 Hz, 6H), 1.94 (s, 3H), 1.83–1.74 (m, 11H), 1.70–1.64 (m, 2H), 1.46–1.28 (m, 60H), 0.89–0.86 (m, 6H). ^{13}C NMR (125 MHz, $CDCl_3$): δ (ppm) 167.56, 166.35, 159.60, 136.65, 133.22, 131.26, 129.50, 129.44, 128.35, 125.18, 114.62, 114.54, 92.61, 87.59, 68.10, 65.27, 64.83, 37.50, 31.91, 31.82, 31.46, 29.97, 29.55, 29.45, 29.42, 29.34, 29.31, 29.24, 29.18, 28.73, 28.62, 26.77, 26.01, 25.97, 22.69, 22.65, 18.33, 14.13, 14.11. MALDI-TOF mass: calcd. for $C_{95}H_{123}O_{11}$ $[M + H]^+$: m/z = 1439.91; found: 1440.01.

Synthesis of 17 and 21

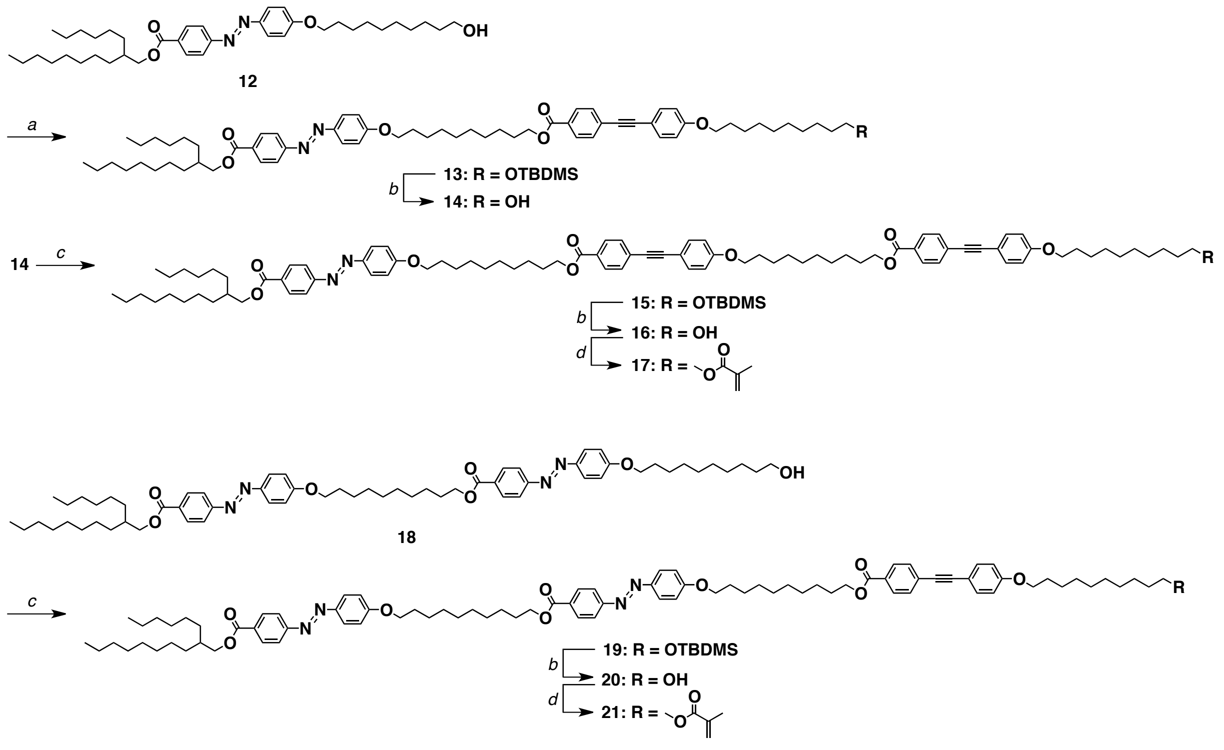

Reagents and conditions: (a) **2**, DPTS, DIPC, CH_2Cl_2, 25 °C; (b) TBAF, THF, 25 °C; (c) **2**, DPTS, DIPC, CH_2Cl_2, 40 °C; (d) methacryloyl chloride, triethylamine, CH_2Cl_2, 25 °C.

Compound 13. By a procedure similar to that for **3**, compound **13** was obtained in 84% yield (1.50 g, 1.34 mmol) from **12** (1.0 g, 1.60 mmol), **2** (0.81 g, 1.60 mmol), DPTS (0.10 g, 0.33 mmol) and DIPC (0.40 g, 3.20 mmol). ^{1}H NMR (500 MHz, $CDCl_3$): δ (ppm) 8.16 (d, J = 8.6 Hz, 2H), 8.00 (d, J = 8.6 Hz, 2H), 7.93 (d, J = 8.6 Hz, 2H), 7.91 (d, J = 8.6 Hz, 2H), 7.55 (d, J = 8.0 Hz, 2H), 7.46 (d, J = 8.6 Hz, 2H), 7.01 (d, J = 8.6 Hz, 2H), 6.86 (d, J = 8.6 Hz, 2H), 4.31 (t, J = 6.9 Hz, 2H), 4.25 (d, J = 5.8 Hz, 2H), 4.04 (t, J = 6.3 Hz, 2H), 3.96 (t, J = 6.3 Hz, 2H), 3.60 (t, J = 6.6 Hz, 2H), 1.85–1.75 (m, 7H), 1.52–1.30 (m, 50H), 0.89–0.86 (m, 15H), 0.05 (s, 6H). ^{13}C NMR (125 MHz, $CDCl_3$): δ (ppm) 166.29, 166.21, 162.33, 159.65, 155.40, 146.91, 133.37, 133.23, 131.67, 131.60, 131.26, 130.51, 129.46, 128.39, 125.20, 122.35, 114.81, 114.71, 114.64, 114.52, 92.68, 87.49, 68.44, 68.13, 68.03, 65.25, 63.32, 37.50, 32.90, 31.92, 31.83, 31.51, 29.98, 29.64, 29.57, 29.51, 29.46, 29.44, 29.38, 29.34, 29.32, 29.24, 29.20, 28.72, 26.80, 26.77, 26.01, 25.82, 24.63, 22.68, 22.66, 18.39, 14.34, 14.12, −5.24. MALDI-TOF mass: calcd. for $C_{70}H_{105}N_2O_7Si$ $[M + H]^+$: m/z = 1113.77; found: 1113.79.

Compound 14. By a procedure similar to that for **4**, compound **14** was obtained in 97% yield (1.22 g, 1.22 mmol) from **13** (1.40 g, 1.26 mmol) and TBAF (1.0 M in THF, 2.60 mL, 2.60 mmol). ^{1}H NMR (500 MHz, $CDCl_3$): δ (ppm) 8.16 (d, J = 8.6 Hz, 2H), 8.00 (d, J = 8.6 Hz, 2H), 7.93 (d, J = 8.6 Hz, 2H), 7.90 (d, J = 8.6 Hz, 2H), 7.55 (d, J = 8.0 Hz, 2H), 7.46 (d, J = 8.6 Hz, 2H), 7.01 (d, J = 8.6 Hz, 2H), 6.86 (d, J = 8.6 Hz, 2H), 4.31 (t, J = 6.9 Hz, 2H), 4.25 (d, J = 5.8 Hz, 2H), 4.05 (t, J = 6.3 Hz, 2H), 3.96 (t, J = 6.3 Hz, 2H), 3.64 (t, J = 6.6 Hz, 2H), 1.85–1.78 (m, 7H), 1.59–1.54 (m, 2H), 1.48–1.29 (m, 49H), 0.88–0.86 (m, 6H). ^{13}C NMR (125 MHz, $CDCl_3$): δ (ppm) 166.31, 166.22, 162.33, 159.64, 155.40, 146.91, 133.23, 131.59, 131.26, 130.51, 129.46, 128.38, 125.20, 122.34, 114.82, 114.64, 114.53, 92.66, 87.49, 68.44, 68.12, 68.04, 65.26, 63.07, 37.49, 32.81, 31.92, 31.82, 31.50, 29.97, 29.64, 29.56, 29.52, 29.47, 29.45, 29.41, 29.35, 29.31, 29.24, 29.18, 28.71, 26.80, 26.79, 26.01, 25.74, 23.50, 22.68, 22.66, 14.11. MALDI-TOF mass: calcd. for $C_{64}H_{91}N_2O_7Si$ $[M + H]^+$: m/z = 999.68; found: 999.67.

Compound 15. By a procedure similar to that for **3**, except that the reaction temperature was 40 °C, compound **15** was obtained in 90% yield (1.34 g, 0.90 mmol) from **14** (1.0 g, 1.0 mmol), **2** (0.55 g, 1.10 mmol), DPTS (0.061 g, 0.21 mmol) and DIPC (0.25 g, 2.01 mmol). ^{1}H NMR (500 MHz, $CDCl_3$): δ (ppm) 8.16 (d, J = 8.6 Hz, 2H), 8.00 (d, J = 8.6 Hz, 4H), 7.94 (d, J = 8.6 Hz, 2H), 7.90 (d, J = 8.6 Hz, 2H), 7.55 (d, J = 8.6 Hz, 4H), 7.46 (d, J = 8.6 Hz, 2H), 7.46 (d, J = 8.6 Hz, 2H), 7.01 (d, J = 8.6 Hz, 2H), 6.87 (d, J = 8.6 Hz, 4H), 4.32 (t, J = 6.6 Hz, 4H), 4.26 (d, J = 5.8 Hz, 2H), 4.05 (t, J = 6.6 Hz, 2H), 3.96 (t, J = 6.6 Hz, 4H), 3.60 (t, J = 6.6 Hz, 2H), 1.85–1.76 (m, 11H), 1.52–1.30 (m, 62H), 0.90–0.86 (m, 15H), 0.05 (s, 6H). ^{13}C NMR (125 MHz, $CDCl_3$): δ (ppm) 166.29, 166.21, 162.33, 159.65, 155.40, 146.92, 133.24, 131.60, 131.26, 130.51, 129.46, 128.38, 125.20, 122.35, 114.82, 114.64, 114.53, 92.67, 87.49, 68.44, 68.11, 68.03, 65.25, 63.32, 37.50, 32.90, 31.92, 31.83, 31.51, 29.97, 29.64, 29.57, 29.51, 29.45, 29.43, 29.37, 29.34, 29.32, 29.24, 29.20, 28.72, 26.80, 26.77, 26.02, 25.82, 22.68, 22.66, 18.39, 14.12, −5.24. MALDI-TOF mass: calcd. for $C_{95}H_{133}N_2O_{10}Si$ $[M + H]^+$: m/z = 1489.97; found: 1489.94.

Compound 16. By a procedure similar to that for **4**, compound **16** was obtained in 90% yield (1.24 g, 0.90 mmol) from **15** (1.20 g, 0.81 mmol) and TBAF (1.0 M in THF, 2.0 mL, 2.0 mmol). ^{1}H NMR (500 MHz, $CDCl_3$): δ (ppm) 8.16 (d, J = 8.6 Hz, 2H), 8.00 (d, J = 8.6 Hz, 4H), 7.94 (d, J = 8.6 Hz, 2H), 7.90 (d, J = 8.6 Hz, 2H), 7.55 (d, J = 8.0 Hz, 4H), 7.46 (d, J = 8.6 Hz, 2H), 7.46 (d, J = 8.6 Hz, 2H), 7.01 (d, J = 8.6 Hz, 2H), 6.87 (d, J = 8.0 Hz, 4H), 4.32 (t, J = 6.6 Hz, 4H), 4.25 (d, J = 5.8 Hz, 2H), 4.05 (t, J = 6.3 Hz, 2H), 3.96 (t, J = 6.6 Hz, 4H), 3.64 (br, J = 6.6 Hz, 2H), 1.84–1.75 (m, 11H), 1.58–1.55 (m, 2H), 1.46–1.22 (m, 61H), 0.88–0.86 (m, 6H). ^{13}C NMR (125 MHz, $CDCl_3$): δ (ppm) 166.29, 166.21, 162.33, 159.64, 155.42, 146.91, 133.24, 131.61, 131.26, 130.51, 129.46, 128.38, 125.20, 122.34, 114.82, 114.64, 114.53, 92.67, 87.50, 68.44, 68.12, 68.04, 65.26, 63.08, 37.50, 32.92, 31.92, 31.83, 31.50, 29.97, 29.64, 29.57, 29.52, 29.45,

29.43, 29.41, 29.35, 29.24, 29.19, 28.72, 26.80, 26.77, 26.02, 25.74, 22.68, 22.66, 14.10. MALDI-TOF mass: calcd. for $C_{89}H_{119}N_2O_{10}$ $[M + H]^+$: m/z = 1375.89; found: 1375.82.

Compound 17. By a procedure similar to that for **5**, compound **17** was obtained in 60% yield (0.63 g, 0.44 mmol) from **16** (1.0 g, 0.73 mmol), methacryloyl chloride (0.15 g, 1.50 mmol) and triethylamine (0.16 g, 1.50 mmol). ^{1}H NMR (500 MHz, $CDCl_3$): δ (ppm) 8.16 (d, J = 8.6 Hz, 2H), 8.00 (d, J = 8.0 Hz, 4H), 7.93 (d, J = 8.6 Hz, 2H), 7.90 (d, J = 8.6 Hz, 2H), 7.55 (d, J = 8.0 Hz, 4H), 7.46 (d, J = 8.6 Hz, 2H), 7.46 (d, J = 8.6 Hz, 2H), 7.01 (d, J = 8.6 Hz, 2H), 6.87 (d, J = 8.0 Hz, 4H), 6.10 (s, 1H), 5.54 (t, J = 1.7 Hz, 1H), 4.32 (t, J = 6.6 Hz, 4H), 4.25 (d, J = 5.8 Hz, 2H), 4.14 (t, J = 6.3 Hz, 2H), 4.05 (t, J = 6.3 Hz, 2H), 3.97 (t, J = 6.6 Hz, 4H), 1.94 (s, 3H), 1.85−1.76 (m, 11H), 1.70−1.64 (m, 2H), 1.45−1.29 (m, 60H), 0.88−0.86 (m, 6H). ^{13}C NMR (125 MHz, $CDCl_3$): δ (ppm) 166.30, 166.21, 162.33, 159.65, 155.42, 146.91, 136.60, 133.24, 131.62, 131.26, 130.51, 129.47, 128.38, 125.21, 122.34, 114.82, 114.64, 114.53, 92.67, 87.50, 68.44, 68.12, 68.04, 65.26, 63.09, 37.50, 32.92, 31.92, 31.83, 31.50, 29.97, 29.64, 29.58, 29.52, 29.46, 29.43, 29.41, 29.35, 29.25, 29.19, 28.72, 26.80, 26.77, 26.02, 25.74, 22.68, 22.66, 14.11. MALDI-TOF mass: calcd. for $C_{93}H_{123}N_2O_{11}$ $[M + H]^+$: m/z = 1443.91; found: 1443.89.

Compound 19. By a procedure similar to that for **3**, except that the reaction temperature was 40 °C, compound **19** was obtained in 95% yield (1.42 g, 0.95 mmol) from **18** (1.0 g, 1.0 mmol), **2** (0.55 g, 1.10 mmol), DPTS (0.060 g, 0.210 mmol) and DIPC (0.25 g, 1.98 mmol). ^{1}H NMR (500 MHz, $CDCl_3$): δ (ppm) 8.17 (d, J = 8.6 Hz, 2H), 8.16 (d, J = 8.6 Hz, 2H), 8.00 (d, J = 8.6 Hz, 2H), 7.93 (d, J = 8.6 Hz, 2H), 7.93 (d, J = 8.6 Hz, 2H), 7.90 (d, J = 8.6 Hz, 2H), 7.90 (d, J = 8.6 Hz, 2H), 7.55 (d, J = 8.0 Hz, 2H), 7.46 (d, J = 8.6 Hz, 2H), 7.00 (d , J = 8.6 Hz, 2H), 7.00 (d , J = 8.6 Hz, 2H), 6.87 (d, J = 8.6 Hz, 2H), 4.35 (t, J = 6.9 Hz, 2H), 4.32 (t, J = 6.6 Hz, 2H), 4.26 (d, J = 5.8 Hz, 2H), 4.04 (t, J = 6.6 Hz, 4H), 4.04 (t, J = 6.6 Hz, 4H), 3.96 (t, J = 6.6 Hz, 2H), 3.60 (t, J = 6.6 Hz, 2H), 1.81−1.78 (m, 11H), 1.56−1.22 (m, 62H), 0.90−0.86 (m, 15H), 0.05 (s, 6H). ^{13}C NMR (125 MHz, $CDCl_3$): δ (ppm) 166.32, 166.21, 162.38, 159.65, 155.40, 146.98, 133.23, 131.60, 130.53, 130.51, 129.45, 128.38, 125.20, 122.34, 122.32, 114.82, 114.64, 92.70, 87.52, 68.44, 68.14, 65.25, 63.32, 37.50, 32.90, 31.83, 31.50, 29.97, 29.64, 29.56, 29.50, 29.45, 29.43, 29.37, 29.34, 29.31, 29.24, 29.19, 28.72, 26.77, 26.02, 25.82, 22.68, 22.65, 18.39, 14.11, −5.24. MALDI-TOF mass: calcd. for $C_{93}H_{133}N_4O_{10}Si$ $[M + H]^+$: m/z = 1493.98; found: 1493.90.

Compound 20. By a procedure similar to that for **4**, compound **20** was obtained in 84% yield (1.01 g, 0.73 mmol) from **19** (1.30 g, 0.87 mmol) and TBAF (1.0 M in THF, 2.0 mL, 2.0 mmol). ^{1}H NMR (500 MHz, $CDCl_3$): δ (ppm) 8.17 (d, J = 8.6 Hz, 2H), 8.16 (d, J = 8.6 Hz, 2H), 8.00 (d, J = 8.6 Hz, 2H), 7.93 (d, J = 8.6 Hz, 2H), 7.93 (d, J = 8.6 Hz, 2H), 7.90 (d, J = 8.6 Hz, 2H), 7.90 (d, J = 8.6 Hz, 2H), 7.55 (d, J = 8.6 Hz, 2H), 7.46 (d, J = 8.6 Hz, 2H), 7.01 (d, J = 8.6 Hz, 2H), 7.01 (d, J = 8.6 Hz, 2H), 6.87 (d, J = 8.6 Hz, 2H), 4.34 (t, J = 6.9 Hz, 2H), 4.32 (t, J = 6.6 Hz,

2H), 4.26 (d, J = 5.8 Hz, 2H), 4.04 (t, J = 6.6 Hz, 4H), 4.04 (t, J = 6.6 Hz, 4H), 3.97 (t, J = 6.6 Hz, 2H), 3.64 (q, J = 5.8 Hz, 2H), 1.82–1.77 (m, 11H), 1.48–1.45 (m, 2H), 1.44–1.22 (m, 61H), 0.90–0.86 (m, 6H). ^{13}C NMR (125 MHz, $CDCl_3$): δ (ppm) 166.28, 166.21, 162.36, 159.66, 155.45, 146.95, 133.24, 131.27, 130.54, 130.52, 129.46, 128.37, 125.20, 124.38, 122.35, 122.33, 114.83, 114.65, 92.67, 87.49, 68.46, 68.14, 65.25, 63.10, 37.55, 32.82, 31.92, 31.83, 31.51, 29.57, 29.52, 29.48, 29.46, 29.35, 29.32, 29.24, 29.19, 28.72, 26.78, 26.02, 25.75, 22.68, 22.66, 14.10. MALDI-TOF mass: calcd. for $C_{87}H_{119}N_4O_{10}$ $[M + H]^+$: m/z = 1379.89; found: 1379.86.

Compound 21. By a procedure similar to that for **5**, compound **21** was obtained in 65% yield (0.61 g, 0.42 mmol) from **20** (0.90 g, 0.65 mmol), methacryloyl chloride (0.14 g, 1.30 mmol) and triethylamine (0.14 g, 1.36 mmol). ^{1}H NMR (500 MHz, $CDCl_3$): δ (ppm) 8.17 (d, J = 8.6 Hz, 2H), 8.16 (d, J = 8.6 Hz, 2H), 8.00 (d, J = 8.0 Hz, 2H), 7.93 (d, J = 8.6 Hz, 4H), 7.90 (d, J = 8.6 Hz, 4H), 7.55 (d, J = 8.6 Hz, 2H), 7.46 (d, J = 8.6 Hz, 2H), 7.01 (d, J = 8.6 Hz, 2H), 7.01 (d, J = 8.6 Hz, 2H), 6.87 (d, J = 8.6 Hz, 2H), 6.10 (s, 1H), 5.54 (t, J = 1.7 Hz, 1H), 4.35 (t, J = 6.6 Hz, 2H), 4.32 (t, J = 6.7 Hz, 2H), 4.25 (d, J = 5.7 Hz, 2H), 4.14 (t, J = 6.3 Hz, 2H), 4.05 (t, J = 6.3 Hz, 4H), 3.97 (t, J = 6.6 Hz, 2H), 1.94 (s, 3H), 1.82–1.76 (m, 11H), 1.70–1.64 (m, 2H), 1.47–1.27 (m, 60H), 0.89–0.86 (m, 6H). ^{13}C NMR (125 MHz, $CDCl_3$): δ (ppm) 166.30, 166.22, 162.33, 159.65, 155.43, 146.91, 136.58, 133.24, 131.62, 131.26, 130.51, 129.49, 128.38, 125.21, 122.33, 114.82, 114.64, 114.54, 92.67, 87.51, 68.44, 68.12, 68.04, 65.26, 63.11, 37.50, 32.92, 31.94, 31.83, 31.50, 29.95, 29.64, 29.58, 29.52, 29.46, 29.43, 29.43, 29.35, 29.27, 29.19, 28.72, 26.81, 26.77, 26.02, 25.73, 22.68, 22.66, 14.11. MALDI-TOF mass: calcd. for $C_{91}H_{123}N_4O_{11}$ $[M + H]^+$: m/z = 1447.92; found: 1447.93.

Synthesis of 26, 29, 32, 33 and 34

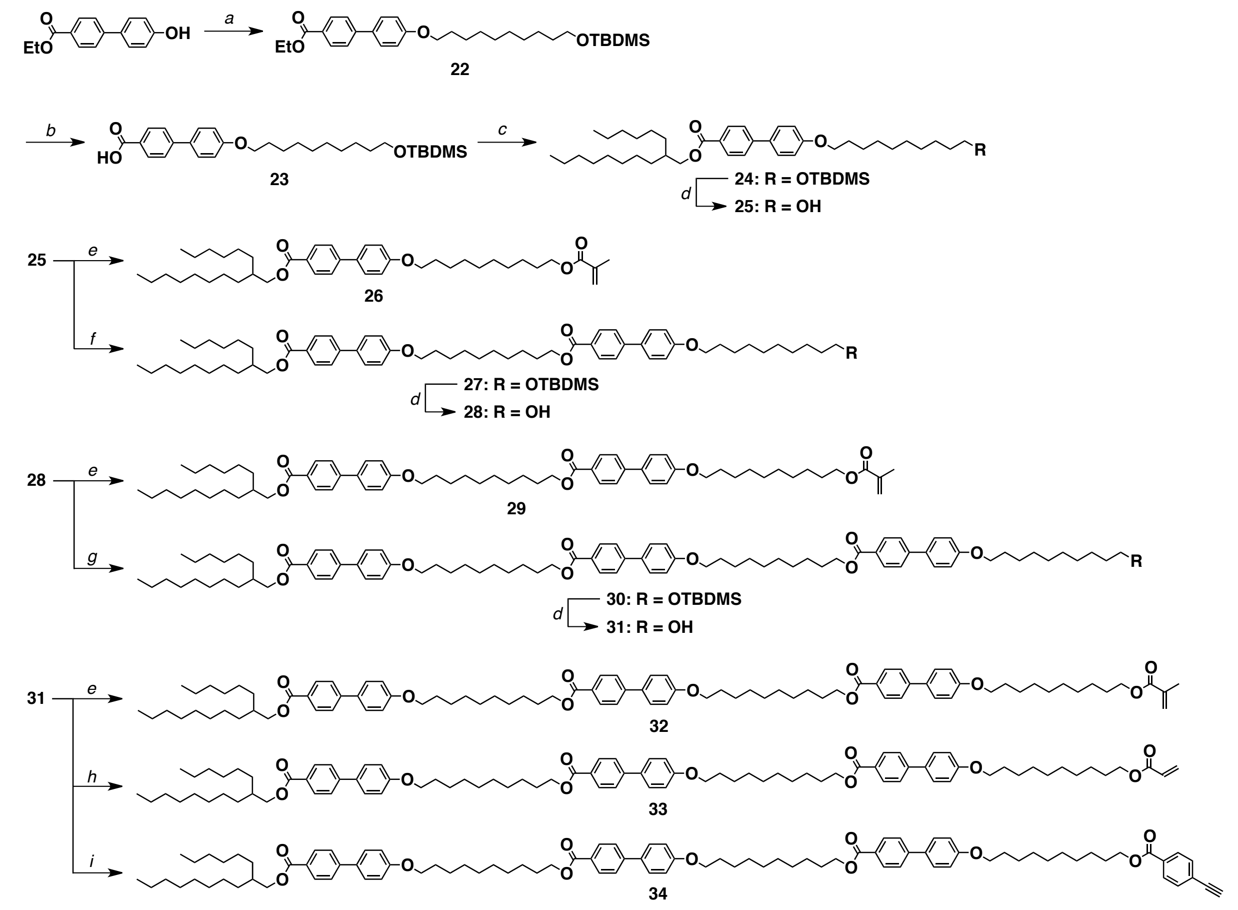

Reagents and conditions: (a) 10-bromodecyl *tert*-butyldimethylsilyl ether, Cs_2CO_3, DMF, 70 °C, (b) KOH, THF, 50 °C; (c) 2-hexyl-1-decanol, DPTS, DIPC, CH_2Cl_2, 25 °C; (d) TBAF, THF, 25 °C; (e) methacryloyl chloride, triethylamine, CH_2Cl_2, 25 °C; (f) **23**, DPTS, DIPC, CH_2Cl_2, 25 °C; (g) **23**, DPTS, DIPC, CH_2Cl_2, 40 °C; (h) acryloyl chloride, triethylamine, CH_2Cl_2, 25 °C; (i) 4-ethynylbenzoic acid, DPTS, DIPC, CH_2Cl_2, 25 °C.

Compound 22. To a DMF solution (110 mL) of ethyl 4-hydroxy-4'-biphenylcarboxylate (10.8 g, 44.5 mmol) were successively added 10-bromodecyl *tert*-butyldimethylsilyl ether (16.4 g, 46.7 mmol) and Cs_2CO_3 (17.4 g, 53.4 mmol), and the mixture was stirred at 70 °C for 15 h under N_2. The reaction mixture was allowed to cool to 25 °C and filtrated off from an insoluble fraction. The filtrate was diluted with AcOEt, washed successively with a saturated aqueous solution of NH_4Cl, water and brine, and an organic phase separated was dried over anhydrous $MgSO_4$ and then evaporated to dryness under a reduced pressure. The residue was recrystallized from a mixture of AcOEt/MeOH, affording **22** as white solid (19.0 g, 37.0 mmol) in 83% yield. ^{1}H NMR (500 MHz, $CDCl_3$): δ (ppm) 8.06 (d, J = 8.6 Hz, 2H), 7.59 (d, J = 8.6 Hz, 2H), 7.54 (d, J = 8.6 Hz, 2H), 6.96 (d, J = 8.6 Hz, 2H), 4.37 (q, J = 7.1 Hz, 2H), 3.98 (t, J = 6.6 Hz, 2H), 3.68 (t, J

= 6.6 Hz, 2H), 1.79 (m, 2H), 1.49 (m, 4H), 1.39 (t, J = 6.9 Hz, 3H), 1.28 (br, 10H), 0.88 (s, 9H), 0.03 (s, 6H). ^{13}C NMR (125 MHz, $CDCl_3$): δ (ppm) 166.75, 159.55, 145.33, 132.35, 130.19, 128.67, 128.46, 126.53, 115.07, 68.28, 63.48, 61.03, 33.04, 29.72, 29.67, 29.57, 29.54, 29.40, 26.19, 26.14, 25.95, 18.54, 14.52, −5.10. MALDI-TOF mass: calcd. for $C_{27}H_{39}O_4Si$ $[M - C_4H_9]^+$: m/z = 455.26; found: 455.30.

Compound 23. To a THF solution (240 mL) of **22** (6.1 g, 12.0 mmol) were successively added EtOH (48 mL) and an aqueous solution of KOH (1 M, 24 mL), and the mixture was stirred at 50 °C for 10 h under N_2. The reaction mixture was poured into a saturated aqueous solution of NH_4Cl and extracted with $CHCl_3$. The combined organic extract was washed successively with water and brine, dried over anhydrous $MgSO_4$ and then evaporated to dryness under a reduced pressure. The residue was recrystallized from EtOH, affording **23** as white solid (4.5 g, 9.3 mmol) in 78% yield. ^{1}H NMR (500 MHz, DMSO-d_6, 60 °C): δ (ppm) 7.98 (d, J = 8.1 Hz, 2H), 7.73 (d, J = 8.0 Hz, 2H), 7.66 (d, J = 8.6 Hz, 2H), 7.03 (d, J = 8.6 Hz, 2H), 4.03 (t, J = 6.3 Hz, 2H), 3.57 (t, J = 6.3 Hz, 2H), 1.73 (m, 2H), 1.44 (br, 4H), 1.29 (br, 10H), 0.87 (s, 9H), 0.02 (s, 6H). ^{13}C NMR (125 MHz, DMSO-d_6, 60 °C): δ (ppm) 167.43, 158.72, 143.17, 131.20, 130.21, 129.60, 127.76, 125.61, 114.85, 67.47, 62.23, 31.99, 28.63, 28.58, 28.41, 25.56, 25.20, 24.97, 17.64, −5.56. MALDI-TOF mass: calcd. for $C_{29}H_{44}NaO_4Si$ $[M + Na]^+$: m/z = 507.29; found: 507.31.

Compound 24. By a procedure similar to that for **3**, compound **24** was obtained in 91% yield (2.40 g, 3.38 mmol) from **23** (1.80 g, 3.71 mmol), 2-hexyl-1-decanol (0.95 g, 3.92 mmol), DPTS (0.17 g, 0.59 mmol) and DIPC (0.51 g, 3.95 mmol). ^{1}H NMR (500 MHz, $CDCl_3$): δ (ppm) 8.08 (d, J = 8.6 Hz, 2H), 7.62 (d, J = 8.6 Hz, 2H), 7.55 (d, J = 8.6 Hz, 2H), 6.98 (d, J = 8.6 Hz, 2H), 4.24 (d, J = 5.7 Hz, 2H), 4.01 (t, J = 6.8 Hz, 2H), 3.60 (t, J = 6.7 Hz, 2H), 1.79–1.77 (m, 4H), 1.56–1.12 (m, 37H), 0.89–0.86 (m, 15H), 0.05 (s, 6H). ^{13}C NMR (125 MHz, $CDCl_3$): δ (ppm) 166.71, 159.43, 145.24, 132.26, 130.06, 129.04, 128.38, 126.34, 122.34, 114.96, 68.18, 67.64, 63.34, 37.52, 32.92, 31.96, 31.83, 31.47, 29.96, 29.66, 29.56, 29.45, 29.43, 29.41, 29.35, 29.24, 29.20, 28.72, 26.80, 26.76, 26.05, 25.98, 25.76, 22.68, 22.66, 18.42, 14.11, −5.24. MALDI-TOF mass: calcd. for $C_{45}H_{77}O_4Si$ $[M + H]^+$: m/z = 709.56; found: 709.55.

Compound 25. By a procedure similar to that for **4**, compound **25** was obtained in 96% yield (1.85 g, 3.11 mmol) from **24** (2.30 g, 3.24 mmol) and TBAF (1.0 M in THF, 6.0 mL, 6.0 mmol). ^{1}H NMR (500 MHz, $CDCl_3$): δ (ppm) 8.08 (d, J = 8.6 Hz, 2H), 7.61 (d, J = 8.6 Hz, 2H), 7.55 (d, J = 8.6 Hz, 2H), 6.99 (d, J = 8.6 Hz, 2H), 4.24 (d, J = 5.7 Hz, 2H), 4.01 (t, J = 6.8 Hz, 2H), 3.65 (br, 2H), 1.83–1.77 (m, 4H), 1.62–1.55 (m, 2H), 1.53–1.18 (m, 36H), 0.88–0.86 (m, 6H). ^{13}C NMR (125 MHz, $CDCl_3$): δ (ppm) 166.71, 159.43, 145.25, 132.26, 130.06, 129.04, 128.38, 126.34, 122.33, 114.97, 68.18, 67.64, 63.33, 37.54, 32.93, 31.96, 31.83, 31.47, 29.96, 29.66,

29.56, 29.45, 29.43, 29.40, 29.35, 29.24, 29.20, 28.72, 26.80, 26.76, 26.05, 25.96, 25.76, 22.68, 22.66, 14.11. MALDI-TOF mass: calcd. for $C_{39}H_{63}O_4$ $[M + H]^+$: m/z = 595.47; found: 595.46.

Compound 26. By a procedure similar to that for **5**, compound **26** was obtained in 75% yield (0.50 g, 0.75 mmol) from **25** (0.60 g, 1.01 mmol), methacryloyl chloride (0.21 g, 2.05 mmol) and triethylamine (0.21 g, 1.98 mmol). ^{1}H NMR (500 MHz, $CDCl_3$): δ (ppm) 8.08 (d, J = 8.0 Hz, 2H), 7.61 (d, J = 8.6 Hz, 2H), 7.55 (d, J = 8.6 Hz, 2H), 6.98 (d, J = 8.6 Hz, 2H), 6.09 (s, 1H), 5.54 (d, J = 1.7 Hz, 1H), 4.24 (d, J = 5.7 Hz, 2H), 4.14 (t, J = 6.8 Hz, 2H), 4.01 (t, J = 6.8 Hz, 2H), 1.94 (s, 3H), 1.83–1.78 (m, 2H), 1.70–1.62 (m, 2H), 1.50–1.22 (m, 37H), 0.91–0.86 (m, 6H). ^{13}C NMR (125 MHz, $CDCl_3$): δ (ppm) 167.56, 166.72, 159.41, 145.18, 136.62, 136.58, 132.25, 130.05, 128.63, 128.31, 126.42, 125.12, 125.10, 114.94, 68.11, 67.70, 67.49, 64.81, 37.50, 37.34, 31.92, 31.84, 31.81, 31.50, 31.42, 29.98, 29.95, 29.65, 29.61, 29.57, 29.47, 29.44, 29.36, 29.32, 29.26, 29.24, 28.62, 26.79, 26.77, 26.74, 26.71, 26.05, 25.98, 25.71, 22.68, 22.66, 18.33, 14.11. MALDI-TOF mass: calcd. for $C_{43}H_{67}O_5$ $[M + H]^+$: m/z = 663.50; found: 663.51.

Compound 27. By a procedure similar to that for **3**, compound **27** was obtained in 60% yield (1.28 g, 1.21 mmol) from **23** (1.02 g, 2.10 mmol), **25** (1.20 g, 2.02 mmol), DPTS (0.10 g, 0.33 mmol) and DIPC (0.38 g, 3.00 mmol). ^{1}H NMR (500 MHz, $CDCl_3$): δ (ppm) 8.08 (d, J = 8.6 Hz, 4H), 7.62 (d, J = 8.6 Hz, 4H), 7.55 (d, J = 8.6 Hz, 4H), 6.99 (d, J = 8.6 Hz, 4H), 4.34 (t, J = 6.9 Hz, 2H), 4.24 (d, J = 5.7 Hz, 2H), 4.01 (t, J = 6.8 Hz, 4H), 3.60 (t, J = 6.8 Hz, 2H), 1.79–1.77 (m, 7H), 1.56–1.22 (m, 50H), 0.91–0.86 (m, 15H), 0.05 (s, 6H). ^{13}C NMR (125 MHz, $CDCl_3$): δ (ppm) 167.53, 166.76, 159.47, 145.18, 136.62, 132.27, 130.05, 128.62, 128.31, 126.42, 125.12, 114.94, 68.11, 67.70, 67.49, 64.83, 37.50, 37.34, 31.92, 31.84, 31.80, 31.52, 31.42, 29.98, 29.95, 29.65, 29.60, 29.57, 29.48, 29.44, 29.36, 29.32, 29.26, 29.24, 28.62, 26.79, 26.77, 26.74, 26.71, 26.06, 25.98, 25.74, 22.68, 22.66, 18.34, 14.11, −5.24. MALDI-TOF mass: calcd. for $C_{68}H_{105}O_7Si$ $[M + H]^+$: m/z = 1061.76; found: 1061.77.

Compound 28. By a procedure similar to that for **4**, compound **28** was obtained in 90% yield (1.02 g, 1.08 mmol) from **27** (1.20 g, 1.13 mmol) and TBAF (1.0 M in THF, 2.0 mL, 2.0 mmol). ^{1}H NMR (500 MHz, $CDCl_3$): δ (ppm) 8.08 (d, J = 8.6 Hz, 4H), 7.62 (d, J = 8.6 Hz, 4H), 7.55 (d, J = 8.6 Hz, 4H), 6.98 (d, J = 8.6 Hz, 4H), 4.33 (t, J = 6.9 Hz, 2H), 4.24 (d, J = 5.7 Hz, 2H), 4.01 (t, J = 6.8 Hz, 4H), 3.65 (t, J = 6.9 Hz, 2H), 1.83–1.77 (m, 7H), 1.62–1.55 (m, 2H), 1.53–1.22 (m, 49H), 0.90–0.86 (m, 6H). ^{13}C NMR (125 MHz, $CDCl_3$): δ (ppm) 167.53, 166.75, 159.48, 145.18, 136.63, 132.27, 130.05, 128.64, 128.32, 126.42, 126.12, 114.98, 68.11, 67.70, 65.02, 63.04, 37.50, 37.34, 31.92, 31.84, 31.80, 31.52, 31.42, 29.98, 29.95, 29.65, 29.60, 29.57, 29.48, 29.45, 29.36, 29.32, 29.27, 29.24, 28.62, 26.79, 26.78, 26.74, 26.71, 26.06, 25.98, 25.74, 22.68, 22.66, 14.11. MALDI-TOF mass: calcd. for $C_{62}H_{91}O_7$ $[M + H]^+$: m/z = 947.68; found: 947.67.

Compound 29. By a procedure similar to that for **5**, compound **29** was obtained in 76% yield (0.41 g, 0.40 mmol) from **28** (0.50 g, 0.53 mmol), methacryloyl chloride (0.11 g, 1.01 mmol) and triethylamine (0.11 g, 1.09 mmol). ^{1}H NMR (500 MHz, $CDCl_3$): δ (ppm) 8.08 (d, J = 8.6 Hz, 2H), 8.07 (d, J = 8.6 Hz, 2H), 7.61 (d, J = 8.6 Hz, 4H), 7.55 (d, J = 8.6 Hz, 2H), 7.55 (d, J = 8.6 Hz, 2H), 6.98 (d, J = 8.6 Hz, 2H), 6.98 (d, J = 8.6 Hz, 2H), 6.09 (s, 1H), 5.54 (t, J = 1.7 Hz, 1H), 4.33 (d, J = 6.8 Hz, 2H), 4.24 (d, J = 5.7 Hz, 2H), 4.14 (t, J = 6.8 Hz, 2H), 4.00 (t, J = 6.8 Hz, 4H), 1.94 (s, 3H), 1.83–1.75 (m, 7H), 1.70–1.64 (m, 2H), 1.50–1.25 (m, 48H), 0.89–0.85 (m, 6H). ^{13}C NMR (125 MHz, $CDCl_3$): δ (ppm) 167.81, 166.73, 159.41, 145.19, 136.58, 132.22, 130.05, 128.63, 128.31, 126.41, 125.13, 114.94, 68.12, 67.70, 65.06, 64.82, 37.50, 31.92, 31.83, 31.49, 29.97, 29.65, 29.57, 29.47, 29.44, 29.36, 29.32, 29.25, 29.24, 28.76, 28.62, 26.77, 26.05, 25.97, 22.68, 22.66, 18.34, 14.11. MALDI-TOF mass: calcd. for $C_{66}H_{95}O_8$ $[M + H]^+$: m/z = 1015.70; found: 1015.68.

Compound 30. By a procedure similar to that for **3**, except that the reaction temperature was 40 °C, compound **30** was obtained in 93% yield (0.69 g, 0.49 mmol) from **28** (0.50 g, 0.53 mmol), **23** (0.33 g, 0.68 mmol), DPTS (0.10 g, 0.33 mmol) and DIPC (0.31 g, 2.42 mmol). ^{1}H NMR (500 MHz, $CDCl_3$): δ (ppm) 8.08 (d, J = 8.6 Hz, 4H), 8.07 (d, J = 8.6 Hz, 2H), 7.61 (d, J = 8.6 Hz, 6H), 7.55 (d, J = 8.6 Hz, 6H), 6.98 (d, J = 8.6 Hz, 6H), 4.34 (t, J = 6.9 Hz, 4H), 4.24 (d, J = 5.7 Hz, 2H), 4.00 (t, J = 6.8 Hz, 6H), 3.60 (t, J = 6.8 Hz, 2H), 1.84–1.75 (m, 11H), 1.56–1.22 (m, 62H), 0.91–0.86 (m, 15H), 0.05 (s, 6H). ^{13}C NMR (125 MHz, $CDCl_3$): δ (ppm) 167.51, 166.76, 159.43, 145.18, 136.62, 132.27, 130.05, 128.62, 128.30, 126.42, 125.12, 114.94, 68.12, 67.70, 67.49, 64.84, 37.50, 37.38, 31.92, 31.84, 31.80, 31.54, 31.42, 29.98, 29.95, 29.64, 29.60, 29.56, 29.48, 29.44, 29.34, 29.32, 29.26, 29.24, 28.62, 26.79, 26.77, 26.74, 26.71, 26.06, 25.98, 25.74, 22.68, 22.66, 18.34, 14.11, −5.24. MALDI-TOF mass: calcd. for $C_{91}H_{133}O_{10}Si$ $[M + H]^+$: m/z = 1413.97; found: 1413.96.

Compound 31. By a procedure similar to that for **4**, compound **31** was obtained in 94% yield (0.56 g, 0.43 mmol) from **30** (0.65 g, 0.46 mmol) and TBAF (1.0 M in THF, 1.0 mL, 1.0 mmol). ^{1}H NMR (500 MHz, $CDCl_3$): δ (ppm) 8.08 (d, J = 8.6 Hz, 2H), 8.08 (d, J = 8.6 Hz, 2H), 8.07 (d, J = 8.6 Hz, 2H), 7.61 (d, J = 8.6 Hz, 6H), 7.55 (d, J = 8.6 Hz, 6H), 6.98 (d, J = 8.6 Hz, 6H), 4.34 (t, J = 6.9 Hz, 4H), 4.24 (d, J = 5.7 Hz, 2H), 4.00 (t, J = 6.8 Hz, 6H), 3.65 (t, J = 6.8 Hz, 2H), 1.83–1.77 (m, 11H), 1.62–1.55 (m, 2H), 1.53–1.22 (m, 61H), 0.91–0.86 (m, 6H). ^{13}C NMR (125 MHz, $CDCl_3$): δ (ppm) 167.52, 166.75, 159.48, 145.18, 136.63, 132.27, 130.06, 128.64, 126.44, 126.12, 114.98, 68.12, 67.70, 65.04, 63.04, 37.50, 37.35, 31.92, 31.85, 31.52, 31.42, 29.98, 29.95, 29.65, 29.61, 29.57, 29.48, 29.45, 29.37, 29.32, 29.27, 28.62, 26.79, 26.78, 26.74, 26.71, 26.06, 25.98, 25.74, 22.68, 22.66, 14.11. MALDI-TOF mass: calcd. for $C_{85}H_{119}O_{10}$ $[M + H]^+$: m/z = 1299.88; found: 1299.90.

Compound 32. By a procedure similar to that for **5**, compound **32** was obtained in 60% yield (0.35 g, 0.25 mmol) from **31** (0.55 g, 0.42 mmol), methacryloyl chloride (0.10 g, 1.0 mmol) and triethylamine (0.12 g, 1.10 mmol). ^{1}H NMR (500 MHz, $CDCl_3$): δ (ppm) 8.08 (d, J = 8.6 Hz, 2H), 8.07 (d, J = 8.6 Hz, 2H), 8.07 (d, J = 8.6 Hz, 2H), 7.61 (d, J = 8.6 Hz, 6H), 7.55 (d, J = 8.6 Hz, 6H), 6.98 (d, J = 8.6 Hz, 6H), 6.09 (s, 1H), 5.54 (t, J = 1.7 Hz, 1H), 4.33 (t, J = 6.8 Hz, 2H), 4.33 (t, J = 6.8 Hz, 2H), 4.24 (d, J = 5.7 Hz, 2H), 4.14 (t, J = 6.8 Hz, 2H), 4.00 (t, J = 6.8 Hz, 6H), 1.94 (s, 3H), 1.83–1.75 (m, 11H), 1.70–1.64 (m, 2H), 1.48–1.27 (m, 60H), 0.89–0.86 (m, 6H). ^{13}C NMR (125 MHz, $CDCl_3$): δ (ppm) 167.82, 166.73, 159.42, 145.19, 136.58, 132.24, 130.05, 128.63, 128.31, 126.41, 125.13, 114.94, 68.12, 67.71, 65.06, 64.85, 37.52, 31.92, 31.83, 31.49, 29.96, 29.65, 29.58, 29.47, 29.44, 29.36, 29.32, 29.25, 29.25, 28.77, 28.62, 26.74, 26.05, 25.97, 22.68, 22.66, 14.13, 14.11. MALDI-TOF mass: calcd. for $C_{89}H_{123}O_{11}$ $[M + H]^+$: m/z = 1367.91; found: 1367.88.

Compound 33. By a procedure similar to that for **5**, compound **33** was obtained in 67% yield (0.70 g, 0.52 mmol) from **31** (1.00 g, 0.77 mmol), acryloyl chloride (0.42 g, 4.6 mmol) and triethylamine (0.50 g, 4.6 mmol). ^{1}H NMR (500 MHz, $CDCl_3$): δ (ppm) 8.05 (d, J = 8.6 Hz, 6H), 7.60 (d, J = 8.6 Hz, 6H), 7.53 (d, J = 8.6 Hz, 6H), 6.92 (d, J = 8.6 Hz, 6H), 6.34 (dd, J = 1.8 and 17.8 Hz, 1H), 6.07 (dd, J = 10.9 and 17.8 Hz, 1H), 5.75 (dd, J = 1.8 and 10.9 Hz, 1H), 4.28 (t , J = 5.7 Hz, 4H), 4.19 (d, J = 5.7 Hz, 2H), 4.10 (t, J = 6.8 Hz, 2H), 3.94 (m, 6H), 1.78–1.74 (m, 10H), 1.53–1.25 (m, 63H), 0.85–0.81 (m, 6H). ^{13}C NMR (125 MHz, $CDCl_3$): δ (ppm) 166.95, 166.91, 159.62, 145.44, 138.10, 132.01, 130.64, 130.28, 128.87, 128.54, 126.61, 126.30, 115.18, 68.44, 67.88, 65.32, 64.89, 37.73, 32.12, 32.04, 31.72, 29.91, 29.87, 29.73, 29.68, 29.61, 29.53, 29.49, 29.45, 29.04, 28.91, 27.04, 26.98, 26.32, 26.14, 22.90, 22.88, 14.31. MALDI-TOF mass: calcd. for $C_{88}H_{120}NaO_{11}$ $[M + Na]^+$: m/z = 1375.87; found: 1375.82.

Compound 34. By a procedure similar to that for **3**, except that the reaction temperature was 40 °C, compound **34** was obtained in 93% yield (0.41 g, 0.29 mmol) from **31** (0.40 g, 0.31 mmol), 4-ethynylbenzoic acid (0.14 g, 1.00 mmol), DPTS (0.05 g, 0.16 mmol) and DIPC (0.10 g, 0.72 mmol). ^{1}H NMR (500 MHz, $CDCl_3$): δ (ppm) 8.07 (d, J = 8.6 Hz, 6H), 7.99 (d, J = 8.6 Hz, 2H), 7.61 (d, J = 8.6 Hz, 6H), 7.54 (d, J = 8.6 Hz, 8H), 6.97 (d, J = 8.6 Hz, 6H), 4.33 (t, J = 5.7 Hz, 6H), 4.23 (d, J = 5.7 Hz, 2H), 3.98 (t, J = 6.8 Hz, 6H), 3.22 (s, 1H), 1.79–1.73 (m, 10H), 1.46–1.28 (m, 63H), 0.88–0.86 (m, 6H). ^{13}C NMR (125 MHz, $CDCl_3$): δ (ppm) 166.54, 159.46, 145.06, 132.23, 132.03, 130.51, 130.03, 129.38, 128.51, 128.26, 126.62, 126.38, 126.35, 114.91, 79.94, 68.07, 67.91, 65.04, 64.92, 37.47, 31.90, 31.87, 31.78, 31.48, 29.98, 29.92, 29.53, 29.43, 29.41, 29.32, 29.28, 29.23, 28.76, 28.71, 28.63, 26.77, 26.01, 22.62, 14.07. MALDI-TOF mass: calcd. for $C_{94}H_{122}NaO_{11}$ $[M + Na]^+$: m/z = 1449.89; found: 1449.87.

Synthesis of 39 and 42

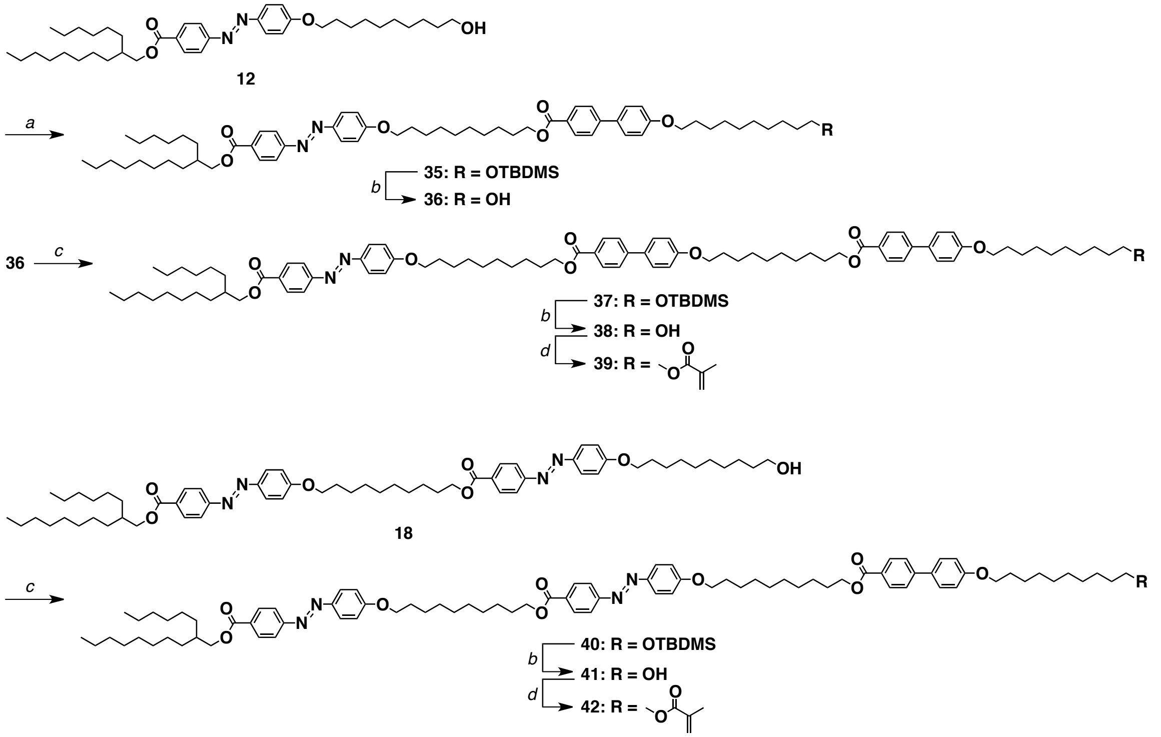

Reagents and conditions: (a) **23**, DPTS, DIPC, CH_2Cl_2, 25 °C; (b) TBAF, THF, 25 °C; (c) **23**, DPTS, DIPC, CH_2Cl_2, 40 °C; (d) methacryloyl chloride, triethylamine, CH_2Cl_2, 25 °C.

Compound 35. By a procedure similar to that for **3**, compound **35** was obtained in 84% yield (1.46 g, 1.34 mmol) from **12** (1.0 g, 1.60 mmol), **23** (0.78 g, 1.60 mmol), DPTS (0.10 g, 0.33 mmol) and DIPC (0.40 g, 3.20 mmol). ^{1}H NMR (500 MHz, $CDCl_3$): δ (ppm) 8.16 (d, J = 8.6 Hz, 2H), 8.08 (d, J = 8.6 Hz, 2H), 7.93 (d, J = 8.6 Hz, 2H), 7.90 (d, J = 8.6 Hz, 2H), 7.61 (d, J = 8.0 Hz, 2H), 7.55 (d, J = 8.6 Hz, 2H), 7.01 (d, J = 8.6 Hz, 2H), 6.98 (d, J = 8.6 Hz, 2H), 4.33 (t, J = 6.6 Hz, 2H), 4.25 (d, J = 5.7 Hz, 2H), 4.04 (t, J = 6.3 Hz, 2H), 4.00 (t, J = 6.8 Hz, 2H), 3.60 (t, J = 6.6 Hz, 2H), 1.84–1.75 (m, 7H), 1.55–1.30 (m, 50H), 0.89–0.86 (m, 15H), 0.05 (s, 6H). ^{13}C NMR (125 MHz, $CDCl_3$): δ (ppm) 166.67, 166.31, 162.35, 159.42, 155.36, 146.85, 145.20, 130.51, 130.06, 128.30, 126.41, 125.19, 122.34, 114.94, 114.80, 68.44, 68.13, 68.03, 65.05, 63.33, 37.48, 32.90, 31.92, 31.83, 31.48, 29.97, 29.64, 29.57, 29.52, 29.46, 29.44, 29.39, 29.34, 29.32, 29.25, 29.18, 28.76, 26.77, 26.05, 25.82, 22.68, 22.66, 18.39, 14.12, –5.25. MALDI-TOF mass: calcd. for $C_{68}H_{105}N_2O_7Si$ $[M + H]^+$: m/z = 1089.77; found: 1089.73.

Compound 36. By a procedure similar to that for **4**, compound **36** was obtained in 98% yield (1.22 g, 1.25 mmol) from **35** (1.40 g, 1.28 mmol) and TBAF (1.0 M in THF, 2.5 mL, 2.5 mmol). ^{1}H NMR (500 MHz, $CDCl_3$): δ (ppm) 8.16 (d, J = 8.6 Hz, 2H), 8.08 (d, J = 8.6 Hz, 2H), 7.93 (d,

J = 8.6 Hz, 2H), 7.90 (d, J = 8.6 Hz, 2H), 7.61 (d, J = 8.0 Hz, 2H), 7.55 (d, J = 8.6 Hz, 2H), 7.01 (d, J = 8.6 Hz, 2H), 6.97 (d, J = 8.6 Hz, 2H), 4.33 (t, J = 6.8 Hz, 2H), 4.25 (d, J = 5.8 Hz, 2H), 4.04 (t, J = 6.3 Hz, 2H), 4.00 (t, J = 6.3 Hz, 2H), 3.64 (t, J = 6.6 Hz, 2H), 1.85–1.71 (m, 7H), 1.61–1.54 (m, 2H), 1.46–1.29 (m, 49H), 0.88–0.86 (m, 6H). ^{13}C NMR (125 MHz, $CDCl_3$): δ (ppm) 166.67, 166.32, 162.32, 159.42, 155.37, 146.88, 145.20, 132.20, 131.58, 130.51, 130.06, 128.56, 128.30, 126.40, 125.20, 122.34, 114.94, 114.80, 114.20, 68.43, 68.12, 68.03, 65.05, 63.08, 37.48, 32.81, 31.92, 31.83, 31.48, 31.41, 29.97, 29.64, 29.57, 29.53, 29.49, 29.45, 29.44, 29.42, 29.37, 29.34, 29.32, 29.25, 29.18, 28.76, 26.79, 26.76, 26.04, 25.74, 22.68, 22.66, 14.11. MALDI-TOF mass: calcd. for $C_{62}H_{91}N_2O_7$ [M + H]$^+$: m/z = 975.68; found: 975.65.

Compound 37. By a procedure similar to that for **3**, except that the reaction temperature was 40 °C, compound **37** was obtained in 81% yield (1.20 g, 0.83 mmol) from **36** (1.0 g, 1.03 mmol), **23** (0.55 g, 1.10 mmol), DPTS (0.060 g, 0.21 mmol) and DIPC (0.25 g, 1.99 mmol). ^{1}H NMR (500 MHz, $CDCl_3$): δ (ppm) 8.16 (d, J = 8.6 Hz, 2H), 8.08 (d, J = 8.6 Hz, 4H), 7.94 (d, J = 8.6 Hz, 2H), 7.90 (d, J = 8.6 Hz, 2H), 7.61 (d, J = 8.6 Hz, 4H), 7.55 (d, J = 8.6 Hz, 2H), 7.55 (d, J = 8.6 Hz, 2H), 7.01 (d, J = 8.6 Hz, 2H), 6.98 (d, J = 8.6 Hz, 2H), 6.98 (d, J = 8.6 Hz, 2H), 4.33 (t, J = 6.6 Hz, 4H), 4.25 (d, J = 5.7 Hz, 2H), 4.04 (t, J = 6.6 Hz, 2H), 4.00 (t, J = 6.6 Hz, 4H), 3.60 (t, J = 6.6 Hz, 2H), 1.85–1.73 (m, 11H), 1.52–1.30 (m, 62H), 0.92–0.86 (m, 15H), 0.05 (s, 6H). ^{13}C NMR (125 MHz, $CDCl_3$): δ (ppm) 166.66, 166.23, 162.33, 159.45, 155.39, 145.22, 132.22, 131.51, 130.51, 130.06, 128.59, 128.31, 126.41, 125.19, 122.34, 114.97, 114.82, 68.45, 68.18, 68.04, 65.05, 63.33, 37.50, 32.90, 31.92, 31.83, 31.51, 29.97, 29.64, 29.57, 29.53, 29.44, 29.39, 29.36, 29.32, 29.27, 29.25, 29.19, 28.77, 26.80, 26.77, 26.05, 25.82, 22.68, 22.66, 18.39, 14.11, −5.24. MALDI-TOF mass: calcd. for $C_{91}H_{133}N_2O_{10}Si$ [M + H]$^+$: m/z = 1441.97; found: 1441.98.

Compound 38. By a procedure similar to that for **4**, compound **38** was obtained in 94% yield (1.24 g, 0.72 mmol) from **37** (1.10 g, 0.76 mmol) and TBAF (1.0 M in THF, 2.0 mL, 2.0 mmol). ^{1}H NMR (500 MHz, $CDCl_3$): δ (ppm) 8.16 (d, J = 8.6 Hz, 2H), 8.08 (d, J = 8.6 Hz, 4H), 7.94 (d, J = 8.6 Hz, 2H), 7.90 (d, J = 8.6 Hz, 2H), 7.61 (d, J = 8.0 Hz, 4H), 7.55 (d, J = 8.6 Hz, 2H), 7.55 (d, J = 8.6 Hz, 2H), 7.01 (d, J = 8.6 Hz, 2H), 6.97 (d, J = 8.6 Hz, 2H), 6.97 (d, J = 8.6 Hz, 2H), 4.34 (t, J = 6.6 Hz, 4H), 4.25 (d, J = 5.8 Hz, 2H), 4.04 (t, J = 6.3 Hz, 2H), 4.00 (t, J = 6.6 Hz, 4H), 3.64 (br, J = 6.6 Hz, 2H), 1.85–1.73 (m, 11H), 1.61–1.54 (m, 2H), 1.47–1.25 (m, 61H), 0.90–0.86 (m, 6H). ^{13}C NMR (125 MHz, $CDCl_3$): δ (ppm) 166.66, 166.21, 162.35, 159.44, 155.40, 145.21, 132.23, 130.51, 130.06, 128.58, 128.31, 126.40, 125.20, 122.34, 114.97, 114.82, 68.45, 68.15, 68.04, 65.06, 63.08, 37.50, 32.82, 31.92, 31.83, 31.50, 29.97, 29.64, 29.57, 29.53, 29.49, 29.46, 29.44, 29.41, 29.37, 29.32, 29.26, 29.18, 28.77, 26.80, 26.77, 26.05, 25.75, 22.68, 22.66, 14.11. MALDI-TOF mass: calcd. for $C_{85}H_{119}N_2O_{10}$ [M + H]$^+$: m/z = 1327.89; found: 1327.81.

Compound 39. By a procedure similar to that for **5**, compound **39** was obtained in 61% yield (0.51 g, 0.37 mmol) from **38** (0.80 g, 0.60 mmol), methacryloyl chloride (0.13 g, 1.20 mmol) and triethylamine (0.13 g, 1.21 mmol). ^{1}H NMR (500 MHz, $CDCl_3$): δ (ppm) 8.16 (d, J = 8.0 Hz, 2H), 8.07 (d, J = 8.6 Hz, 2H), 8.07 (d, J = 8.6 Hz, 2H), 7.93 (d, J = 8.6 Hz, 2H), 7.90 (d, J = 8.0 Hz, 2H), 7.61 (d, J = 8.6 Hz, 4H), 7.55 (d, J = 8.6 Hz, 2H), 7.55 (d, J = 8.6 Hz, 2H), 7.00 (d, J = 8.6 Hz, 2H), 6.98 (d, J = 8.6 Hz, 2H), 6.98 (d, J = 8.6 Hz, 2H), 6.09 (s, 1H), 5.54 (t, J = 1.7 Hz, 1H), 4.33 (t, J = 6.8 Hz, 4H), 4.25 (d, J = 5.7 Hz, 2H), 4.14 (t, J = 6.8 Hz, 2H), 4.05 (t, J = 6.8 Hz, 2H), 4.00 (t, J = 6.8 Hz, 2H), 4.00 (t, J = 6.8 Hz, 2H), 1.94 (s, 3H), 1.81–1.75 (m, 11H), 1.69–1.64 (m, 2H), 1.47–1.28 (m, 60H), 0.88–0.86 (m, 6H). ^{13}C NMR (125 MHz, $CDCl_3$): δ (ppm) 166.68, 166.22, 162.35, 159.45, 155.40, 145.21, 136.61, 132.23, 130.51, 130.06, 128.58, 128.33, 126.40, 125.22, 122.34, 114.97, 114.82, 68.45, 68.15, 68.04, 65.06, 63.11, 37.50, 32.83, 31.92, 31.85, 31.50, 29.98, 29.66, 29.57, 29.53, 29.49, 29.45, 29.41, 29.37, 29.32, 29.26, 29.18, 28.77, 26.81, 26.77, 26.05, 25.74, 22.68, 22.66, 14.13. MALDI-TOF mass: calcd. for $C_{89}H_{123}N_2O_{11}$ $[M + H]^+$: m/z = 1395.91; found: 1395.94.

Compound 40. By a procedure similar to that for **3**, except that the reaction temperature was 40 °C, compound **40** was obtained in 94% yield (1.38 g, 0.94 mmol) from **18** (1.0 g, 1.0 mmol), **23** (0.53 g, 1.10 mmol), DPTS (0.06 g, 0.21 mmol) and DIPC (0.26 g, 2.00 mmol). ^{1}H NMR (500 MHz, $CDCl_3$): δ (ppm) 8.17 (d, J = 8.6 Hz, 2H), 8.16 (d, J = 8.6 Hz, 2H), 8.08 (d, J = 8.0 Hz, 2H), 7.93 (d, J = 8.6 Hz, 2H), 7.93 (d, J = 8.6 Hz, 2H), 7.90 (d, J = 8.6 Hz, 2H), 7.90 (d, J = 8.6 Hz, 2H), 7.61 (d, J = 8.6 Hz, 2H), 7.55 (d, J = 8.6 Hz, 2H), 7.00 (d, J = 8.6 Hz, 2H), 7.00 (d, J = 8.6 Hz, 2H), 6.98 (d, J = 8.6 Hz, 2H), 4.35 (t, J = 6.9 Hz, 2H), 4.33 (t, J = 6.6 Hz, 2H), 4.25 (d, J = 5.7 Hz, 2H), 4.04 (t, J = 6.6 Hz, 2H), 4.04 (t, J = 6.6 Hz, 2H), 4.00 (t, J = 6.6 Hz, 2H), 3.60 (t, J = 6.6 Hz, 2H), 1.83–1.76 (m, 11H), 1.50–1.25 (m, 62H), 0.90–0.86 (m, 15H), 0.05 (s, 6H). ^{13}C NMR (125 MHz, $CDCl_3$): δ (ppm) 166.30, 166.21, 162.34, 159.47, 155.39, 145.23, 130.53, 130.51, 130.06, 128.30, 126.41, 125.19, 122.34, 114.97, 114.82, 68.49, 68.17, 65.39, 65.05, 63.33, 37.53, 32.90, 31.88, 31.51, 29.57, 29.44, 29.34, 29.27, 29.24, 29.19, 28.74, 26.84, 26.06, 26.04, 26.00, 22.68, 22.65, 18.41, 14.11, −5.24. MALDI-TOF mass: calcd. for $C_{91}H_{133}N_4O_{10}Si$ $[M + H]^+$: m/z = 1469.98; found: 1469.88.

Compound 41. By a procedure similar to that for **4**, compound **41** was obtained in 92% yield (1.10 g, 0.81 mmol) from **40** (1.30 g, 0.88 mmol) and TBAF (1.0 M in THF, 2.0 mL, 2.0 mmol). ^{1}H NMR (500 MHz, $CDCl_3$): δ (ppm) 8.17 (d, J = 8.6 Hz, 2H), 8.16 (d, J = 8.6 Hz, 2H), 8.08 (d, J = 8.6 Hz, 2H), 7.93 (d, J = 8.6 Hz, 2H), 7.93 (d, J = 8.6 Hz, 2H), 7.90 (d, J = 8.6 Hz, 2H), 7.90 (d, J = 8.6 Hz, 2H), 7.61 (d, J = 8.6 Hz, 2H), 7.55 (d, J = 8.6 Hz, 2H), 7.01 (d, J = 8.6 Hz, 2H), 7.01 (d, J = 8.6 Hz, 2H), 6.98 (d, J = 8.6 Hz, 2H), 4.35 (t, J = 6.9 Hz, 2H), 4.33 (t, J = 6.6 Hz, 2H), 4.25 (d, J = 5.8 Hz, 2H), 4.04 (t, J = 6.6 Hz, 2H), 4.04 (t, J = 6.6 Hz, 2H), 4.00 (t, J = 6.6 Hz,

2H), 3.64 (q, J = 5.8 Hz, 2H), 1.83–1.76 (m, 11H), 1.49–1.46 (m, 2H), 1.45–1.25 (m, 61H), 0.89–0.86 (m, 6H). ^{13}C NMR (125 MHz, $CDCl_3$): δ (ppm) 166.32, 166.21, 162.37, 159.40, 155.46, 145.21, 132.22, 130.51, 130.06, 128.31, 126.41, 125.19, 122.34, 114.97, 114.82, 68.45, 68.15, 63.09, 37.50, 32.86, 32.81, 31.92, 31.86, 31.50, 31.43, 29.97, 29.53, 29.49, 29.44, 29.41, 29.37, 29.34, 29.31, 29.26, 29.24, 29.18, 26.77, 26.04, 26.01, 25.75, 22.68, 14.10. MALDI-TOF mass: calcd. for $C_{85}H_{119}N_4O_{10}$ $[M + H]^+$: m/z = 1355.89; found: 1355.84.

Compound 42. By a procedure similar to that for **5**, compound **42** was obtained in 66% yield (0.55 g, 0.39 mmol) from **41** (0.80 g, 0.59 mmol), methacryloyl chloride (0.13 g, 1.22 mmol) and triethylamine (0.13 g, 1.21 mmol). ^{1}H NMR (500 MHz, $CDCl_3$): δ (ppm) 8.17 (d, J = 8.6 Hz, 2H), 8.16 (d, J = 8.6 Hz, 2H), 8.07 (d, J = 8.6 Hz, 2H), 7.93 (d, J = 8.6 Hz, 2H), 7.93 (d, J = 8.6 Hz, 2H), 7.91 (d, J = 8.6 Hz, 2H), 7.91 (d, J = 8.6 Hz, 2H), 7.61 (d, J = 8.6 Hz, 2H), 7.55 (d, J = 8.6 Hz, 2H), 7.00 (d, J = 8.6 Hz, 2H), 7.00 (d, J = 8.6 Hz, 2H), 6.98 (d, J = 8.6 Hz, 2H), 6.10 (s, 1H), 5.54 (t, J = 1.7 Hz, 1H), 4.33 (t, J = 6.7 Hz, 2H), 4.33 (t, J = 6.7 Hz, 2H), 4.25 (d, J = 5.7 Hz, 2H), 4.14 (t, J = 6.8 Hz, 2H), 4.05 (t, J = 6.8 Hz, 2H), 4.05 (t, J = 6.8 Hz, 2H), 4.00 (t, J = 6.8 Hz, 2H), 1.94 (s, 3H), 1.82–1.75 (m, 11H), 1.69–1.60 (m, 2H), 1.48–1.27 (m, 60H), 0.89–0.86 (m, 6H). ^{13}C NMR (125 MHz, $CDCl_3$): δ (ppm) 166.68, 166.22, 162.37, 159.47, 155.41, 145.21, 136.58, 132.23, 130.52, 130.07, 128.58, 128.35, 126.40, 125.23, 122.34, 114.98, 114.82, 68.45, 68.17, 68.04, 65.06, 63.11, 37.52, 32.83, 31.92, 31.85, 31.51, 29.98, 29.66, 29.57, 29.53, 29.49, 29.45, 29.41, 29.37, 29.34, 29.26, 29.18, 28.78, 26.81, 26.77, 26.07, 25.75, 22.68, 22.66, 14.12. MALDI-TOF mass: calcd. for $C_{89}H_{123}N_4O_{11}$ $[M + H]^+$: m/z = 1423.92; found: 1423.91.

Synthesis of 51

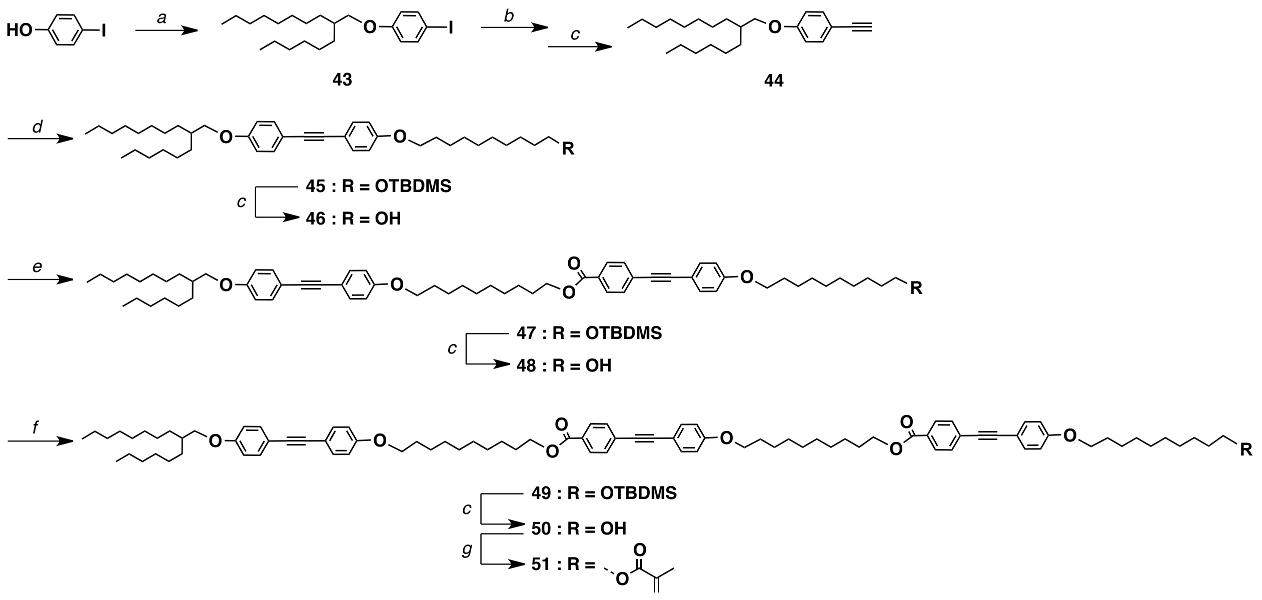

Reagents and conditions: (a) 1-bromo-2-hexyldecane, Cs_2CO_3, DMF, 70 °C; (b) Trimethylsilylacetylene, CuI, triethylamine, tetrakis(triphenylphosphine)palladium, THF, reflux; (c) TBAF, THF, 25 °C; (d) **1**, CuI, triethylamine, tetrakis(triphenylphosphine)palladium, THF, reflux; (e) **2**, DPTS, DIPC, CH_2Cl_2, 25 °C; (f) **2**, DPTS, DIPC, CH_2Cl_2, 40 °C; (g) methacryloyl chloride, triethylamine, CH_2Cl_2, 25 °C.

Compound 43. By a procedure similar to that for **1**, compound **43** was obtained in 72% yield (3.2 g, 7.2 mmol) from 4-iodophenol (2.2 g, 10.0 mmol), 1-bromo-2-hexyldecane (3.1 g, 10.0 mmol) and Cs_2CO_3 (3.6 g, 11.2 mmol). ^{1}H NMR (500 MHz, $CDCl_3$): δ (ppm) 7.51 (d, J = 9.2 Hz, 2H), 6.65 (d, J = 9.2 Hz, 2H), 3.78 (d, J = 5.8 Hz, 2H), 1.75 (m, 1H), 1.43–1.27 (m, 24H), 0.88 (m, 6H). ^{13}C NMR (125 MHz, $CDCl_3$): δ (ppm) 158.51, 132.12, 116.32, 112.43, 71.17, 37.88, 31.89, 31.31, 31.29, 29.99, 29.66, 29.57, 29.31, 26.80, 26.78, 22.67, 14.09. MALDI-TOF mass: calcd. for $C_{22}H_{38}IO$ $[M + H]^+$: m/z = 444.43; found: 444.40.

Compound 44. To a THF solution (30 mL) of **43** (3.0 g, 6.8 mmol) were successively added trimethylsilylacetylene (2 mL, 14.5 mmol), CuI (0.051 g, 0.3 mmol), triethylamine (30 mL) and tetrakis(triphenylphosphine)palladium (0.616 g, 0.5 mmol), and the mixture was purged with N_2 for 20 min and then refluxed for 18 h under N_2. The reaction mixture was poured into a saturated aqueous solution of NH_4Cl and extracted with AcOEt, CH_2Cl_2 and ether. The combined organic extract was evaporated to dryness under a reduced pressure, and then TBAF (1.0 M in THF, 6.0 mL, 6.0 mmol) was added to a THF solution (20 mL) of the above crude product, and the mixture was stirred at 25 °C for 12 h under Ar. The reaction mixture was evaporated to dryness under a reduced pressure, and a $CHCl_3$ solution of the residue was washed with a saturated aqueous

solution of NH_4Cl. An organic phase separated was dried over anhydrous $MgSO_4$ and evaporated to dryness under a reduced pressure. The residue was subjected to column chromatography (SiO_2, *n*-hexane) to allow isolation of **44** as colorless oil (1.5 g, 4.4 mmol) in 65% yield. ^{1}H NMR (500 MHz, $CDCl_3$): δ (ppm) 7.41 (d, J = 9.2 Hz, 2H), 6.83 (d, J = 9.2 Hz, 2H), 3.82 (d, J = 5.7 Hz, 2H), 2.99 (s, 1H), 1.76 (m, 1H), 1.47–1.27 (br, 22H), 0.88 (m, 6H). ^{13}C NMR (125 MHz, $CDCl_3$): δ (ppm) 159.79, 133.51, 132.12, 116.32, 114.49, 113.74, 112.43, 83.81, 75.57, 71.17, 70.97, 37.86, 31.88, 31.83, 31.30, 29.99, 29.65, 29.56, 29.31, 26.81, 26.78, 22.67, 14.09. MALDI-TOF mass: calcd. for $C_{24}H_{39}O$ $[M + H]^+$: m/z = 342.56; found: 342.52.

Compound 45. By a procedure similar to that for **2**, compound **45** was obtained in 73% yield (2.3 g, 3.2 mmol) from **44** (1.5 g, 4.4 mmol), **1** (2.2 g, 4.4 mmol), CuI (0.051 g, 0.3 mmol), tetrakis(triphenylphosphine)palladium (0.616 g, 0.5 mmol) and triethylamine (30 mL). ^{1}H NMR (500 MHz, $CDCl_3$): δ (ppm) 7.42 (d, J = 8.6 Hz, 4H), 6.84 (d, J = 8.6 Hz, 4H), 3.96 (t, J = 6.6 Hz, 2H), 3.83 (d, J = 5.8 Hz, 2H), 3.60 (t, J = 6.6 Hz, 2H), 1.77 (m, 4H), 1.52–1.27 (br, 37H), 0.89–0.86 (m, 15H), 0.05 (s, 6H). ^{13}C NMR (125 MHz, $CDCl_3$): δ (ppm) 159.51, 136.23, 133.26, 132.78, 132.74, 132.21, 131.33, 129.78, 114.77, 114.73, 71.02, 68.10, 63.32, 38.03, 32.89, 31.91, 31.87, 31.76, 31.40, 30.03, 29.80, 29.72, 29.58, 29.51, 29.49, 29.47, 29.44, 29.42, 29.36, 29.33, 29.26, 26.87, 26.83, 26.46, 26.43, 26.07, 25.98, 25.80, 22.67, 22.62, 22.59, 14.10, −5.26. MALDI-TOF mass: calcd. for $C_{46}H_{77}O_3Si$ $[M + H]^+$: m/z = 705.18; found: 705.20.

Compound 46. By a procedure similar to that for **4**, compound **46** was obtained in 89% yield (1.10 g, 1.9 mmol) from **45** (1.5 g, 2.1 mmol) and TBAF (1.0 M in THF, 3.0 mL, 3.0 mmol). ^{1}H NMR (500 MHz, $CDCl_3$): δ (ppm) 7.42 (d, J = 8.6 Hz, 4H), 6.85 (d, J = 8.6 Hz, 4H), 3.96 (t, J = 6.6 Hz, 2H), 3.83 (d, J = 5.8 Hz, 2H), 3.64 (br, 2H), 1.77 (m, 4H), 1.58–1.55 (br, 2H), 1.52–1.20 (br, 36H), 0.89–0.86 (m, 6H). ^{13}C NMR (125 MHz, $CDCl_3$): δ (ppm) 159.19, 136.34, 133.39, 132.23, 132.11, 129.67, 114.76, 114.73, 71.01, 68.06, 63.10, 37.99, 32.79, 31.84, 31.82, 31.47, 30.01, 29.69, 29.58, 29.53, 29.47, 29.36, 29.33, 29.30, 26.82, 26.78, 26.04, 25.67, 25.61, 22.68, 14.09. MALDI-TOF mass: calcd. for $C_{40}H_{63}O_3$ $[M + H]^+$: m/z = 590.92; found: 590.90.

Compound 47. By a procedure similar to that for **3**, compound **49** was obtained in 80% yield (0.80 g, 0.74 mmol) from **46** (0.55 g, 0.93 mmol), **2** (0.5 g, 1.0 mmol), DPTS (0.061 g, 0.2 mmol) and DIPC (0.144 g, 1.2 mmol). ^{1}H NMR (500 MHz, $CDCl_3$): δ (ppm) 8.00 (d, J = 8.0 Hz, 2H), 7.55 (d, J = 8.6 Hz, 2H), 7.46 (d, J = 8.6 Hz, 2H), 7.42 (d, J = 8.6 Hz, 4H), 6.87 (d, J = 8.6 Hz, 2H), 6.85 (d, J = 8.6 Hz, 4H), 4.32 (t, J = 6.6 Hz, 2H), 3.96 (t, J = 6.6 Hz, 2H), 3.96 (t, J = 6.6 Hz, 2H), 3.83 (d, J = 5.8 Hz, 2H), 3.60 (t, J = 6.6 Hz, 2H), 1.78 (m, 7H), 1.55–1.22 (br, 50H), 0.89–0.87 (m, 15H), 0.05 (s, 6H). ^{13}C NMR (125 MHz, $CDCl_3$): δ (ppm) 167.53, 166.20, 159.59, 136.51, 133.21, 132.81, 132.77, 131.23, 129.44, 114.60, 114.51, 114.47, 92.61, 87.46, 70.98, 68.09, 68.01, 65.26, 63.31, 37.90, 32.88, 31.89, 31.84, 31.34, 31.32, 30.00, 29.67, 29.55, 29.49,

29.44, 29.41, 29.36, 29.32, 29.22, 29.19, 29.17, 28.69, 26.82, 26.80, 26.00, 25.98, 25.79, 22.67, 18.38, 14.10, −5.26. MALDI-TOF mass: calcd. for $C_{71}H_{105}O_6Si$ $[M + H]^+$: *m/z* = 1081.67; found: 1081.65.

Compound 48. By a procedure similar to that for **4**, compound **48** was obtained in 85% yield (0.55 g, 0.55 mmol) from **47** (0.70 g, 0.65 mmol) and TBAF (1.0 M in THF, 1 mL, 1 mmol). ^1H NMR (500 MHz, $CDCl_3$): δ (ppm) 8.00 (d, *J* = 8.0 Hz, 2H), 7.55 (d, *J* = 8.6 Hz, 2H), 7.46 (d, *J* = 8.6 Hz, 2H), 7.42 (d, *J* = 8.6 Hz, 4H), 6.87 (d, *J* = 8.6 Hz, 2H), 6.85 (d, *J* = 8.6 Hz, 4H), 4.32 (t, *J* = 6.6 Hz, 2H), 3.96 (t, *J* = 6.6 Hz, 2H), 3.96 (t, *J* = 6.6 Hz, 2H), 3.83 (d, *J* = 5.8 Hz, 2H), 3.64 (m, 2H), 1.78 (m, 7H), 1.59–1.56 (m, 2H), 1.45–1.21 (m, 49H), 0.90–0.87 (m, 6H). ^{13}C NMR (125 MHz, $CDCl_3$): δ (ppm) 166.78, 159.21, 158.92, 133.22, 132.81, 132.77, 131.23, 129.44, 128.31, 115.51, 115.37, 114.60, 114.52, 114.48, 87.99, 87.89, 87.47, 70.98, 68.08, 68.02, 65.24, 63.07, 37.90, 32.79, 31.89, 31.84, 31.34, 31.32, 30.00, 29.67, 29.57, 29.50, 29.46, 29.44, 29.41, 29.38, 29.34, 29.32, 29.22, 29.19, 29.16, 28.69, 26.82, 26.80, 26.00, 25.72, 22.67, 14.10. MALDI-TOF mass: calcd. for $C_{65}H_{91}O_6$ $[M + H]^+$: *m/z* = 997.41; found: 997.38.

Compound 49. By a procedure similar to that for **3**, except that the reaction temperature was 40 °C, compound **49** was obtained in 92% yield (0.71 g, 0.49 mmol) from **48** (0.50 g, 0.50 mmol), **2** (0.30 g, 0.59 mmol), DPTS (0.060 g, 0.21 mmol) and DIPC (0.12 g, 1.00 mmol). ^1H NMR (500 MHz, $CDCl_3$): δ (ppm) 8.00 (d, *J* = 8.0 Hz, 4H), 7.55 (d, *J* = 8.6 Hz, 4H), 7.46 (d, *J* = 8.6 Hz, 4H), 7.42 (d, *J* = 8.6 Hz, 4H), 6.87 (d, *J* = 8.6 Hz, 4H), 6.85 (d, *J* = 8.6 Hz, 4H), 4.32 (t, *J* = 6.6 Hz, 4H), 3.96 (t, *J* = 6.6 Hz, 6H), 3.83 (d, *J* = 5.8 Hz, 2H), 3.60 (t, *J* = 6.6 Hz, 2H), 1.82–1.75 (m, 11H), 1.56–1.21 (m, 62H), 0.89–0.87 (m, 15H), 0.05 (s, 6H). ^{13}C NMR (125 MHz, $CDCl_3$): δ (ppm) 167.55, 166.21, 159.59, 136.50, 133.23, 132.81, 131.21, 131.19, 129.43, 114.60, 114.48, 114.47, 92.62, 87.46, 70.94, 68.92, 68.11, 65.26, 63.31, 37.91, 32.88, 31.89, 31.85, 31.34, 31.32, 30.01, 29.67, 29.55, 29.50, 29.43, 29.40, 29.36, 29.32, 29.23, 29.19, 29.15, 28.62, 26.82, 26.80, 26.02, 25.98, 25.79, 22.67, 18.37, 14.10, −5.26. MALDI-TOF mass: calcd. for $C_{96}H_{133}O_9Si$ $[M + H]^+$: *m/z* = 1458.16; found: 1458.13.

Compound 50. By a procedure similar to that for **4**, compound **50** was obtained in 91% yield (0.60 g, 0.45 mmol) from **49** (0.70 g, 0.49 mmol) and TBAF (1.0 M in THF, 1 mL, 1 mmol). ^1H NMR (500 MHz, $CDCl_3$): δ (ppm) 8.00 (d, *J* = 8.0 Hz, 4H), 7.55 (d, *J* = 8.6 Hz, 4H), 7.46 (d, *J* = 8.6 Hz, 4H), 7.42 (d, *J* = 8.6 Hz, 4H), 6.87 (d, *J* = 8.6 Hz, 4H), 6.85 (d, *J* = 8.6 Hz, 4H), 4.32 (t, *J* = 6.6 Hz, 4H), 3.96 (t, *J* = 6.6 Hz, 6H), 3.83 (d, *J* = 5.8 Hz, 2H), 3.64 (m, 2H), 1.83–1.75 (m, 11H), 1.57–1.52 (m, 2H), 1.48–1.21 (m, 61H), 0.89–0.87 (m, 6H). ^{13}C NMR (125 MHz, $CDCl_3$): δ (ppm) 166.76, 159.21, 158.92, 133.21, 132.84, 132.73, 131.21, 129.44, 128.31, 115.51, 115.35, 114.60, 114.52, 114.46, 87.99, 87.85, 70.98, 68.09, 68.02, 65.26, 63.10, 37.92, 32.79, 31.86, 31.84, 31.32, 31.28, 30.01, 29.67, 29.57, 29.50, 29.44, 29.43, 29.39, 29.36, 29.34, 29.32, 29.22,

29.19, 29.15, 28.69, 26.82, 26.78, 26.00, 25.72, 22.65, 14.10. MALDI-TOF mass: calcd. for $C_{90}H_{119}O_9$ $[M + H]^+$: *m/z* = 1344.89; found: 1344.85.

Compound 51. By a procedure similar to that for **5**, compound **51** was obtained in 86% yield (0.45 g, 0.32 mmol) from **50** (0.50 g, 0.37 mmol), methacryloyl chloride (0.26 mL, 2.7 mmol) and triethylamine (0.38 mL, 2.7 mmol). ^1H NMR (500 MHz, $CDCl_3$): δ (ppm) 8.00 (d, J = 8.0 Hz, 4H), 7.55 (d, J = 8.6 Hz, 4H), 7.46 (d, J = 8.6 Hz, 4H), 7.42 (d, J = 8.6 Hz, 4H), 6.87 (d, J = 8.6 Hz, 2H), 6.84 (d, J = 8.6 Hz, 4H), 6.10 (s, 1H), 5.54 (t, J = 1.7 Hz, 1H), 4.32 (t, J = 6.6 Hz, 4H), 4.14 (t, J = 6.6 Hz, 2H), 3.96 (t, J = 6.6 Hz, 6H), 3.83 (d, J = 5.8 Hz, 2H), 1.94 (s, 3H), 1.80–1.76 (m, 11H), 1.70–1.54 (m, 2H), 1.45–1.27 (m, 60H), 0.89–0.86 (m, 6H). ^{13}C NMR (125 MHz, $CDCl_3$): δ (ppm) 167.54, 166.18, 159.59, 136.55, 133.21, 132.80, 132.76, 131.23, 129.43, 128.32, 125.12, 114.59, 114.51, 114.47, 92.61, 87.46, 70.97, 68.06, 68.01, 65.23, 64.79, 37.89, 31.88, 31.83, 31.33, 29.99, 29.66, 29.57, 29.43, 29.40, 29.32, 29.21, 29.16, 28.68, 28.58, 26.82, 26.79, 25.99, 25.94, 22.66, 18.32, 14.09. MALDI-TOF mass: calcd. for $C_{94}H_{123}O_{10}$ $[M + H]^+$: *m/z* = 1411.97; found: 1411.99.

Synthesis of 59

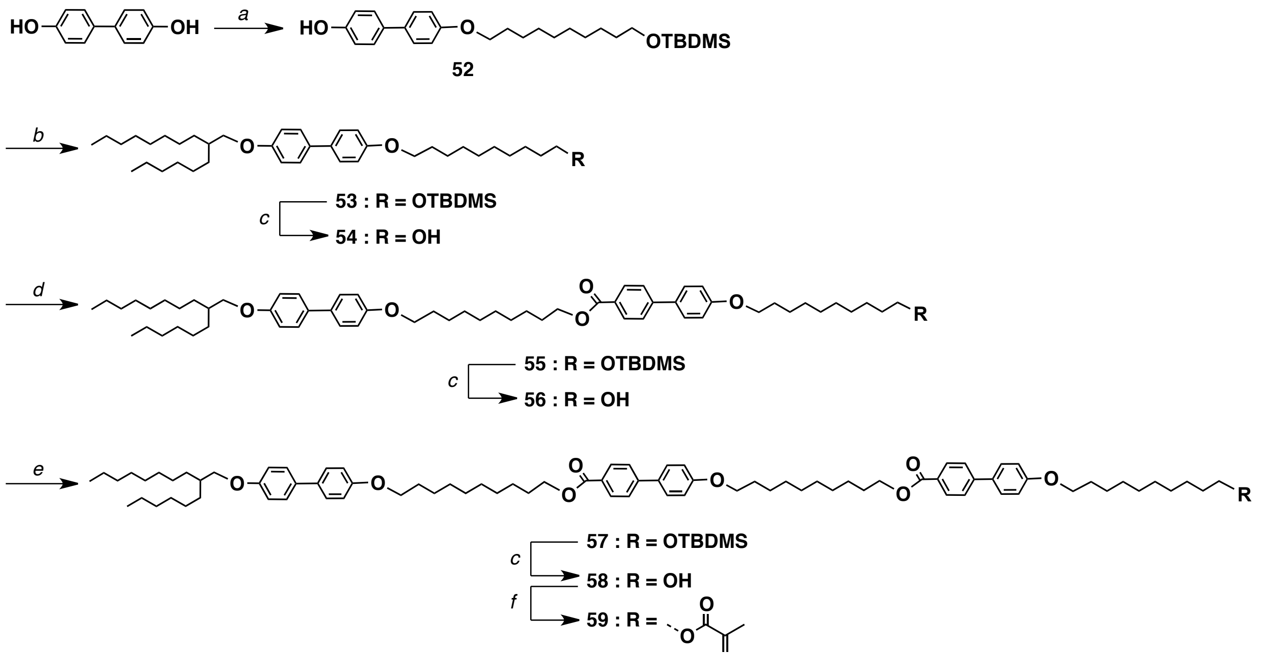

Reagents and conditions: (a) 10-bromodecyl *tert*-butyldimethylsilyl ether, Cs_2CO_3, DMF, 70 °C; (b) 1-bromo-2-hexyldecane, Cs_2CO_3, DMF, 70 °C; (c) TBAF, THF, 25 °C; (d) **23**, DPTS, DIPC, CH_2Cl_2, 25 °C; (e) **23**, DPTS, DIPC, CH_2Cl_2, 40 °C; (f) methacryloyl chloride, triethylamine, CH_2Cl_2, 25 °C.

Compound 52. By a procedure similar to that for **1**, compound **52** was obtained in 40% yield (1.8 g, 3.9 mmol) from 4,4'-biphenol (4.0 g, 21.0 mmol), 10-bromodecyl *tert*-butyldimethylsilyl ether (3.5 g, 10.0 mmol) and Cs_2CO_3 (7.3 g, 22.5 mmol). ^{1}H NMR (500 MHz, $CDCl_3$): δ (ppm) 7.43 (d x 2, J = 8.6 Hz, 4H), 6.93 (d, J = 8.6 Hz, 2H), 6.87 (d, J = 8.6 Hz, 2H), 4.99 (s, 1H), 3.98 (t, J = 6.6 Hz, 2H), 3.61 (t, J = 6.9 Hz, 2H), 1.77 (m, 2H), 1.53–1.43 (m, 4H), 1.34–1.23 (br, 12H), 0.89 (s, 9H), 0.05 (s, 6H). ^{13}C NMR (125 MHz, $CDCl_3$): δ (ppm) 158.25, 154.59, 133.71, 133.19, 127.91, 127.65, 115.56, 114.74, 68.10, 63.40, 32.84, 29.56, 29.51, 29.41, 29.38, 29.29, 26.05, 25.99, 25.78, 18.40, –5.24. MALDI-TOF mass: calcd. for $C_{28}H_{45}O_3Si$ $[M + H]^+$: m/z = 456.73; found: 456.73.

Compound 53. By a procedure similar to that for **1**, compound **53** was obtained in 61% yield (0.62 g, 0.91 mmol) from **52** (0.70 g, 1.5 mmol), 1-bromo-2-hexyldecane (0.62 g, 2.0 mmol) and Cs_2CO_3 (0.72 g, 2.2 mmol). ^{1}H NMR (500 MHz, $CDCl_3$): δ (ppm) 7.45 (d, J = 8.6 Hz, 4H), 6.93 (d, J = 8.6 Hz, 4H), 3.98 (t, J = 6.6 Hz, 2H), 3.85 (d, J = 5.8 Hz, 2H), 3.60 (t, J = 6.6 Hz, 2H), 1.81–1.76 (m, 4H), 1.59–1.14 (br, 37H), 0.89–0.86 (m, 15H), 0.05 (s, 6H). ^{13}C NMR (125 MHz, $CDCl_3$): δ (ppm) 158.51, 158.22, 133.39, 133.26, 133.22, 127.93, 127.65, 127.61, 114.77, 114.74, 71.02, 68.09, 63.32, 38.00, 32.89, 31.91, 31.87, 31.73, 31.40, 30.03, 29.79, 29.70, 29.59, 29.57,

29.52, 29.48, 29.44, 29.42, 29.39, 29.33, 29.26, 26.85, 26.83, 26.46, 26.41, 26.07, 25.99, 25.80, 22.67, 22.65, 22.60, 14.09, 14.01, −5.26. MALDI-TOF mass: calcd. for $C_{44}H_{77}O_3Si$ $[M + H]^+$: m/z = 681.16; found: 681.13.

Compound 54. By a procedure similar to that for **4**, compound **54** was obtained in 90% yield (0.45 g, 0.79 mmol) from **53** (0.60 g, 0.88 mmol) and TBAF (1.0 M in THF, 1 mL, 1 mmol). ^1H NMR (500 MHz, $CDCl_3$): δ (ppm) 7.45 (d, J = 8.6 Hz, 4H), 6.94 (d, J = 8.6 Hz, 4H), 3.98 (t, J = 6.6 Hz, 2H), 3.85 (d, J = 5.8 Hz, 2H), 3.65 (br, 2H), 1.81–1.76 (m, 4H), 1.59–1.14 (br, 38H), 0.89–0.86 (m, 6H). ^{13}C NMR (125 MHz, $CDCl_3$): δ (ppm) 158.51, 158.19, 133.39, 133.23, 127.63, 127.59, 114.76, 114.73, 71.01, 68.06, 63.06, 37.98, 32.79, 31.89, 31.85, 31.40, 30.01, 29.68, 29.58, 29.51, 29.47, 29.38, 29.35, 29.30, 26.84, 26.81, 26.04, 25.71, 25.63, 22.66, 14.09. MALDI-TOF mass: calcd. for $C_{38}H_{63}O_3$ $[M + H]^+$: m/z = 566.90; found: 566.92.

Compound 55. By a procedure similar to that for **3**, compound **55** was obtained in 87% yield (0.71 g, 0.69 mmol) from **54** (0.45 g, 0.79 mmol), **23** (0.40 g, 0.83 mmol), DPTS (0.031 g, 0.1 mmol) and DIPC (0.144 g, 1.2 mmol). ^1H NMR (500 MHz, $CDCl_3$): δ (ppm) 8.07 (d, J = 8.0 Hz, 2H), 7.61 (d, J = 8.6 Hz, 2H), 7.55 (d, J = 8.6 Hz, 2H), 7.45 (d, J = 8.6 Hz, 4H), 6.97 (d, J = 8.6 Hz, 2H), 6.94 (d, J = 8.6 Hz, 4H), 4.33 (t, J = 6.6 Hz, 2H), 3.97 (t, J = 6.6 Hz, 2H), 3.97 (t, J = 6.6 Hz, 2H), 3.85 (d, J = 5.8 Hz, 2H), 3.60 (t, J = 6.6 Hz, 2H), 1.78 (m, 7H), 1.55–1.25 (br, 50H), 0.89–0.86 (m, 15H), 0.05 (s, 6H). ^{13}C NMR (125 MHz, $CDCl_3$): δ (ppm) 166.67, 159.40, 158.68, 158.32, 145.19, 143.92, 130.04, 128.30, 127.61, 126.39, 114.93, 114.77, 114.74, 71.03, 68.16, 68.04, 65.06, 63.32, 38.01, 32.89, 31.90, 31.86, 31.44, 29.98, 29.69, 29.59, 29.56, 29.51, 29.43, 29.38, 29.36, 29.32, 28.77, 26.85, 26.77, 26.04, 25.98, 25.80, 25.79, 22.67, 14.22, 14.09, −5.26. MALDI-TOF mass: calcd. for $C_{67}H_{105}O_6Si$ $[M + H]^+$: m/z = 1033.62; found: 1033.65.

Compound 56. By a procedure similar to that for **4**, compound **56** was obtained in 94% yield (0.60 g, 0.65 mmol) from **55** (0.70 g, 0.69 mmol) and TBAF (1.0 M in THF, 1 mL, 1 mmol). ^1H NMR (500 MHz, $CDCl_3$): δ (ppm) 8.07 (d, J = 8.0 Hz, 2H), 7.61 (d, J = 8.6 Hz, 2H), 7.55 (d, J = 8.6 Hz, 2H), 7.45 (d, J = 8.6 Hz, 4H), 6.97 (d, J = 8.6 Hz, 2H), 6.94 (d, J = 8.6 Hz, 4H), 4.33 (t, J = 6.6 Hz, 2H), 3.97 (t, J = 6.6 Hz, 2H), 3.97 (t, J = 6.6 Hz, 2H), 3.85 (d, J = 5.8 Hz, 2H), 3.64 (br, 2H), 1.78 (m, 7H), 1.59–1.55 (m, 2H), 1.52–1.28 (m, 49H), 0.89–0.86 (m, 6H). ^{13}C NMR (125 MHz, $CDCl_3$): δ (ppm) 166.68, 159.43, 158.68, 158.35, 145.19, 143.92, 130.04, 128.34, 127.62, 126.39, 114.91, 114.77, 71.05, 68.16, 68.10, 65.06, 63.22, 38.00, 32.82, 31.90, 31.86, 31.46, 29.98, 29.69, 29.59, 29.57, 29.51, 29.43, 29.40, 29.36, 29.32, 28.73, 26.85, 26.75, 26.04, 25.94, 25.80, 25.73, 22.67, 14.09. MALDI-TOF mass: calcd. for $C_{61}H_{91}O_6$ $[M + H]^+$: m/z = 919.36; found: 919.34.

Compound 57. By a procedure similar to that for **3**, except that the reaction temperature was 40 °C, compound **57** was obtained in 82% yield (0.68 g, 0.49 mmol) from **56** (0.55 g, 0.60 mmol), **23** (0.35 g, 0.70 mmol), DPTS (0.060 g, 0.21 mmol) and DIPC (0.12 g, 1.00 mmol). ^{1}H NMR (500 MHz, $CDCl_3$): δ (ppm) 8.07 (d, J = 8.0 Hz, 4H), 7.61 (d, J = 8.6 Hz, 4H), 7.55 (d, J = 8.6 Hz, 4H), 7.45 (d, J = 8.6 Hz, 4H), 6.97 (d, J = 8.6 Hz, 4H), 6.94 (d, J = 8.6 Hz, 4H), 4.33 (t, J = 6.6 Hz, 4H), 3.97 (t, J = 6.6 Hz, 6H), 3.85 (d, J = 5.8 Hz, 2H), 3.60 (t, J = 6.6 Hz, 2H), 1.83–1.75 (m, 11H), 1.56–1.28 (m, 62H), 0.89–0.86 (m, 15H), 0.05 (s, 6H). ^{13}C NMR (125 MHz, $CDCl_3$): δ (ppm) 166.67, 159.40, 158.70, 158.32, 145.19, 143.91, 130.04, 128.30, 127.61, 126.39, 114.95, 114.77, 114.71, 71.03, 68.16, 68.04, 65.08, 63.35, 38.22, 32.82, 31.90, 31.86, 31.44, 29.98, 29.65, 29.59, 29.54, 29.51, 29.48, 29.38, 29.36, 29.31, 28.76, 26.85, 26.77, 26.01, 25.98, 25.84, 25.79, 22.67, 14.21, 14.09, −5.26. MALDI-TOF mass: calcd. for $C_{90}H_{133}O_9Si$ $[M + H]^+$: m/z = 1386.09; found: 1386.11.

Compound 58. By a procedure similar to that for **4**, compound **58** was obtained in 91% yield (0.55 g, 0.45 mmol) from **57** (0.65 g, 0.47 mmol) and TBAF (1.0 M in THF, 1 mL, 1 mmol). ^{1}H NMR (500 MHz, $CDCl_3$): δ (ppm) 8.07 (d, J = 8.0 Hz, 4H), 7.61 (d, J = 8.6 Hz, 4H), 7.55 (d, J = 8.6 Hz, 4H), 7.45 (d, J = 8.6 Hz, 4H), 6.97 (d, J = 8.6 Hz, 4H), 6.94 (d, J = 8.6 Hz, 4H), 4.33 (t, J = 6.6 Hz, 4H), 3.97 (t, J = 6.6 Hz, 6H), 3.85 (d, J = 5.8 Hz, 2H), 3.64 (m, 2H), 1.87–1.77 (m, 11H), 1.58–1.52 (m, 2H), 1.46–1.20 (m, 61H), 0.89–0.86 (m, 6H). ^{13}C NMR (125 MHz, $CDCl_3$): δ (ppm) 166.71, 166.68, 159.44, 158.65, 158.32, 145.19, 143.92, 130.10, 128.36, 127.62, 126.40, 114.91, 114.77, 71.07, 68.16, 68.12, 65.06, 63.24, 38.00, 32.82, 31.95, 31.86, 31.46, 29.94, 29.69, 29.56, 29.57, 29.51, 29.43, 29.39, 29.33, 29.30, 28.73, 26.85, 26.73, 26.04, 25.94, 25.80, 25.71, 22.67, 14.09. MALDI-TOF mass: calcd. for $C_{84}H_{119}O_9$ $[M + H]^+$: m/z = 1271.83; found: 1271.85.

Compound 59. By a procedure similar to that for **5**, compound **51** was obtained in 95% yield (0.50 g, 0.32 mmol) from **58** (0.50 g, 0.39 mmol), methacryloyl chloride (0.26 mL, 2.7 mmol) and triethylamine (0.38 mL, 2.7 mmol). ^{1}H NMR (500 MHz, $CDCl_3$): δ (ppm) 8.07 (d, J = 8.0 Hz, 4H), 7.61 (d, J = 8.6 Hz, 4H), 7.55 (d, J = 8.6 Hz, 4H), 7.45 (d, J = 8.6 Hz, 4H), 6.97 (d, J = 8.6 Hz, 2H), 6.94 (d, J = 8.6 Hz, 4H), 6.10 (s, 1H), 5.54 (t, J = 1.7 Hz, 1H), 4.33 (t, J = 6.6 Hz, 4H), 4.14 (t, J = 6.6 Hz, 2H), 3.98 (t, J = 6.6 Hz, 6H), 3.86 (d, J = 5.8 Hz, 2H), 1.94 (s, 3H), 1.80–1.78 (m, 11H), 1.70–1.53 (m, 2H), 1.46–1.28 (m, 60H), 0.89–0.86 (m, 6H). ^{13}C NMR (125 MHz, $CDCl_3$): δ (ppm) 167.52, 166.61, 159.37, 158.48, 145.14, 136.53, 133.34, 133.19, 132.15, 130.02, 128.52, 128.27, 127.61, 127.58, 126.36, 125.11, 114.89, 114.73, 114.70, 70.95, 68.07, 68.02, 65.02, 64.78, 37.96, 31.88, 31.85, 31.36, 30.01, 29.68, 29.58, 29.44, 29.41, 29.33, 29.22, 28.73, 28.58, 26.83, 26.80, 26.01, 25.94, 25.69, 22.66, 18.32, 14.10. MALDI-TOF mass: calcd. for $C_{88}H_{123}O_{10}$ $[M + H]^+$: m/z = 1339.90; found: 1339.92.

Synthesis of 64, 69 and 72

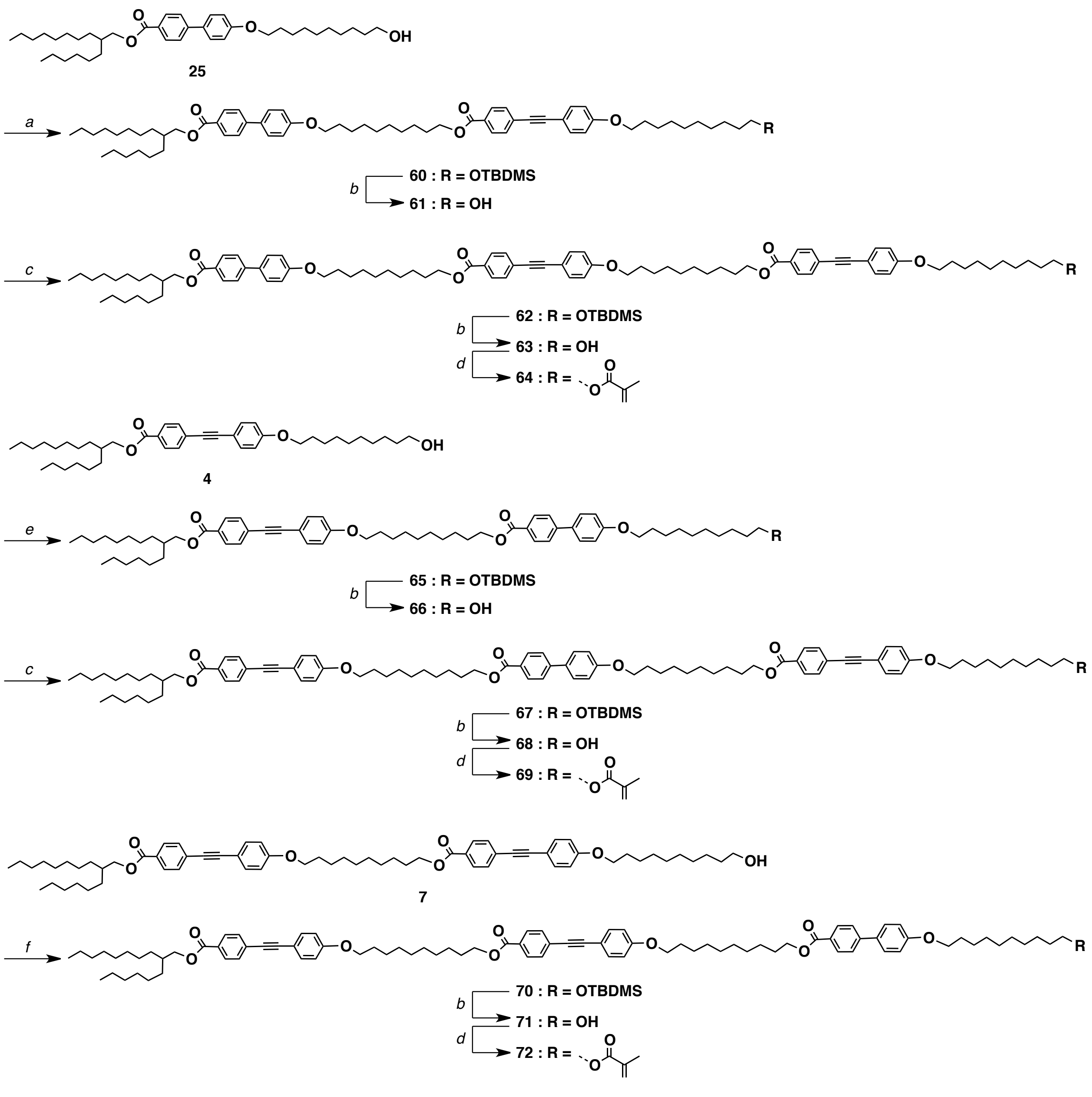

Reagents and conditions: (a) **2**, DPTS, DIPC, CH_2Cl_2, 25 °C; (b) TBAF, THF, 25 °C; (c) **2**, DPTS, DIPC, CH_2Cl_2, 40 °C; (d) methacryloyl chloride, triethylamine, CH_2Cl_2, 25 °C; (e) **23**, DPTS, DIPC, CH_2Cl_2, 25 °C; (f) **23**, DPTS, DIPC, CH_2Cl_2, 40 °C;

Compound 60. By a procedure similar to that for **3**, compound **60** was obtained in 88% yield (1.60 g, 1.48 mmol) from **25** (1.0 g, 1.68 mmol), **2** (0.86 g, 1.70 mmol), DPTS (0.10 g, 0.33 mmol) and DIPC (0.40 g, 3.20 mmol). ^{1}H NMR (500 MHz, $CDCl_3$): δ (ppm) 8.06 (d, J = 8.0 Hz, 2H), 8.00 (d, J = 8.6 Hz, 2H), 7.61 (d, J = 8.0 Hz, 2H), 7.55 (d, J = 8.6 Hz, 4H), 7.46 (d, J = 8.6

Hz, 2H), 6.98 (d, J = 8.6 Hz, 2H), 6.86 (d, J = 8.6 Hz, 2H), 4.30 (t, J = 6.9 Hz, 2H), 4.22 (d, J = 5.8 Hz, 2H), 4.00 (t, J = 6.3 Hz, 2H), 3.96 (t, J = 6.3 Hz, 2H), 3.58 (t, J = 6.6 Hz, 2H), 1.82–1.73 (m, 7H), 1.44–1.20 (m, 50H), 0.88–0.85 (m, 15H), 0.05 (s, 6H). ^{13}C NMR (125 MHz, $CDCl_3$): δ (ppm) 166.68, 166.17, 159.61, 159.38, 158.98, 145.15, 138.12, 133.31, 132.20, 131.23, 130.02, 129.43, 128.59, 128.34, 128.28, 126.40, 116.90, 114.90, 114.58, 114.47, 87.45, 68.07, 67.66, 65.22, 63.30, 37.47, 32.88, 31.90, 31.82, 31.46, 29.97, 29.70, 29.63, 29.56, 29.50, 29.45, 29.41, 29.35, 29.31, 29.25, 29.22, 29.17, 29.12, 28.67, 26.77, 26.75, 26.04, 26.00, 25.98, 25.64, 22.67, 22.65, 18.37, 14.10, −5.27. MALDI-TOF mass: calcd. for $C_{70}H_{105}O_7Si$ $[M + H]^+$: m/z = 1085.66; found: 1085.66.

Compound 61. By a procedure similar to that for **4**, compound **61** was obtained in 95% yield (1.28 g, 1.31 mmol) from **60** (1.50 g, 1.38 mmol) and TBAF (1.0 M in THF, 2.60 mL, 2.60 mmol). ^{1}H NMR (500 MHz, $CDCl_3$): δ (ppm) 8.06 (d, J = 8.0 Hz, 2H), 8.00 (d, J = 8.6 Hz, 2H), 7.61 (d, J = 8.0 Hz, 2H), 7.55 (d, J = 8.6 Hz, 4H), 7.46 (d, J = 8.6 Hz, 2H), 6.98 (d, J = 8.6 Hz, 2H), 6.86 (d, J = 8.6 Hz, 2H), 4.32 (t, J = 6.9 Hz, 2H), 4.23 (d, J = 5.8 Hz, 2H), 4.00 (t, J = 6.3 Hz, 2H), 3.96 (t, J = 6.3 Hz, 2H), 3.64 (t, J = 6.6 Hz, 2H), 1.83–1.72 (m, 7H), 1.59–1.54 (m, 2H), 1.52–1.30 (m, 49H), 0.89–0.86 (m, 6H). ^{13}C NMR (125 MHz, $CDCl_3$): δ (ppm) 166.70, 166.17, 159.57, 159.36, 145.13, 138.10, 133.19, 132.17, 131.21, 129.99, 129.41, 128.55, 128.30, 128.26, 126.38, 116.88, 114.89, 114.57, 114.45, 92.61, 87.44, 68.07, 68.04, 67.65, 65.22, 63.02, 37.44, 32.76, 31.87, 31.79, 31.44, 29.93, 29.60, 29.53, 29.48, 29.43, 29.39, 29.37, 29.32, 29.28, 29.22, 29.20, 29.13, 28.66, 26.74, 26.72, 26.01, 25.96, 25.70, 23.46, 22.64, 14.09. MALDI-TOF mass: calcd. for $C_{64}H_{91}O_7$ $[M + H]^+$: m/z = 971.67; found: 971.62.

Compound 62. By a procedure similar to that for **3**, except that the reaction temperature was 40 °C, compound **62** was obtained in 85% yield (1.24 g, 0.85 mmol) from **61** (1.0 g, 1.0 mmol), **2** (0.55 g, 1.10 mmol), DPTS (0.061 g, 0.21 mmol) and DIPC (0.25 g, 2.01 mmol). ^{1}H NMR (500 MHz, $CDCl_3$): δ (ppm) 8.06 (d, J = 8.6 Hz, 2H), 8.00 (d, J = 8.0 Hz, 4H), 7.61 (d, J = 8.6 Hz, 2H), 7.54 (d, J = 8.6 Hz, 6H), 7.46 (d, J = 8.6 Hz, 2H), 7.46 (d, J = 8.6 Hz, 2H), 6.98 (d, J = 8.6 Hz, 2H), 6.87 (d, J = 8.0 Hz, 4H), 4.32 (t, J = 6.6 Hz, 4H), 4.23 (d, J = 5.8 Hz, 2H), 4.02 (t, J = 6.6 Hz, 2H), 3.96 (t, J = 6.6 Hz, 4H), 3.58 (t, J = 6.6 Hz, 2H), 1.86–1.77 (m, 11H), 1.51–1.28 (m, 62H), 0.89–0.86 (m, 15H), 0.05 (s, 6H). ^{13}C NMR (125 MHz, $CDCl_3$): δ (ppm) 166.65, 166.15, 159.61, 159.48, 145.11, 138.10, 133.31, 132.18, 130.12, 129.48, 128.59, 128.36, 128.28, 126.40, 114.96, 114.68, 114.41, 87.47, 68.12, 67.68, 65.22, 63.34, 37.47, 32.86, 31.93, 31.84, 31.43, 29.95, 29.70, 29.64, 29.56, 29.52, 29.47, 29.41, 29.36, 29.33, 29.25, 29.22, 29.16, 29.12, 28.67, 26.77, 26.74, 26.04, 26.01, 25.98, 25.68, 22.67, 22.63, 18.37, 14.11, −5.27. MALDI-TOF mass: calcd. for $C_{95}H_{133}O_{10}Si$ $[M + H]^+$: m/z = 1461.96; found: 1461.97.

Compound 63. By a procedure similar to that for **4**, compound **63** was obtained in 90% yield (1.00 g, 0.74 mmol) from **62** (1.20 g, 0.82 mmol) and TBAF (1.0 M in THF, 2.0 mL, 2.0 mmol). ^{1}H NMR (500 MHz, $CDCl_3$): δ (ppm) 8.06 (d, J = 8.6 Hz, 2H), 8.00 (d, J = 8.0 Hz, 4H), 7.61 (d, J = 8.6 Hz, 2H), 7.54 (d, J = 8.6 Hz, 6H), 7.46 (d, J = 8.6 Hz, 2H), 7.46 (d, J = 8.6 Hz, 2H), 6.98 (d, J = 8.6 Hz, 2H), 6.87 (d, J = 8.0 Hz, 4H), 4.33 (t, J = 6.6 Hz, 4H), 4.23 (d, J = 5.8 Hz, 2H), 4.02 (t, J = 6.6 Hz, 2H), 3.96 (t, J = 6.6 Hz, 4H), 3.64 (m, 2H), 1.85–1.76 (m, 11H), 1.56–1.53 (m, 2H), 1.46–1.21 (m, 61H), 0.88–0.86 (m, 6H). ^{13}C NMR (125 MHz, $CDCl_3$): δ (ppm) 166.71, 166.21, 159.57, 145.23, 138.14, 133.19, 132.18, 129.97, 129.41, 128.55, 128.32, 128.25, 126.38, 116.88, 114.89, 114.54, 114.46, 92.64, 87.47, 68.11, 68.06, 67.65, 65.21, 63.02, 37.46, 32.76, 31.87, 31.81, 31.44, 29.93, 29.62, 29.53, 29.49, 29.45, 29.39, 29.38, 29.32, 29.25, 29.21, 29.17, 29.14, 28.66, 26.74, 26.72, 26.07, 25.96, 25.74, 23.46, 22.65, 14.09. MALDI-TOF mass: calcd. for $C_{89}H_{119}O_{10}$ $[M + H]^+$: m/z = 1347.87; found: 1347.86.

Compound 64. By a procedure similar to that for **5**, compound **64** was obtained in 60% yield (0.63 g, 0.44 mmol) from **63** (1.00 g, 0.74 mmol), methacryloyl chloride (0.15 g, 1.50 mmol) and triethylamine (0.16 g, 1.50 mmol). ^{1}H NMR (500 MHz, $CDCl_3$): δ (ppm) 8.06 (d, J = 8.6 Hz, 2H), 8.00 (d, J = 8.0 Hz, 4H), 7.61 (d, J = 8.6 Hz, 2H), 7.54 (d, J = 8.6 Hz, 6H), 7.46 (d, J = 8.6 Hz, 2H), 7.46 (d, J = 8.6 Hz, 2H), 6.98 (d, J = 8.6 Hz, 2H), 6.87 (d, J = 8.0 Hz, 4H), 6.09 (s, 1H), 5.54 (t, J = 1.7 Hz, 1H), 4.32 (t, J = 6.6 Hz, 4H), 4.23 (d, J = 5.8 Hz, 2H), 4.14 (t, J = 6.3 Hz, 2H), 4.01 (t, J = 6.3 Hz, 2H), 3.97 (t, J = 6.6 Hz, 4H), 1.94 (s, 3H), 1.80−1.75 (m, 11H), 1.67−1.64 (m, 2H), 1.55−1.28 (m, 60H), 0.88–0.86 (m, 6H). ^{13}C NMR (125 MHz, $CDCl_3$): δ (ppm) 167.51, 159.60, 159.39, 136.61, 136.51, 133.20, 131.23, 130.01, 129.48, 129.42, 128.51, 128.30, 128.27, 126.36, 125.12, 123.38, 117.84, 114.89, 114.57, 114.47, 92.60, 87.46, 68.07, 68.05, 67.88, 65.23, 65.01, 64.78, 37.41, 31.87, 31.79, 31.42, 29.92, 29.59, 29.53, 29.42, 29.40, 29.31, 29.28, 29.22, 29.15, 28.72, 28.67, 28.57, 26.73, 26.71, 26.01, 25.98, 25.93, 22.65, 22.62, 18.32, 14.09. MALDI-TOF mass: calcd. for $C_{93}H_{123}O_{11}$ $[M + H]^+$: m/z = 1415.91; found: 1415.91.

Compound 65. By a procedure similar to that for **3**, compound **60** was obtained in 85% yield (1.55 g, 1.43 mmol) from **4** (1.0 g, 1.68 mmol), **23** (0.82 g, 1.70 mmol), DPTS (0.10 g, 0.33 mmol) and DIPC (0.40 g, 3.20 mmol). ^{1}H NMR (500 MHz, $CDCl_3$): δ (ppm) 8.06 (d, J = 8.0 Hz, 2H), 8.00 (d, J = 8.6 Hz, 2H), 7.62 (d, J = 8.0 Hz, 2H), 7.55 (d, J = 8.6 Hz, 4H), 7.46 (d, J = 8.6 Hz, 2H), 6.98 (d, J = 8.6 Hz, 2H), 6.86 (d, J = 8.6 Hz, 2H), 4.32 (t, J = 6.9 Hz, 2H), 4.23 (d, J = 5.8 Hz, 2H), 4.00 (t, J = 6.3 Hz, 2H), 3.96 (t, J = 6.3 Hz, 2H), 3.58 (t, J = 6.6 Hz, 2H), 1.81–1.72 (m, 7H), 1.45–1.21 (m, 50H), 0.88–0.86 (m, 15H), 0.05 (s, 6H). ^{13}C NMR (125 MHz, $CDCl_3$): δ (ppm) 166.69, 166.17, 159.61, 159.37, 158.98, 145.15, 138.11, 133.31, 132.20, 131.23, 130.02, 129.43, 128.59, 128.34, 128.29, 126.40, 116.90, 114.90, 114.58, 114.47, 87.45, 68.07, 67.66, 65.22, 63.30, 37.48, 32.88, 31.90, 31.82, 31.46, 29.98, 29.70, 29.63, 29.56, 29.50, 29.45, 29.41,

29.35, 29.31, 29.25, 29.22, 29.18, 29.13, 28.67, 26.79, 26.76, 26.04, 26.00, 25.96, 25.64, 22.67, 22.65, 18.32, 14.11, −5.27. MALDI-TOF mass: calcd. for $C_{70}H_{105}O_7Si$ $[M + H]^+$: m/z = 1085.66; found: 1085.65.

Compound 66. By a procedure similar to that for **4**, compound **65** was obtained in 95% yield (1.28 g, 1.31 mmol) from **65** (1.50 g, 1.38 mmol) and TBAF (1.0 M in THF, 2.60 mL, 2.60 mmol). ^{1}H NMR (500 MHz, $CDCl_3$): δ (ppm) 8.06 (d, J = 8.0 Hz, 2H), 8.00 (d, J = 8.6 Hz, 2H), 7.61 (d, J = 8.0 Hz, 2H), 7.55 (d, J = 8.6 Hz, 4H), 7.46 (d, J = 8.6 Hz, 2H), 6.98 (d, J = 8.6 Hz, 2H), 6.86 (d, J = 8.6 Hz, 2H), 4.33 (t, J = 6.9 Hz, 2H), 4.23 (d, J = 5.8 Hz, 2H), 4.00 (t, J = 6.3 Hz, 2H), 3.96 (t, J = 6.3 Hz, 2H), 3.64 (t, J = 6.6 Hz, 2H), 1.82−1.70 (m, 7H), 1.58−1.55 (m, 2H), 1.52−1.28 (m, 49H), 0.89−0.86 (m, 6H). ^{13}C NMR (125 MHz, $CDCl_3$): δ (ppm) 166.71, 166.17, 159.56, 159.36, 145.13, 138.11, 133.19, 132.16, 131.21, 129.98, 129.41, 128.56, 128.30, 128.26, 126.38, 116.88, 114.88, 114.54, 114.47, 92.61, 87.44, 68.07, 68.04, 67.65, 65.24, 63.02, 37.44, 32.76, 31.88, 31.79, 31.44, 29.93, 29.61, 29.53, 29.48, 29.43, 29.38, 29.36, 29.32, 29.28, 29.25, 29.20, 29.13, 28.66, 26.71, 26.72, 26.01, 25.96, 25.72, 23.46, 22.64, 14.09. MALDI-TOF mass: calcd. for $C_{64}H_{91}O_7$ $[M + H]^+$: m/z = 971.67; found: 971.65.

Compound 67. By a procedure similar to that for **3**, except that the reaction temperature was 40 °C, compound **67** was obtained in 87% yield (1.27 g, 0.87 mmol) from **66** (1.0 g, 1.0 mmol), **2** (0.55 g, 1.10 mmol), DPTS (0.061 g, 0.21 mmol) and DIPC (0.25 g, 2.01 mmol). ^{1}H NMR (500 MHz, $CDCl_3$): δ (ppm) 8.06 (d, J = 8.6 Hz, 2H), 8.00 (d, J = 8.0 Hz, 4H), 7.61 (d, J = 8.6 Hz, 2H), 7.54 (d, J = 8.6 Hz, 6H), 7.46 (d, J = 8.6 Hz, 2H), 7.46 (d, J = 8.6 Hz, 2H), 6.98 (d, J = 8.6 Hz, 2H), 6.87 (d, J = 8.0 Hz, 4H), 4.33 (t, J = 6.6 Hz, 4H), 4.23 (d, J = 5.8 Hz, 2H), 4.00 (t, J = 6.6 Hz, 2H), 3.96 (t, J = 6.6 Hz, 4H), 3.58 (t, J = 6.6 Hz, 2H), 1.87−1.78 (m, 11H), 1.52−1.28 (m, 62H), 0.89−0.86 (m, 15H), 0.05 (s, 6H). ^{13}C NMR (125 MHz, $CDCl_3$): δ (ppm) 166.65, 166.15, 159.64, 159.48, 145.11, 138.13, 133.31, 132.16, 130.12, 129.48, 128.58, 128.36, 128.28, 126.42, 114.96, 114.68, 114.46, 87.47, 68.12, 67.68, 65.22, 63.34, 37.47, 32.87, 31.93, 31.84, 31.46, 29.95, 29.77, 29.64, 29.56, 29.51, 29.47, 29.40, 29.36, 29.33, 29.25, 29.25, 29.16, 29.14, 28.67, 26.78, 26.74, 26.05, 26.01, 25.98, 25.68, 22.63, 22.63, 18.37, 14.11, −5.27. MALDI-TOF mass: calcd. for $C_{95}H_{133}O_{10}Si$ $[M + H]^+$: m/z = 1461.96; found: 1461.97.

Compound 68. By a procedure similar to that for **4**, compound **67** was obtained in 95% yield (1.01 g, 0.75 mmol) from **62** (1.20 g, 0.82 mmol) and TBAF (1.0 M in THF, 2.0 mL, 2.0 mmol). ^{1}H NMR (500 MHz, $CDCl_3$): δ (ppm) 8.06 (d, J = 8.6 Hz, 2H), 8.00 (d, J = 8.0 Hz, 4H), 7.61 (d, J = 8.6 Hz, 2H), 7.54 (d, J = 8.6 Hz, 6H), 7.46 (d, J = 8.6 Hz, 2H), 7.46 (d, J = 8.6 Hz, 6H), 6.98 (d, J = 8.6 Hz, 2H), 6.87 (d, J = 8.0 Hz, 4H), 4.32 (t, J = 6.6 Hz, 4H), 4.23 (d, J = 5.8 Hz, 2H), 4.01 (t, J = 6.6 Hz, 2H), 3.96 (t, J = 6.6 Hz, 4H), 3.64 (br, J = 6.6 Hz, 2H), 1.86−1.77 (m, 11H), 1.57−1.53 (m, 2H), 1.46−1.20 (m, 61H), 0.88−0.86 (m, 6H). ^{13}C NMR (125 MHz, $CDCl_3$): δ

(ppm) 166.71, 166.21, 159.58, 145.23, 138.16, 133.19, 132.18, 129.97, 129.45, 128.55, 128.31, 128.25, 126.38, 116.88, 114.85, 114.54, 114.47, 92.64, 87.47, 68.12, 68.06, 67.65, 65.21, 63.12, 37.46, 32.76, 31.88, 31.81, 31.46, 29.93, 29.62, 29.56, 29.49, 29.40, 29.38, 29.36, 29.32, 29.25, 29.20, 29.17, 29.13, 28.66, 26.74, 26.72, 26.07, 25.98, 25.74, 23.46, 22.61, 14.10. MALDI-TOF mass: calcd. for $C_{89}H_{119}O_{10}$ $[M + H]^+$: m/z = 1347.87; found: 1347.82.

Compound 69. By a procedure similar to that for **5**, compound **69** was obtained in 70% yield (0.73 g, 0.52 mmol) from **69** (1.00 g, 0.74 mmol), methacryloyl chloride (0.15 g, 1.50 mmol) and triethylamine (0.16 g, 1.50 mmol). ^{1}H NMR (500 MHz, $CDCl_3$): δ (ppm) 8.07 (d, J = 8.6 Hz, 2H), 8.00 (d, J = 8.0 Hz, 2H), 8.00 (d, J = 8.0 Hz, 2H), 7.61 (d, J = 8.6 Hz, 2H), 7.55 (d, J = 8.6 Hz, 6H), 7.46 (d, J = 8.6 Hz, 2H), 7.46 (d, J = 8.6 Hz, 2H), 6.98 (d, J = 8.6 Hz, 2H), 6.87 (d, J = 8.0 Hz, 4H), 6.10 (s, 1H), 5.54 (t, J = 1.7 Hz, 1H), 4.32 (t, J = 6.6 Hz, 4H), 4.23 (d, J = 5.8 Hz, 2H), 4.14 (t, J = 6.3 Hz, 2H), 4.01 (t, J = 6.3 Hz, 2H), 3.97 (t, J = 6.6 Hz, 4H), 1.94 (s, 3H), 1.82–1.76 (m, 11H), 1.69–1.64 (m, 2H), 1.50–1.28 (m, 60H), 0.89–0.87 (m, 6H). ^{13}C NMR (125 MHz, $CDCl_3$): δ (ppm) 167.58, 167.23, 159.64, 159.39, 136.62, 136.51, 133.21, 131.23, 130.01, 129.48, 129.42, 128.51, 128.33, 128.27, 126.36, 125.18, 123.38, 117.86, 114.89, 114.53, 114.47, 92.61, 87.46, 68.07, 68.07, 67.88, 65.26, 65.01, 64.78, 37.41, 31.89, 31.79, 31.42, 29.90, 29.59, 29.53, 29.42, 29.40, 29.33, 29.28, 29.22, 29.17, 28.72, 28.65, 28.57, 26.76, 26.71, 26.01, 25.98, 25.95, 22.65, 22.64, 18.32, 14.10. MALDI-TOF mass: calcd. for $C_{93}H_{123}O_{11}$ $[M + H]^+$: m/z = 1415.91; found: 1415.94.

Compound 70. By a procedure similar to that for **3**, except that the reaction temperature was 40 °C, compound **70** was obtained in 91% yield (1.33 g, 0.91 mmol) from **7** (1.0 g, 1.0 mmol), **23** (0.53 g, 1.10 mmol), DPTS (0.060 g, 0.210 mmol) and DIPC (0.25 g, 1.98 mmol). ^{1}H NMR (500 MHz, $CDCl_3$): δ (ppm) 8.06 (d, J = 8.6 Hz, 2H), 8.00 (d, J = 8.0 Hz, 4H), 7.61 (d, J = 8.6 Hz, 2H), 7.54 (d, J = 8.6 Hz, 6H), 7.46 (d, J = 8.6 Hz, 2H), 7.46 (d, J = 8.6 Hz, 2H), 6.98 (d, J = 8.6 Hz, 2H), 6.87 (d, J = 8.0 Hz, 4H), 4.32 (t, J = 6.6 Hz, 4H), 4.23 (d, J = 5.8 Hz, 2H), 4.00 (t, J = 6.6 Hz, 2H), 3.96 (t, J = 6.6 Hz, 4H), 3.58 (t, J = 6.6 Hz, 2H), 1.85–1.78 (m, 11H), 1.53–1.29 (m, 62H), 0.89–0.86 (m, 15H), 0.05 (s, 6H). ^{13}C NMR (125 MHz, $CDCl_3$): δ (ppm) 166.68, 166.17, 159.61, 159.48, 145.10, 138.11, 133.31, 132.19, 129.48, 128.59, 128.36, 128.28, 126.40, 114.96, 114.68, 114.44, 87.47, 68.20, 67.68, 65.24, 63.34, 37.47, 32.86, 31.96, 31.84, 31.43, 29.95, 29.71, 29.64, 29.57, 29.52, 29.47, 29.41, 29.37, 29.33, 29.25, 29.23, 29.16, 29.12, 28.65, 26.81, 26.78, 26.04, 26.01, 25.96, 25.68, 22.65, 22.63, 18.31, 14.11, −5.26. MALDI-TOF mass: calcd. for $C_{95}H_{133}O_{10}Si$ $[M + H]^+$: m/z = 1461.96; found: 1461.93.

Compound 71. By a procedure similar to that for **4**, compound **71** was obtained in 94% yield (1.13 g, 0.84 mmol) from **70** (1.30 g, 0.89 mmol) and TBAF (1.0 M in THF, 2.0 mL, 2.0 mmol). ^{1}H NMR (500 MHz, $CDCl_3$): δ (ppm) 8.06 (d, J = 8.6 Hz, 2H), 8.00 (d, J = 8.0 Hz, 4H), 7.61 (d,

J = 8.6 Hz, 2H), 7.54 (d, J = 8.6 Hz, 6H), 7.46 (d, J = 8.6 Hz, 2H), 7.46 (d, J = 8.6 Hz, 2H), 6.98 (d, J = 8.6 Hz, 2H), 6.87 (d, J = 8.0 Hz, 4H), 4.32 (t, J = 6.6 Hz, 4H), 4.23 (d, J = 5.8 Hz, 2H), 4.00 (t, J = 6.6 Hz, 2H), 3.96 (t, J = 6.6 Hz, 4H), 3.64 (br, J = 6.6 Hz, 2H), 1.86–1.77 (m, 11H), 1.58–1.52 (m, 2H), 1.46–1.23 (m, 61H), 0.89–0.86 (m, 6H). ^{13}C NMR (125 MHz, $CDCl_3$): δ (ppm) 166.73, 166.25, 159.56, 145.23, 138.13, 133.19, 132.16 129.97, 129.45, 128.55, 128.32, 128.25, 126.38, 116.88, 114.89, 114.54, 114.46, 92.64, 87.47, 68.11, 68.06, 67.65, 65.21, 63.21, 37.45, 32.76, 31.87, 31.83, 31.44, 29.93, 29.67, 29.52, 29.49, 29.43, 29.35, 29.32, 29.30, 29.26, 29.24, 29.17, 29.14, 28.67, 26.73, 26.72, 26.07, 25.94, 25.74, 23.47, 22.65, 14.11. MALDI-TOF mass: calcd. for $C_{89}H_{119}O_{10}$ $[M + H]^+$: m/z = 1347.87; found: 1347.85.

Compound 72. By a procedure similar to that for **5**, compound **72** was obtained in 65% yield (0.68 g, 0.48 mmol) from **71** (1.0 g, 0.74 mmol), methacryloyl chloride (0.14 g, 1.30 mmol) and triethylamine (0.14 g, 1.36 mmol). ^{1}H NMR (500 MHz, $CDCl_3$): δ (ppm) 8.07 (d, J = 8.6 Hz, 2H), 8.00 (d, J = 8.0 Hz, 2H), 8.00 (d, J = 8.0 Hz, 2H), 7.61 (d, J = 8.6 Hz, 2H), 7.55 (d, J = 8.6 Hz, 6H), 7.46 (d, J = 8.6 Hz, 2H), 7.46 (d, J = 8.6 Hz, 2H), 6.97 (d, J = 8.6 Hz, 2H), 6.87 (d, J = 8.0 Hz, 4H), 6.10 (s, 1H), 5.54 (t, J = 1.7 Hz, 1H), 4.33 (t, J = 6.6 Hz, 4H), 4.23 (d, J = 5.8 Hz, 2H), 4.14 (t, J = 6.3 Hz, 2H), 4.01 (t, J = 6.3 Hz, 2H), 3.97 (t, J = 6.6 Hz, 4H), 1.94 (s, 3H), 1.83–1.76 (m, 11H), 1.70–1.64 (m, 2H), 1.50–1.28 (m, 60H), 0.89–0.86 (m, 6H). ^{13}C NMR (125 MHz, $CDCl_3$): δ (ppm) 167.52, 167.48, 159.61, 159.38, 136.61, 136.54, 133.20, 131.25, 130.01, 129.48, 129.42, 128.51, 128.30, 128.27, 126.36, 125.12, 123.38, 117.84, 114.89, 114.57, 114.47, 92.60, 87.45, 68.07, 68.05, 67.86, 65.23, 65.05, 64.78, 37.41, 31.86, 31.79, 31.47, 29.91, 29.59, 29.53, 29.45, 29.41, 29.31, 29.28, 29.20, 29.17, 28.72, 28.68, 28.57, 26.78, 26.75, 26.01, 25.98, 25.93, 22.65, 22.62, 18.31, 14.09. MALDI-TOF mass: calcd. for $C_{93}H_{123}O_{11}$ $[M + H]^+$: m/z = 1415.91; found: 1415.88.

Polymerization of 5 (PMA^{T}). Monomer **5** (0.31 g, 0.45 mmol) was placed in a Schlenk flask (25 mL), and the inner atmosphere was strictly purged with Ar. A stock solution of AIBN (13.3 mM) in anhydrous benzene was degassed by freeze-pump-thaw cycles (three times). This stock solution (0.45 mL) was introduced using a syringe into the flack containing **5**, and the mixture was stirred at 70 °C for 24 h. The reaction mixture was subjected to preparative SEC with $CHCl_3$ as an eluent, to allow separation of a polymeric fraction from **5**. The polymeric fraction was dried at 50 °C under a reduced pressure to give **PMA^{T}** (295 mg) as colorless oil in 95% yield. ^{1}H NMR (500 MHz, $CDCl_3$): δ (ppm) 8.00–7.91 (m), 7.55–7.38 (m), 6.87–6.77 (m), 4.21–4.17 (m), 4.01–3.85 (br), 1.78–1.68 (br), 1.60–1.54 (br), 1.48–1.18 (br), 0.88–0.81 (m). FT-IR (ATR): ν (cm^{-1}) 2927, 2853, 2212, 1715, 1601, 1514, 1468, 1400, 1273, 1251, 1174, 1142, 1107, 1018. SEC analysis ($CHCl_3$, polystyrene standards): number-average molecular weight (M_n) = 7.0 × 10^4 g mol^{-1}, polydispersity index (M_w/M_n) = 4.0.

Polymerization of 8 (PMA^{TT}). Monomer **8** (0.32 g, 0.30 mmol) was placed in a Schlenk flask (25 mL), and the inner atmosphere was strictly purged with Ar. A stock solution of AIBN (8.92 mM) in anhydrous benzene was degassed by freeze-pump-thaw cycles (three times). This stock solution (0.45 mL) was introduced using a syringe into the flack containing **8**, and the mixture was stirred at 70 °C. After 24 h, the reaction mixture was poured into MeOH (150 mL), and a white precipitate formed was collected by filtration and subjected to preparative SEC with $CHCl_3$ as an eluent, to allow separation of a polymeric fraction from **8**. The polymeric fraction was concentrated (~5 mL) under a reduced pressure to a small volume, which was then added dropwise to MeOH (150 mL). A precipitate thus formed was collected by filtration and dried at 25 °C under a reduced pressure to give **PMA^{TT}** (282 mg) as white solid in 88% yield. ^{1}H NMR (500 MHz, $CDCl_3$): δ (ppm) 8.04–7.94 (m), 7.58–7.38 (m), 6.88–6.76 (m), 4.31–4.17 (br), 3.98–3.85 (br), 1.81–1.70 (br), 1.68–1.55 (br), 1.48–1.18 (br), 0.88–0.84 (m). FT-IR (ATR): ν (cm^{-1}) 2928, 2852, 2212, 1714, 1598, 1514, 1467, 1402, 1273, 1250, 1176, 1140, 1107, 1018. SEC analysis ($CHCl_3$, polystyrene standards): M_n = 7.6 × 10^4 g mol^{-1}, M_w/M_n = 3.1.

Polymerization of 11 (PMA^{TTT}). By a procedure similar to that for **PMA^{TT}**, **PMA^{TTT}** was obtained in 80% yield (192 mg) from monomer **11** (0.24 g, 0.17 mmol) and AIBN in anhydrous benzene (6.13 mM, 0.36 mL). ^{1}H NMR (500 MHz, $CDCl_3$): δ (ppm) 8.03–7.93 (m), 7.57–7.38 (m), 6.89–6.77 (m), 4.33–4.21 (br), 3.98–3.86 (m), 1.81–1.68 (br), 1.67–1.55 (br), 1.48–1.18 (br), 0.88–0.84 (m). FT-IR (ATR): ν (cm^{-1}) 2927, 2852, 2212, 1714, 1599, 1514, 1468, 1400, 1273, 1250, 1174, 1140, 1107, 1018. SEC analysis ($CHCl_3$, polystyrene standards): M_n = 7.7 × 10^4 g mol^{-1}, M_w/M_n = 3.0.

Polymerization of 17 (PMA^{TTA}). By a procedure similar to that for **PMA^{TT}**, **PMA^{TTA}** was obtained in 85% yield (170 mg) from monomer **17** (0.20 g, 0.14 mmol) and AIBN in anhydrous benzene (6.13 mM, 0.30 mL). ^{1}H NMR (500 MHz, $CDCl_3$): δ (ppm) 8.16–8.12 (m), 8.02–7.86 (m), 7.56–7.39 (m), 7.05–6.97 (m), 6.87–6.78 (m), 4.32–4.21 (br), 4.05–3.98 (m), 3.97–3.84 (m), 1.81–1.68 (br), 1.67–1.55 (br), 1.48–1.18 (br), 0.90–0.84 (m). FT-IR (ATR): ν (cm^{-1}) 2927, 2854, 2212, 1716, 1601, 1516, 1469, 1402, 1275, 1174, 1140, 1109, 1016. SEC analysis ($CHCl_3$, polystyrene standards): $M_n = 7.9 \times 10^4$ g mol^{-1}, $M_w/M_n = 3.3$.

Polymerization of 21 (PMA^{TAA}). By a procedure similar to that for **PMA^{TT}**, **PMA^{TAA}** was obtained in 75% yield (165 mg) from monomer **21** (0.22 g, 0.15 mmol) and AIBN in anhydrous benzene (6.13 mM, 0.33 mL). ^{1}H NMR (500 MHz, $CDCl_3$): δ (ppm) 8.15–8.12 (m), 7.98–7.86 (m), 7.56–7.38 (br), 7.01–6.93 (m), 6.83–6.78 (br), 4.35–4.21 (br), 4.05–3.94 (m), 3.93–3.81 (br), 1.81–1.68 (br), 1.67–1.55 (br), 1.48–1.18 (br), 0.90–0.84 (m). FT-IR (ATR): ν (cm^{-1}) 2927, 2852, 2212, 1716, 1599, 1502, 1469, 1400, 1275, 1174, 1138, 1107, 1016. SEC analysis ($CHCl_3$, polystyrene standards): $M_n = 6.2 \times 10^4$ g mol^{-1}, $M_w/M_n = 2.2$.

Polymerization of 26 (PMA^{B}). By a procedure similar to that for **PMA^{T}**, **PMA^{B}** was obtained in 92% yield (276 mg) from monomer **26** (0.30 g, 0.45 mmol) and AIBN in anhydrous benzene (13.3 mM, 0.45 mL). ^{1}H NMR (500 MHz, $CDCl_3$): δ (ppm) 8.05–7.98 (m), 7.60–7.45 (m), 6.97–6.87 (br), 4.23–4.18 (m), 4.03–3.87 (br), 1.80–1.68 (br), 1.72–1.51 (br), 1.48–1.18 (br), 0.91–0.82 (m). FT-IR (ATR): ν (cm^{-1}) 2927, 2854, 1714, 1605, 1525, 1496, 1472, 1396, 1275, 1191, 1109, 1039. SEC analysis ($CHCl_3$, polystyrene standards): $M_n = 5.1 \times 10^4$ g mol^{-1}, $M_w/M_n = 3.8$.

Polymerization of 29 (PMA^{BB}). By a procedure similar to that for **PMA^{TT}**, **PMA^{BB}** was obtained in 90% yield (270 mg) from monomer **29** (0.30 g, 0.30 mmol) and AIBN in anhydrous benzene (8.91 mM, 0.45 mL). ^{1}H NMR (500 MHz, $CDCl_3$): δ (ppm) 8.08–7.96 (m), 7.62–7.42 (m), 6.96–6.85 (m), 4.31–4.20 (br), 3.98–3.86 (br), 1.81–1.70 (br), 1.68–1.55 (br), 1.48–1.18 (br), 0.88–0.83 (m). FT-IR (ATR): ν (cm^{-1}) 2927, 2854, 1715, 1605, 1525, 1497, 1475, 1396, 1276, 1191, 1109, 1039. SEC analysis ($CHCl_3$, polystyrene standards): $M_n = 1.6 \times 10^5$ g mol^{-1}, $M_w/M_n = 3.4$.

Polymerization of 32 (PMA^{BBB}). By a procedure similar to that for **PMA^{TT}**, **PMA^{BBB}** was obtained in 90% yield (225 mg) from monomer **32** (0.25 g, 0.18 mmol) and AIBN in anhydrous benzene (6.13 mM, 0.40 mL). ^{1}H NMR (500 MHz, $CDCl_3$): δ (ppm) 8.08–7.96 (m), 7.62–7.42 (m), 6.96–6.85 (m), 4.31–4.20 (br), 3.98–3.86 (br), 1.81–1.70 (br), 1.68–1.55 (br), 1.48–1.18 (br), 0.90–0.83 (m). FT-IR (ATR): ν (cm^{-1}) 2924, 2854, 1714, 1604, 1525, 1496, 1471, 1396,

1275, 1190, 1109, 1039. SEC analysis ($CHCl_3$, polystyrene standards): M_n = 7.6 × 10^4 g mol^{-1}, M_w/M_n = 3.8.

Polymerization of 33 (PA^{BBB}). By a procedure similar to that for **PMA^{TT}**, **PA^{BBB}** was obtained in 47% yield (94 mg) from monomer **33** (0.20 g, 0.15 mmol) and AIBN in anhydrous benzene (6.13 mM, 0.32 mL). ^{1}H NMR (500 MHz, $CDCl_3$): δ (ppm) 8.03 (br), 7.59–7.51 (br), 6.94 (br), 4.28 (br), 4.20–4.10 (br), 3.94 (br), 1.78–1.74 (br), 1.53–1.25 (br), 0.86 (br). FT-IR (ATR): ν (cm^{-1}) 2925, 2854, 1716, 1603, 1525, 1498, 1472, 1396, 1275, 1191, 1108, 1039. SEC analysis ($CHCl_3$, polystyrene standards): M_n = 3.8 × 10^4 g mol^{-1}, M_w/M_n = 1.4.

Polymerization of 34 (PPA^{BBB}). Monomer **34** (0.20 g, 0.14 mmol) was placed in a Schlenk flask (25 mL), and the inner atmosphere was strictly purged with Ar. A stock solution of Rh(nbd)BPh_4 (1.2 mM) in anhydrous $CHCl_3$ was degassed by freeze-pump-thaw cycles (three times). This stock solution (2.3 mL) was introduced using a syringe into the flask containing **34**, and the mixture was stirred at 25 °C. After 24 h, triphenylphosphine (7.9 mg, 0.03 mmol) was added into the reaction mixture. The mixture was poured into MeOH, and a precipitate formed was collected by filtration and reprecipitated twice from MeOH. A precipitate thus formed was dried at 25 °C under a reduced pressure, and then subjected to preparative SEC with $CHCl_3$ as an eluent, to allow separation of a polymeric fraction from **34** as yellow solid (140 mg) in 70% yield. ^{1}H NMR (500 MHz, $CDCl_3$): δ (ppm) 8.06–7.94 (m), 7.62–7.41 (m), 6.96–6.86 (m), 6.72 (br), 5.74 (br), 4.30–4.23 (br), 3.98–3.84 (br), 1.82–1.65 (br), 1.48–1.20 (br), 0.90–0.83 (m). FT-IR (ATR): ν (cm^{-1}) 2925, 2854, 1714, 1604, 1525, 1496, 1469, 1396, 1277, 1188, 1108, 1039. SEC analysis ($CHCl_3$, polystyrene standards): M_n = 6.2 × 10^4 g mol^{-1}, M_w/M_n = 2.0.

Polymerization of 39 (PMA^{BBA}). By a procedure similar to that for **PMA^{TT}**, **PMA^{BBA}** was obtained in 87% yield (165 mg) from monomer **39** (0.19 g, 0.14 mmol) and AIBN in anhydrous benzene (6.13 mM, 0.30 mL). ^{1}H NMR (500 MHz, $CDCl_3$): δ (ppm) 8.16–8.12 (m), 8.08–7.86 (m), 7.59–7.42 (m), 7.01–6.96 (m), 6.92–6.86 (m), 4.32–4.22 (br), 4.05–3.98 (m), 3.97–3.86 (br), 1.82–1.68 (br), 1.67–1.55 (br), 1.48–1.18 (br), 0.90–0.84 (m). FT-IR (ATR): ν (cm^{-1}) 2925, 2854, 1714, 1603, 1525, 1498, 1469, 1398, 1275, 1190, 1138, 1109, 1036. SEC analysis ($CHCl_3$, polystyrene standards): M_n = 7.7 × 10^4 g mol^{-1}, M_w/M_n = 2.9.

Polymerization of 42 (PMA^{BAA}). By a procedure similar to that for **PMA^{TT}**, **PMA^{BAA}** was obtained in 89% yield (205 mg) from monomer **42** (0.23 g, 0.15 mmol) and AIBN in anhydrous benzene (6.13 mM, 0.33 mL). ^{1}H NMR (500 MHz, $CDCl_3$): δ (ppm) 8.15–8.12 (m), 8.06–7.85 (m), 7.56–7.43 (br), 7.01–6.93 (m), 6.93–6.85 (br), 4.35–4.21 (br), 3.04–3.82 (br), 1.81–1.68 (br), 1.67–1.55 (br), 1.48–1.18 (br), 0.90–0.84 (m). FT-IR (ATR): ν (cm^{-1}) 2929, 2854, 1716,

1603, 1525, 1500, 1469, 1400, 1273, 1188, 1140, 1111, 1036, 1014. SEC analysis ($CHCl_3$, polystyrene standards): $M_n = 8.0 \times 10^4$ g mol^{-1}, $M_w/M_n = 3.2$.

Polymerization of 51 (PMA$^{TTT'}$). By a procedure similar to that for **PMA^{TT}**, **PMA^{TTT}** was obtained in 80% yield (176 mg) from monomer **51** (0.22 g, 0.16 mmol) and AIBN in anhydrous benzene (4.5 mM, 0.35 mL). ^{1}H NMR (500 MHz, $CDCl_3$): δ (ppm) 7.98–7.96 (m), 7.52–7.49 (m), 7.43–7.39 (m), 6.82 (m), 4.28 (br), 3.91 (br), 3.80 (m), 1.74–1.55 (m), 1.40–1.26 (br), 0.87–0.85 (m). FT-IR (ATR): ν (cm^{-1}) 2925, 2854, 2214, 1714, 1601, 1566, 1516, 1469, 1404, 1281, 1248, 1173, 1142, 1107, 1020. SEC analysis ($CHCl_3$, polystyrene standards): $M_n = 1.2 \times 10^5$ g/mol, $M_w/M_n = 2.7$.

Polymerization of 59 (PMA$^{BBB'}$). By a procedure similar to that for **PMA^{TT}**, **PMA^{BBB}** was obtained in 53% yield (105 mg) from monomer **59** (0.21 g, 0.16 mmol) and AIBN in anhydrous benzene (4.5 mM, 0.35 mL). ^{1}H NMR (500 MHz, $CDCl_3$): δ (ppm) 8.04–8.00 (m), 7.57–7.41 (m), 6.91 (m), 4.29 (br), 3.94 (br), 3.82 (m), 1.76–1.51 (m), 1.42–1.28 (br), 0.86 (m). FT-IR (ATR): ν (cm^{-1}) 2924, 2854, 1714, 1604, 1527, 1498, 1469, 1396, 1281, 1244, 1192, 1111, 1041. SEC analysis ($CHCl_3$, polystyrene standards): $M_n = 7.3 \times 10^4$ g/mol, $M_w/M_n = 2.2$.

Polymerization of 64 (PMA^{TTB}). By a procedure similar to that for **PMA^{TT}**, **PMA^{TTB}** was obtained in 80% yield (184 mg) from monomer **64** (0.23 g, 0.16 mmol) and AIBN in anhydrous benzene (4.5 mM, 0.35 mL). ^{1}H NMR (500 MHz, $CDCl_3$): δ (ppm) 8.04 (d), 7.98–7.96 (br), 7.60–7.41 (m), 6.95 (d), 6.82 (br), 4.28 (br), 4.22 (br), 3.96 (br), 3.90 (br), 1.74 (br), 1.57 (br), 1.40–1.25 (br), 0.87–0.85 (m). FT-IR (ATR): ν (cm^{-1}) 2925, 2854, 2214, 1715, 1600, 1516, 1498, 1469, 1405, 1273, 1249, 1174, 1141, 1016. SEC analysis ($CHCl_3$, polystyrene standards): $M_n = 8.4 \times 10^4$ g/mol, $M_w/M_n = 2.3$.

Polymerization of 69 (PMA^{TBT}). By a procedure similar to that for **PMA^{TT}**, **PMA^{TBT}** was obtained in 67% yield (154 mg) from monomer **69** (0.23 g, 0.16 mmol) and AIBN in anhydrous benzene (4.5 mM, 0.35 mL). ^{1}H NMR (500 MHz, $CDCl_3$): δ (ppm) 8.04 (d), 7.98–7.96 (br), 7.58–7.40 (m), 6.93 (br), 6.84–6.80 (m), 4.29 (br), 4.21 (br), 3.93–3.90 (br), 1.75 (br), 1.58 (br), 1.40–1.25 (br), 0.87–0.85 (m). FT-IR (ATR): ν (cm^{-1}) 2924, 2854, 2213, 1714, 1601, 1518, 1469, 1405, 1274, 1249, 1175, 1141, 1105, 1017. SEC analysis ($CHCl_3$, polystyrene standards): $M_n = 6.5 \times 10^4$ g/mol, $M_w/M_n = 2.0$.

Polymerization of 72 (PMA^{BTT}). By a procedure similar to that for **PMA^{TT}**, **PMA^{BTT}** was obtained in 91% yield (209 mg) from monomer **72** (0.23 g, 0.16 mmol) and AIBN in anhydrous benzene (4.5 mM, 0.35 mL). ^{1}H NMR (500 MHz, $CDCl_3$): δ (ppm) 7.98–7.96 (m), 7.54–7.41 (m), 6.85–6.81 (m), 4.28 (br), 4.21 (br), 3.94–3.89 (m), 1.74 (m), 1.56 (br), 1.40–1.26 (br),

0.87–0.85 (m). FT-IR (ATR): ν (cm^{-1}) 2924, 2854, 2215, 1715, 1600, 1517, 1498, 1469, 1404, 1277, 1250, 1175, 1141, 1107, 1017. SEC analysis ($CHCl_3$, polystyrene standards): M_n = 1.1 × 10^5 g/mol, M_w/M_n = 3.3.

Supplementary References

1. Moore, J. S. & Stupp, S. I. Room temperature polyesterification. *Macromolecules* **23**, 65–70 (1990).

2. Hosono, N. *et al*. Large-area three-dimensional molecular ordering of a polymer brush by one-step processing. *Science* **330**, 808–811 (2010).

3. Stas, S., Sergeyev, S. & Geerts, Y. Synthesis of diketopyrrolopyrrole (DPP) derivatives comprising bithiophene moieties. *Tetrahedron* **66**, 1837–1845 (2010).

4. Yashima, E., Matsushima, T. & Okamoto, Y. Chirality assignment of amines and amino alcohols based on circular dichroism induced by helix formation of a stereoregular poly((4-carboxyphenyl)acetylene) through acid-base complexation. *J. Am. Chem. Soc.* **119**, 6345–6359 (1997).

5. Schrock, R. & Osborn, J. A. π-Bonded complexes of the tetraphenylborate ion with rhodium(I) and iridium(I). *Inorg. Chem.* **9**, 2339–2343 (1970).

6. http://www.esrf.eu/computing/scientific/FIT2D/

7. http://homepage2.nifty.com/~hsc/soft/cellcalc_e.html

8. Frisch, M. J. *et al*. Gaussian 03, Gaussian, Inc., Wallingford CT (2004).

9. Daxer, A. & Fratzl, P. Collagen fibril orientation in the human corneal stroma and its implication in keratoconus. *Invest. Ophthalmol. Vis. Sci.* **38**, 121–129 (1997).
